# Diversity and evolution of the small multidrug resistance protein family

**DOI:** 10.1186/1471-2148-9-140

**Published:** 2009-06-23

**Authors:** Denice C Bay, Raymond J Turner

**Affiliations:** 1 Department of Biological Sciences, University of Calgary, Calgary, Alberta, T2N 1N4, Canada

## Abstract

**Background:**

Members of the small multidrug resistance (SMR) protein family are integral membrane proteins characterized by four α-helical transmembrane strands that confer resistance to a broad range of antiseptics and lipophilic quaternary ammonium compounds (QAC) in bacteria. Due to their short length and broad substrate profile, SMR proteins are suggested to be the progenitors for larger α-helical transporters such as the major facilitator superfamily (MFS) and drug/metabolite transporter (DMT) superfamily. To explore their evolutionary association with larger multidrug transporters, an extensive bioinformatics analysis of SMR sequences (> 300 Bacteria taxa) was performed to expand upon previous evolutionary studies of the SMR protein family and its origins.

**Results:**

A thorough annotation of unidentified/putative SMR sequences was performed placing sequences into each of the three SMR protein subclass designations, namely small multidrug proteins (SMP), suppressor of *groEL *mutations (SUG), and paired small multidrug resistance (PSMR) using protein alignments and phylogenetic analysis. Examination of SMR subclass distribution within Bacteria and Archaea taxa identified specific Bacterial classes that uniquely encode for particular SMR subclass members. The extent of selective pressure acting upon each SMR subclass was determined by calculating the rate of synonymous to non-synonymous nucleotide substitutions using Syn-SCAN analysis. SUG and SMP subclasses are maintained under moderate selection pressure in comparison to integron and plasmid encoded SMR homologues. Conversely, PSMR sequences are maintained under lower levels of selection pressure, where one of the two PSMR pairs diverges in sequence more rapidly than the other. SMR genomic loci surveys identified potential SMR efflux substrates based on its gene association to putative operons that encode for genes regulating amino acid biogenesis and QAC-like metabolites. SMR subclass protein transmembrane domain alignments to Bacterial/Archaeal transporters (BAT), DMT, and MFS sequences supports SMR participation in multidrug transport evolution by identifying common TM domains.

**Conclusion:**

Based on this study, PSMR sequences originated recently within both SUG and SMP clades through gene duplication events and it appears that SMR members may be evolving towards specific metabolite transport.

## Background

Anthropogenic drug overuse combined with the rapid horizontal distribution of multidrug efflux genes encoded on mobile genetic elements has facilitated drug resistance in distant or unrelated microorganisms [1-3]. One such gene encode small multidrug resistance (SMR) proteins which are frequently identified within the 3' conserved region of mobile genetic elements referred to as integrons [4] and on various multidrug resistance plasmids [5-8]. SMR proteins are characterized by their short amino acid length (100–150 amino acids) resulting in a four transmembrane (TM) stranded α-helical protein that confers low-level resistance to a broad range of drugs using proton motive force (as reviewed by [9]). These drugs include a wide variety of antiseptics, namely quaternary ammonium compounds (QAC) and toxic lipophilic compounds, such as DNA interchelating dyes (as reviewed by [9]). In addition to QAC, members of the SMR protein family also demonstrate the ability to efflux other compounds such as potentially toxic metabolites like nicotine intermediates [10] and polyamine compounds like spermidine [11] implying that SMR proteins may play a broader role in toxic compound regulation.

The SMR protein family can be subdivided into three subclasses namely, small multidrug proteins (SMP), suppressor of *groEL *mutations (SUG), and paired small multidrug resistance (PSMR) subclasses (reviewed by [9,12]). The SMP subclass is characterized by its ability to confer host resistance to a broad range of lipophilic drugs and QAC. Members from the SMP subclass include small multidrug resistance (Smr) proteins from Archaea and Firmicutes, ethidium multidrug resistance protein E (EmrE) from Proteobacteria, and plasmid and/or integron encoded Qac proteins such as QacE, QacF and QacH (as reviewed by [9]). Proteins from this subclass are the most frequently studied members within the SMR family and *Escherichia coli *EmrE (Eco-EmrE) serve as the paradigm for all SMR members.

SUG subclass members were initially identified based on their ability to suppress *groEL *mutation phenotypes [13] and these proteins are speculated to support cellular chaperone activity (as reviewed by [9,12]). Members from this subclass confer host resistance to a limited subset of QAC compounds emphasizing their distinction from other SMR homologues [14,15]. To date, the SUG subclass consists primarily of SugE members identified from bacterial genomes but additional SUG homologues are also present on integrons and conjugative multidrug resistance plasmids [16,17] that include, *qacC' *[18] and *smr-2 *[19]. Members of the SUG subclass have been identified within a variety of Bacterial classes yet only two homologues are functionally characterized to date, specifically *E. coli *SugE (Eco-SugE) and *Citrobacter freundii *(Cfr-SugE) [12,14,15,20].

Members of the PSMR subclass are distinct from both SMP or SUG subclasses since they require co-expression of two SMR homologues to confer host resistance to QAC and toxic metabolites [10,11,21-25]. Generally, the genes encoding for PSMR protein pairs are located adjacently in a single operon at a separate genetic locus from other SMR subclass members within the host [26,27]. To date, the PSMR subclass includes the experimentally characterized pairs YdgE (MdtI)/YdgF (MdtJ) in Proteobacteria, and YkkC/YkkD, YvdR/YvdS, EbrA/EbrB, and YvaD/YvaE in Actinobacteria. However, not all PSMR members have demonstrated drug resistance, namely *B. subtilis *YvdR/YvdS and YvaD only of the YvaD/YvaE pair [26] which suggests that these SMR homologues are likely involved in the transport of as yet unidentified compounds or metabolites.

Each SMR subclass is thought to have a similar structural architecture based on hydropathy plot analysis and predicts that all SMR homologues adopt four TM strands connected by short loops of varying hydrophilicity [26,28]. One of the most highly conserved amino acid residues found within any of the TM strands is a negatively charged Glu residue (position 14 according to Eco-EmrE) in TM strand 1 (TM1). Biochemical studies of SMR subclass members from both Gram-positive and Gram-negative hosts have identified the importance of this residue for transport activity as part of the active site (as reviewed by [9]). Previous examination of SMR protein alignments have revealed that SMP and SUG subclasses possess unique conserved amino acid motifs that likely reflect these functional differences [9].

The short length and limited number of TM strands that comprise the SMR protein family have led to the proposal that they are the evolutionary building blocks of larger α-helical multidrug efflux proteins [29-31]. Although the sequence similarity between SMR protein members and other larger multidrug resistance transporters (composed of 5–10 TM strands) is poor, the structural arrangements of the TM strands often appear similar. Duplication of SMR genetic regions that encode for single TM strands or entire SMR genes have been speculated to give rise to members within the drug metabolite transport (DMT) superfamily such as the five TM stranded Bacterial/Archaeal transporter (BAT) family members and ten TM stranded transporters such as members of the drug metabolite exporter (DME) [30]. Experimental evidence supporting this evolutionary model is unavailable but artificially duplicated Eco-EmrE gene cassette fusions result in a functional protein displaying parallel topology between the fused subunits [32].

The objective of this work is to explore the evolutionary origins of the SMR protein family by examining SMR diversity among Archaeal and Bacterial genomes. The specific goal is to gain insight into the evolutionary constraints exerted upon SMR subclass members to validate the notion that SMR proteins acted or are acting as the evolutionary starting points for larger transporters. We began these studies by surveying SMR sequences within Archaea and Bacteria from diverse taxonomic backgrounds using current genomic and plasmid sequence databases (as of March 2008). This effort identified 685 SMR amino acid sequences which were aligned to putatively classify SMR sequences into the three SMR protein subclasses. The evolutionary relatedness of assigned SMR protein sequences was refined by using phylogenetic analysis and assisted with SMR homologue classification. The degree of amino acid conservation and the selective pressures exerted upon selected taxonomic SMR representatives was determined by their rate of synonymous to non-synonymous nucleotide substitutions. Finally, the genomic loci of SMR homologues were surveyed within the currently sequenced Archaeal and Bacterial genomes to explore their functional association to metabolite transport and their affiliation with transposon and integron mediated inheritance among diverse microorganisms. The results from this bioinformatics exploration support the current models that SMR protein sequences act as genetic building blocks for much larger multidrug efflux proteins based on their diversity and rapid evolution.

## Results and Discussion

### SMR proteins have unique subclass distributions within Archaeal and Bacterial classes

To understand and explore the extent of SMR subclass distribution and diversity, we surveyed the NCBI sequence database for chromosomally encoded SMR sequences among Archaea and Bacteria using characterised SMR homologues from B. *subtilis *and *E. coli *as seed sequences. In total, 685 putative SMR protein sequences were retrieved from Archaea and Bacteria and a summary of their subclass distribution is presented in Table 1.

**Table 1:** The distribution of SMR homologues into each of the three SMR protein family subclasses within Archaea and Bacteria.

	**SMR subclass members (%)**										
				
	**SMP**	**SUG**	**PSMR^c^**					**Qac^d^**			
				
**Archaeal and Bacterial Classes**			**A**	**B**	**C**	**D**	**E**		**Total number SMR/Bacteria**	**Total number species with SMR**	**Mean SMR/species**
**Euryarchaeota**	**30**	**40**					**30**		**10**	**10**	**1**
Halobacteria	60	40							5	5	1
Methanomicrobia		40					60		5	5	1
											

**Bacteria**	**28**	**32**	**11**	**7**	**8**	**3**	**6**	**4**	**675**	**330**	**2**
Actinobacteria	6	43		48				4	54	28	2
Bacilli		13		12	32	25	14	5	81	22	4
Lactobacilli		59		18	24				17	11	2
Bacteroidetes		100							4	4	1
Chlorobia	7	53					40		15	9	2
Flavobacteria	20	80							5	4	1
Clostridia		36		9	18		36		11	7	2
Cyanophyceae	43	14		19			24		21	15	1
Deinococci		63	25	13					6	3	2
Chlamydiales		100							2	2	1
Planctomycetacia		50		50					3	3	1
Chloroflexi	43			14			43		8	7	1
α-proteobacteria	48	45	4				2	1	100	63	2
β-proteobacteria	42	42	7				6	3	71	36	2
γ-proteobacteria	41	25	21		2		2	9	213	95	2
δ-proteobacteria	21	52	21				7		29	13	2
ε-proteobacteria		6	40		54				35	11	3
											

**Total number of sequenced organisms surveyed with SMR**										**340**	

**Total number of sequenced organisms surveyed**										**988**	

**Percentage of sequenced organisms with SMR (%)**										**34**	

The number of SMR sequences for each kingdom and class are indicated by percentage.

^a ^The number of SMR sequences for each kingdom and class are indicated as a percentage of the total number identified.

^b ^*yvaE *genes are only paired with *yvaD *genes among Bacilli and Lactobacilli.

^c ^PSMR pairs are indicated as follows corresponding to the indicated letter: *ydgE *and *ydgF*(**A**), *ebrA *and *ebrB *(**B**), *ykkC *and *ykkD *(**C**), *yvdR *and *yvdS *(**D**), and *yvaE *and *yvaD *(**E**)

^d ^Qac refers to integron and plasmid derived seqeunces only.

The results from Archaeal genome surveys identified that SMR sequences are present within Euryarchaeal sub-phylum genomes (Table 1). The lack of SMR homologue detection in the genomes of available Crenarchaeal sub-phylum species may reflect insufficient taxonomic diversity or the presence of highly divergent SMR sequences that prohibited identification. This latter observation is supported by the identification of only three putative SMR sequences from Crenarchaeal genome BLAST searches with e-values at the minimum significance threshold (5 × 10^-3^). All three Crenarchaeal SMR sequences (all annotated as putative QAC transporters within the database) served as outgroups for subsequent phylogenetic analysis and will not be discussed further.

Overall, SMR homologues we identified from surveyed Euryarchaeal genomes belonged to SUG, SMP, and the PSMR (only YvaE) subclasses only (Table 1). All Euryarchaeal SMR homologues were found in two classes, namely Methanomicrobia and Halobacteria. These surveyed genomes possessed a single SMR homologue, present as a single isogenic copy but had variable SMR subclass identity within similar genera in each Archaeal class. Although SUG and SMP members were commonly identified from this survey, Euryarchaeal Methanomicrobia were the only organisms to show homology to any PSMR subclass member, namely to *B. subtilis *YvaE (Bsu-YvaE). The presence of Methanomicrobia *yvaE *homologues could suggest that YvaE potentially represents a PSMR progenitor. This observation is supported upon closer examination of their genomic loci, where all of the Euryarchaeal *yvaE *homologues lacked the presence of *yvaD *a poorly conserved SMR that is commonly located in the same putative operon among *Bacillus *species [26].

As expected from the wealth of bacterial genomic sequences available, SMR diversity among bacterial species far outnumbered Archaea (Table 1). Surveys of the NCBI bacterial genomic database revealed SMR sequences were present in 34% of the genera we examined (as of March 2008). This total is lower than previously reported estimates (~52% by [9,26]) and will likely vary as more genomic information is collected. However, this value emphasizes the extent of SMR proliferation in the Bacterial kingdom. As shown in Table 1, Bacteria have two SMR homologues per bacterium based on the overall average but the determination of which SMR subclass member participates in this average is difficult to identify since SMR sequences distributed more or less equally within all three SMR subclasses. Examination of SMR subclass identity by each Bacterial class we surveyed revealed a far more specific SMR distribution than the overall bacterial SMR average indicated in Table 1.

The SUG subclass prevailed over all other subclasses in Bacteria since SUG homologues were found in nearly every class we examined (Table 1). The presence of SUG sequences from almost all Euryarchaeal and Bacterial classes we surveyed indicate that SUG homologues may represent a potentially ancient SMR sequence. However, this observation fails to explain the lack of SUG members from the Bacterial class Chloroflexi or the relatively low frequency of SUG identification within the genomes of ε-proteobacteria (6%), Bacilli (13%), and Cyanophyceae (14%) (Table 1). SUG identification within most bacterial classes indicates that SUG sequences are maintained under selective pressures that differ from other SMR homologues within their hosts.

**Figure 1:**
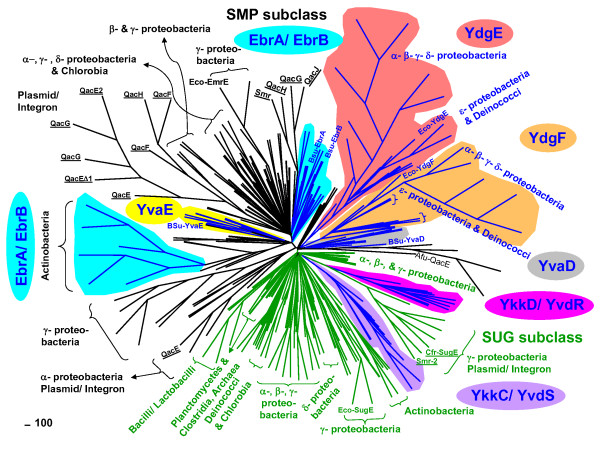
**Phylogenetic tree of SMR protein family members selected from Archaea and Bacteria**. The unrooted phylogenetic tree is based on a NJ analysis of 338 SMR protein sequences identified from various Archaeal and Bacterial species. Due to the large number of sequences and tight clustering of branches within this tree, genus and species names were omitted and taxa were described according to their bacterial class. NCBI accession numbers of all SMR protein sequences and their genus and species names are shown in Additional Files 1 and 3. Plasmid and integron encoded SMR proteins are underlined and *E. coli *(Eco-EmrE, Eco-SugE, and Eco-YdgE/Eco-YdgF) and *B. subtilis *(Bsu-EbrA/Bsu-EbrB, Bsu-YkkC/Bsu-YkkD, Bsu-YvdR/Bsu-YvdS, and Bsu-YvaE/Bsu-YvaD) SMR homologues are indicated at their respective branch. The Archaeal *Archaeoglobus *Afu-QacE sequence served as an outgroup for this analysis. Bootstrap values were calculated but not shown on this tree; refer to Additional File 1 for confidence values at the respective nodes. Branches are coloured corresponding to their SMR subclass designation, PSMR (blue), SUG (green), and SMP (black) and individual PSMR members are highlighted according the following colours; YvaE (yellow), YvaD (Grey), YkkC/YvdS (violet), YkkD/YvdR (pink), YdgE (red), YdgF (orange), EbrA/EbrB (light blue).

SMP subclass sequences were identified within the genomes of particular Bacterial classes namely, Cyanobacteria, Chloroflexi, as well as α-, β- and γ-proteobacteria (Table 1). Unlike SUG, many Bacterial classes either lacked SMP sequences altogether or encoded for SMP homologues at a lower frequency (Table 1). Bacterial species in possession of SMP homologues within these classes were either associated with nosocomial or strict pathogen causing microorganisms or found in either aquatic or soil dwelling environments. Hence, SMP maintenance within its host microorganism in these types of environments likely reflects their frequent exposure to QAC and other toxic compounds excreted from other biological or anthropogenic sources. Many Bacterial species, particularly those from Actinobacteria and Proteobacteria, had chromosomally encoded SMP homologues in addition to plasmid and strictly integron encoded Qac sequences such as QacE, QacEΔ1, QacG, QacH, QacF, and QacJ (Table 1). These Qac sequences demonstrated sufficient sequence similarity (QacE 80.9%, QacEΔ1 73.7%, QacF 80.0%, QacG 80.0%, QacH 81.0%, QacJ 81.0% to Eco-EmrE respectively) to their chromosomally encoded SMP protein sequences to be considered members of the SMP subclass. The distribution of chromosomally encoded SMP may be highly influenced by their close association to Qac encoded multidrug resistance plasmids and integrons (a trend also noted in our phylogenetic analysis and genomic loci surveys; Figure 1 and Table 2). However, SMP sequences are not strictly associated with host plasmid/integron presence in the host organism, since surveyed Chlorobia and δ-proteobacteria genera lacked Qac encoding plasmids/integron sequences. Therefore, lateral gene transfer is likely only one of many mechanisms at play in current Bacterial SMP diversity and supports observations made for other integron associated genes from the genera *Xanthomonas *[33] or *Vibrio *[34-36] that are chromosomally inherited and maintained within closely related species.

**Table 2:** SMR subclass member diversity within putative metabolite operons and their association to common metabolite ORF based on genomic loci surveys of sequenced Archaeal and Bacterial chromosomes.

			**SMR association to the following operons**								
			
				**Amino Acid Transport/Metabolism**				**Multidrug Resistance**		**Lipid Mtabolism**	
**SMR Subclass**	**Subclass Member**	**Total number of Loci**		**Lys/Arg**	**Trp/Tyr/Phe**	**Other Amino Acids**	**Polyamine (spe/cad/put) & Betaine**	**Glyco-peptide & Poly-ketide**	**β-lactam metabolism**	**Sn-G3P**	**Fatty Acid synthesis**

**SUG**	***sugE***										

Putative operons		118		lys	trp/aro	liv/ilv/pro	put/spe/bet	MFS/ABC/ecn/bleo	tetR/pbp	ugp/pls	fab/acc
Frequency in operon/total surveyed loci				0.8%	4.2%	1.7%/0.8%/0.8%	0.8%/1.7%/0.8%	3.4%/2.5%/3.4%/0.8%	5.1%/0.8%	0.8%/2.5%	1.7%/0.8%
Frequency of occurrence in each locus (10 gene radius)				29.7%	20.3%	2.0%	5.1%	22.9%	23.7%	7.6%	17.8%

**SMP**	***emrE/smr***										

Putative operons		92		lys	tyr	pro/his/met/cys	put/bet/dpp	ABC/bleo/hlx	tetR/pbp	glp	fab/ech
Frequency in operon/total surveyed loci				1.1%	1.1%	1.1%/1.1%/1.1%/2.2%	1.1%/3.3%/1.1%	1.1%/1.1%/2.2%	8.7%/1.1%	3.3%	1.1%/2.2%
Frequency of occurrence in each locus (10 gene radius)				22.8%	14.1%	12.0%	14.1%	28.3%	10.9%	9.8%	22.8%

**PSMR**	yvaE**										

Putative operons		16		---	---	pro	---	MFS/ABC	tetR	---	acp/fab
Frequency in operon/total surveyed loci				---	---	12.5%	---	6.3%/18.8%	1.1%	---	12.5%/6.3%
Frequency of occurrence in each locus (10 gene radius)				25.0%		18.8%	---	43.8%	12.5%	6.3%	18.8%

**PSMR**	yvaD**										

Putative operons		7		arg	---	---	---	---	tetR	---	acp/fab
Frequency in operon/total surveyed loci				14.3%	---	---	---	---	14.3%	---	28.6%
Frequency of occurrence in each locus (10 gene radius)				14.3%	---	---	---	---	28.6%	---	42.9%

**PSMR**	***ydgE/ydgF***										

Putative operons		22		lysR	---	---	spe*	---	---	---	---
Frequency in operon/total surveyed loci				4.5%	---	---	---	---	---	---	---
Frequency of occurrence in each locus (10 gene radius)				54.5%	---	---	31.8%	22.7%	---	---	22.7%

**PSMR**	***ebrA/ebrB***										

Putative operons		16		---	---	---	---	---	---	---	---
Frequency in operon/total surveyed loci				---	---	---	---	---	---	---	---
Frequency of occurrence in each locus (10 gene radius)				12.5%	6.3%	---	6.3%	31.3%	43.8%	---	50.0%

**PSMR**	***ykkC/ykkD***										

Putative operons		9		---	aro	liv	spe	MFS	---	---	---
Frequency in operon/total surveyed loci				---	11.1%	11.1%	11.1%	11.1%	---	---	---
Frequency of occurrence in each locus (10 gene radius)				22.2%	55.6%	11.1%	11.1%	33.3%	33.3%	11.1%	44.4%

**PSMR**	***yvdR/yvdS***	3		NA	NA	NA	NA	NA	NA	NA	NA

**Total loci**		**283**									

			**SMR association to the following operons**								

			**Vitamin Metabolism**				**Nucleotide Metabolism**		**Horizontal Gene Transfer System**		

**SMR Subclass**	**Subclass Member**	**Total number of Loci**	**Vitamin (Vit.) B1, B2, & BB3 (thi, rib, nia)**	**Vit. B7 & BB9 (bio/fol)**	**Vit. B6 & BB12 (Pyd & Cob)**	**Coenz. Q10 (Ubi) & F420**	**Pur**	**Pyr**	**Int/Tn/Mat**	**Plasmid**	**Phage genes**

**SUG**	***sugE***										

Putative operons		118	thi/nad	fol	---	ubi	pur	ctp	tn/rve	vap/kill	---
Frequency in operon/total surveyed loci			0.8/2.5%	0.8%	---	1.7%	4.2%	0.8%	2.5%	2.5%	---
Frequency of occurrence in each locus (10 gene radius)			7.6%	1.7%	2.5%	11.9%	22.0%	3.4%	21.2%	9.3%	5.9%

**SMP**	***emrE/smr***										

Putative operons		92	thi/nad	bio/fol	cob	---	pur	---	tn/rve	---	Pro-phage DLP12
Frequency in operon/total surveyed loci			1.1%/1.1%	1.1%/1.1%	2.2%	---	1.1%	---	3.3%	---	1.1%
Frequency of occurrence in each locus (10 gene radius)			17.4%	12.0%	3.3%	9.8%	8.7%	3.3%	14.1%	2.2%	4.3%

**PSMR**	yvaE**										

Putative operons		16	---	---	---	---	---	---	---	---	---
Frequency in operon/total surveyed loci			---	---	---	---	---	---	---	---	---
Frequency of occurrence in each locus (10 gene radius)			---	6.3%	---	25.0%	---	---	12.5%	---	---

**PSMR**	yvaD**										

Putative operons		7	---	---	---	ubi	pur	---	---	---	---
Frequency in operon/total surveyed loci			---	---	---	28.6%	14.3%	---	---	---	---
Frequency of occurrence in each locus (10 gene radius)			---	14.3%	---	42.9%	42.9%	28.6%	14.3%	---	---

**PSMR**	***ydgE/ydgF***										

Putative operons		22	---	---	---	---	---	---	tn/rve	tra/cop	---
Frequency in operon/total surveyed loci			---	---	---	---	---	---	4.5%	9.1%	---
Frequency of occurrence in each locus (10 gene radius)			13.6%	18.2%	---	---	18.2%	4.5%	9.1%	9.1%	13.6%

**PSMR**	***ebrA/ebrB***										

Putative operons		16	nad*	fol	---	---	pur	---	---	---	---
Frequency in operon/total surveyed loci			12.5%	6.3%	---	---	6.3%	---	---	---	---
Frequency of occurrence in each locus (10 gene radius)			18.8%	25.0%	12.5%	6.3%	25.0%	---	25.0%	---	6.3%

**PSMR**	***ykkC/ykkD***										

Putative operons		9	rib	bio	---	---	pur	pyr	---	cdt	---
Frequency in operon/total surveyed loci			11.1%	11.1%	---	---	22.2%	11.1%	---	11.1%	---
Frequency of occurrence in each locus (10 gene radius)			22.2%	11.1%	11.1%	---	33.3%	11.1%	11.1%	11.1%	---

**PSMR**	***yvdR/yvdS***	3	N/A	N/A	N/A	N/A	N/A	N/A	N/A	N/A	N/A

**Total loci**		**283**									

* Indicates these SMR members have experimentally demonstrated transport involvement in the metabolite transport.

** Calculated value listed in table includes both isogenic gene occurances and gene pairs.

**Abreviations of genes listed in table: ABC **ABC-type antimicrobial peptide transport system; **aro **aromatic amino acid biosynthesis; **bet **transport and biosynthesis/degradation of glycine betaines; **bleo **bleomycin resistance genes; **bio **involved in biotin (vitamin B7) metabolism; **cdt **plasmid encoded cytotoxin genes; **cob **involved in cobalamin vitamin B6 metabolism; **dpp **ABC-type dipeptide/oligopeptide/nickel transport system; **fol **involved in folate metabolism; **glp **utilization of glycerol and sn-glycerol 3-phosphate (sn-**G3P**); **hlx **hemolysin genes involved in host virulence; **lysR **lysine transcriptional regulator; **liv/ile **branched amino acid biosynthesis (val, ile, leu); **met/pro/his/cys **amino acid biosynthetic genes; **MFS **multidrug efflux major facillitator superfamily genes; **nad **involved in nicotine and nicotinamide metabolism; **nag **GlcNAc uptake and metabolism; **nai **(niacin) vitamin B3 metabolism; **pbp **penicillin binding proteins/cell wall biosynthesis proteins; **pls **involved in sn-glycerol-3-phosphate phsopholipid biosynthesis; **put **transport and biosynthesis/degradation of putrescine; **pts **phosphoenolpyruvate-dependent phosphotransferase system; **pur **involved in purine nucleotide biosynthesis; **pyd **(pyridoxine) vitamin B12 metabolism **pyr **involved in pyrimidine nucleotide biosynthesis; **rib **involved in riboflavin metabolism; **spe **transport and biosynthesis/degradation of spermidine; **tetR **tetracyclin resistance transcriptional regulator; **thi **(thiamin) vitamin B1 metabolism; **tn/rve **transposons and integron maturases; **trp/tyr **biosynthesis of tryptophan/tyrosine; **ubi **involved in ubiquinone (coenzyme Q10) biosynthesis; **ugp **uptake of sn-glycerol-3-phosphate and glycerophosphoryl diesters; **vap/kill **host plasmid virulence and toxin genes.

PSMR subclass members have the most specific sequence distribution among the sequenced Bacterial genomes we surveyed strongly suggesting they are maintained under selective pressures different from either the SUG or SMP subclasses (Table 1). Particular PSMR pairs themselves are uniquely distributed within each Bacterial class we examined. For specific examples, refer to ε-proteobacterial YdgE and YdgF pairs and YkkC and YkkD compared to γ- and δ-proteobacteria YdgE and YdgF only in Table 1). The PSMR distribution among Bacilli demonstrated far greater diversity where EbrA/EbrB, YkkC/YkkD, YvdR/YvdS, and YvaE/YvaD members were identified (Table 1). Hence, PSMR distribution appears to be highly selective, as observed by the complete lack of identified YdgE/YdgF homologues from almost all Gram-positive organisms we surveyed (except in Deinococci) (Table 1). In contrast, only the PSMR homologue YvaE was found throughout Gram-positive and Gram-negative genomes we surveyed, albeit at a lower frequency than other SMR subclass members. The presence of *yvaD *in Bacilli and Lactobacilli only suggests that *yvaD *may be a rapidly diverging *yvaE *gene duplication product within Bacilli and Lactobacilli.

Taken together, the unique distribution pattern of SUG, SMP and PSMR subclass members strongly supports SMR diversification that is tailored to particular genera.

### The phylogenetic history of SMR proteins

To explore the evolutionary relationships of SMR protein family members further, we analyzed a taxonomically diverse set of 338 SMR protein sequences. Previous phylogenetic analyses of SMR amino acid sequences using smaller taxonomic data sets [9,12,26,37] demonstrated that SUG, SMP and PSMR subclasses grouped separately from each other. To determine the phylogenetic relatedness of SMR subclass members within various Archaeal and Bacterial classes to plasmid/integron encoded homologues, we broadened the taxonomic sampling range. If horizontal gene transfer is the dominant influence on SMR distribution, we expect a highly discordant phylogenetic distribution closely related to plasmid and integron Qac sequences rather than a linear inheritance.

Our phylogenetic analysis demonstrated a division of SMR members into two major clades (Figure 1; Additional File 1), namely SUG and SMP, similar to earlier phylogenetic analyses [9,12,26]. Unfortunately, our phylogram shows that both SUG and SMP clades reside in an unresolved polytomy, a common occurrence in dendrograms of sequences dispersed via lateral gene transfer mechanisms (for examples refer to [3,38]). This prevents reliable determination of a common ancestor for either the SUG or SMP subclasses from this analysis. However, both SUG and SMP clades demonstrate branching patterns that follow some host driven inheritance, where SMR members of the same bacterial class group together. However, SUG and SMP sequences originating from diverse Bacterial classes were observed suggesting a common lateral transfer of SMR sequence within these taxonomic groups (Figure 1; Additional File 1).

Since the greatest number of SMR homologues we identified are from Actinobacteria, Bacilli, and various Proteobacteria, it is not surprising that the phylogenetic groupings of SMR homologues within our dendrogram are strongly dominated by these Bacterial classes (Figure 1; Additional File 1). Previous studies of Class I integron mobility within all of these genomes indicated that Actinobacteria, and Proteobacteria bacterial classes in particular, are highly favourable targets for horizontal gene traffic and may explain the abundance of SMR homologues within these particular classes [39,40]. Furthermore, there is an inherent codon bias within Class 1 integrons (which typically encode for SMP homologues at the conserved 3' end) that may have been particularly optimized for these Bacterial classes [40]. Based on the diversity of Bacterial genera represented in the branches within both the SUG and SMP clades, it is likely that a combination of heritable and horizontal SMR gene transfer events have taken place. However, SMR dissemination by means of horizontal gene transfer appears to predominate simply based on the frequency of unrelated taxa grouping within the branches of both SUG and SMP clades.

Similar to the observations made from SMR sequence surveys (Table 1), a trend between the environmental origins of SMR homologues to their host can be observed. SUG or SMP homologues that grouped together with apparent mixed taxonomic origins often shared a common environmental niche or lifestyle (aerobic/anaerobic) (Figure 1; Additional File 1). This phylogenetic arrangement supports previous studies that demonstrated class 1 integron dissemination patterns among evolutionarily unrelated bacteria are strongly influenced by the selective pressures imposed by a shared ecological environment [40-42]. Therefore, SUG and SMP inheritance within Archaea and Bacteria is also influenced by environmental pressures or organism lifestyle and the impact of each influence appears to be unique within SUG and SMP clades.

PSMR subclass homologues grouped in distinct branches to either the SUG or SMP clades (Figure 1; Additional File 1). YvaD homologues were the only exceptions; since they grouped outside of either SUG or SMP clades close to the Crenarchaeal SMR outgroup sequences. PSMR members closely related to SUG members were YkkC/YvdS and YkkD/YvdR and formed distinct groupings adjacent to proteobacterial and plasmid/integron encoded SUG homologues within the SUG clade. This indicates that YkkC/YvdS and YkkD/YvdR homologues originated from the SUG subclass since both branches group to a common node within the SUG clade with high levels of confidence. Moreover, the close association of YkkC/YvdS and YkkD/YvdR in separate branches within this PSMR grouping strongly suggest that YvdS and YvdR homologues are closely related to YkkC or YkkD. The presence of both YkkC/YkkD and YvdR/YvdS sequences at separate loci within the genomes of Bacilli and Lactobacilli indicate that both PSMR pairs likely derived from separate SUG gene duplication events. Furthermore, YkkD/YvdR homologues show a close association to plasmid and integron encoded SUG homologues indicating that these PSMR homologues may have originated via horizontal gene transfer and explain their presence in unrelated taxa (Figure 1; Additional File 1). Similar to the SUG clade, PSMR members EbrA and EbrB, YdgE and YdgF, and YvaE formed distinct branches within the SMP clade with relatively high levels of confidence (Figure 1; Additional File 1). Like PSMR members related to the SUG clade, PSMR branches within the SMP clade were composed of taxa originating from diverse Bacterial classes in addition to sequences for a particular class (Figure 1; Additional File 1). The polychotomous branching within SMP prohibits reliable identification of an ancient SMP ancestor from the tree but does strongly support that SMP sequences serve as the origin of these particular PSMR sequences based on higher bootstrap values at their nodes. Therefore, PSMR subclass members with the exception of YvaE have emerged recently within the clades of both SUG and SMP likely due to gene duplication events of either a SUG or SMP homologue within particular Bacterial classes. The occurrence of PSMR sequences within certain Bacterial classes may reflect the different selection pressures placed on either SUG or SMP homologues to rapidly diversify for a particular environment or host physiology.

### Syn-SCAN analysis indicates each SMR subclass member is maintained under different selective pressure

Since SMR subclass distribution is enriched within particular Bacteria, we wanted to compare the frequency of synonymous to non-synonymous changes occurring within SMR sequences at the nucleotide level. Examining the degree of nucleotide changes throughout the whole SMR sequence relative to the rate of change at each codon can identify regions of the protein that are actively evolving. Experimentally examined SMR sequences, specifically SMR homologues from *E. coli *and *B. subtilis*, were selected for pairwise comparison to their respective putative homologues from Bacterial and Euryarchaeal class in our survey. Integron and plasmid encoded SMR homologues were included in this analysis to determine the extent of nucleotide changes within chromosomally encoded SMR sequences compared to the rate of divergence from their horizontally transferred counterparts. This comparison also served as a method to gauge differences that arise from the codon bias within the various host genomes.

The rate of synonymous to non-synonymous changes (dS/dN) in nucleotide sequences of SMP subclass homologues selected from various Euryarchaeal and Bacterial genera resulted in a mean value of 3.6 (Table 3). Since this value exceeds 1.0, it indicates that SMP homologues are maintained under selective pressure within the examined microorganisms. This value is very close to the mean dS/dN value of the SUG subclass (3.4) and suggesting that both SMR subclasses are under similar levels of selective pressures despite their known functional differences (Table 3). However, mean dS/dN values for members of the PSMR subclass are much lower (1.8–2.0), indicating that the selective pressures exerted on these sequences are less stringent and reflect ongoing sequence divergence from either SUG and SMP subclasses similar to observations made from our phylogram (Additional file 1; Figure 1).

**Table 3:** Summary of synonymous to non-synonymous nucleotide substitution patterns within SMR family subclasses SMP and SUG.

**SMR 1**	**SMR 2**	**Sd**	**Nd**	**S**	**N**	**pS**	**pN**	**dS**	**dN**	**dS/dN**
**SMP**	**mean**	57.27	83.94	79.63	220.37	0.72	0.38	1.68	0.55	**3.57**

Hsa-*smr*	Tfu-*smr*	39.12	88.88	85.17	214.83	0.46	0.41	0.71	0.6	1.18
	Ser-*smr*	43.12	90.88	85.5	214.5	0.5	0.42	0.84	0.62	1.34
	Cli-*smr*	55	86	82.33	217.67	0.67	0.4	1.66	0.56	2.96
	Fjo-*smr*	71.88	90.12	77.67	222.33	0.93	0.41	NA	0.58	NA
	Ssp-*emrE*	50.25	88.75	84.83	215.17	0.59	0.41	1.17	0.6	1.95
	Cau-*emrE*	56	85	82.83	217.17	0.68	0.39	1.74	0.55	3.14
	Bja-*emrE*	44.88	86.12	83.5	216.5	0.54	0.4	0.95	0.57	1.67
	Bme-*emrE*	55.38	70.62	83.67	216.33	0.66	0.33	1.61	0.43	3.75
	Bxe-*emrE*	49.88	77.12	85.5	214.5	0.58	0.36	1.13	0.49	2.30
	Pae-*emrE*	46.88	77.12	85.33	214.67	0.55	0.36	0.99	0.49	2.02
	Eco-*emrE*	66.5	96.5	79.33	220.67	0.84	0.44	NA	0.66	NA
	Gsu-*emrE*	49.88	78.12	84.5	215.5	0.59	0.36	1.16	0.5	2.34
	I-*qacE*	65.62	77.38	81.17	218.83	0.81	0.35	NA	0.48	NA
	PI-*qacEΔ1*	64.12	85.88	81.67	218.33	0.79	0.39	NA	0.56	NA
	P-*qacF*	47.75	79.25	80.83	219.17	0.59	0.36	1.16	0.49	2.35
	P-*qacG*	61	89	80.67	219.33	0.76	0.41	NA	0.58	NA
	P-*qacH*	71.25	102.75	76.5	223.5	0.93	0.46	NA	0.71	NA
	P-*qacJ*	72.88	92.12	78	222	0.93	0.41	NA	0.6	NA

Eco-*emrE*	Tfu-*smr*	70.75	98.25	79.5	220.5	0.89	0.45	NA	0.68	NA
	Ser-*smr*	67.62	105.38	79.83	220.17	0.85	0.48	NA	0.76	NA
	Cli-*smr*	57.5	79.5	76.67	223.33	0.75	0.36	NA	0.48	NA
	Fjo-*smr*	52.88	86.12	72	228	0.73	0.38	2.9	0.53	5.53
	Ssp-*emrE*	61	104	79.17	220.83	0.77	0.47	NA	0.74	NA
	Cau-*emrE*	55.25	85.75	77.17	222.83	0.72	0.38	2.32	0.54	4.30
	Bja-*emrE*	63.62	82.38	77.83	222.17	0.82	0.37	NA	0.51	NA
	Bme-*emrE*	59.12	75.88	78	222	0.76	0.34	NA	0.46	NA
	Bxe-*emrE*	63	74	79.83	220.17	0.79	0.34	NA	0.45	NA
	Pae-*emrE*	64.38	85.62	79.67	220.33	0.81	0.39	NA	0.55	NA
	Gsu-*emrE*	62.25	69.75	78.83	221.17	0.79	0.32	NA	0.41	NA
	I-*qacE*	47.5	84.5	75.5	224.5	0.63	0.38	1.37	0.52	2.62
	PI-*qacEΔ1*	47.5	88.5	76	224	0.62	0.4	1.34	0.56	2.39
	P-*qacF*	63.75	73.25	75.17	224.83	0.85	0.33	NA	0.43	NA
	P-*qacG*	60.12	83.88	75	225	0.8	0.37	NA	0.52	NA
	P-*qacH*	52.62	97.38	70.83	229.17	0.74	0.42	3.5	0.63	5.58
	P-*qacJ*	53.38	88.62	72.33	227.67	0.74	0.39	3.1	0.55	5.64

**SUG**	**mean**	55.11	91.77	78.26	221.74	0.71	0.41	1.83	0.63	**3.41**

Hla-*sugE*	Mba-*sugE*	60.5	73.5	75.83	224.17	0.8	0.33	NA	0.43	NA
	Tfu-*sugE*	45.25	75.75	80.83	219.17	0.56	0.35	1.03	0.46	2.22
	Ser-*sugE*	39.5	87.5	80.83	219.17	0.49	0.4	0.79	0.57	1.39
	Cli-*sugE*	61.25	76.75	78.83	221.17	0.78	0.35	NA	0.47	NA
	Fjo-SugE	64.75	82.25	75.17	224.83	0.86	0.37	NA	0.5	NA
	Gvo-*sugE*	50.38	66.62	80.83	219.17	0.62	0.3	1.33	0.39	3.42
	Bja-*sugE*	47.25	74.75	80	220	0.59	0.34	1.16	0.45	2.57
	Bme-*sugE*	55.38	79.62	78.5	221.5	0.71	0.36	2.12	0.49	4.33
	Bxe-*sugE*	50.25	72.75	80	220	0.63	0.33	1.36	0.44	3.13
	Pae-*sugE1*	49.5	82.5	80.83	219.17	0.61	0.38	1.27	0.52	2.43
	Pae-*sugE2*	47.12	57.88	82	218	0.57	0.27	1.09	0.33	3.33
	Eco-*sugE*	62	66	79.83	220.17	0.78	0.3	NA	0.38	NA
	Gsu-*sugE*	51.88	67.12	81.17	218.83	0.64	0.31	1.43	0.39	3.63
	I-*qacE*	62.38	109.62	78.5	221.5	0.79	0.49	NA	0.81	NA
	PI-*qacEΔ1*	60.12	116.88	79	221	0.76	0.53	NA	0.92	NA
	P-*qacF*	48.88	115.12	78.17	221.83	0.63	0.52	1.35	0.88	1.52
	P-*qacG*	51.75	116.25	78	222	0.66	0.52	1.62	0.9	1.80
	P-*qacH*	70.12	110.88	73.83	226.17	0.95	0.49	NA	0.8	NA
	P-*qacJ*	68.75	111.25	75.33	224.67	0.91	0.5	NA	0.81	NA
	P-Cfr-*sugE*	62.62	68.38	80.17	219.83	0.78	0.31	NA	0.4	NA
	I-*sugE*	56	89	79.83	220.17	0.7	0.4	2.05	0.58	3.54

Eco-*sugE*	Mba-*sugE*	61.62	83.38	76	224	0.81	0.37	NA	0.51	NA
	Tfu-*sugE*	60.38	76.62	81	219	0.75	0.35	3.82	0.47	8.10
	Ser-*sugE*	53.12	78.88	81	219	0.66	0.36	1.56	0.49	3.17
	Cli-*sugE*	57.25	65.75	79	221	0.72	0.3	2.54	0.38	6.71
	Fjo-*sugE*	57.25	91.75	75.33	224.67	0.76	0.41	NA	0.59	NA
	Gvo-*sugE*	60.75	59.25	81	219	0.75	0.27	NA	0.34	NA
	Bsp-*sugE*	55	68	80.17	219.83	0.69	0.31	1.85	0.4	4.63
	Bme-*sugE*	59.12	60.88	78.67	221.33	0.75	0.28	NA	0.34	NA
	Bxe-*sugE*	56.62	56.38	80.17	219.83	0.71	0.26	2.13	0.31	6.80
	Pae-*sugE1*	57.75	69.25	81	219	0.71	0.32	2.26	0.41	5.49
	Pae-*sugE2*	59.88	46.12	82.17	217.83	0.73	0.21	2.67	0.25	10.74
	Gsu-*sugE*	62.75	52.25	81.33	218.67	0.77	0.24	NA	0.29	NA
	I-*qacE*	57	102	78.67	221.33	0.72	0.46	2.54	0.71	3.55
	PI-*qacEΔ1*	54.5	109.5	79.17	220.83	0.69	0.5	1.87	0.81	2.31
	P-*qacF*	56.38	113.62	78.33	221.67	0.72	0.51	2.41	0.86	2.79
	P-*qacG*	60.5	99.5	78.17	221.83	0.77	0.45	NA	0.68	NA
	P-*qacH*	54.5	120.5	74	226	0.74	0.53	3.01	0.93	3.24
	P-*qacJ*	53.25	118.75	75.5	224.5	0.71	0.53	2.12	0.92	2.31
	P-Cfr-*sugE*	47.38	26.62	80.33	219.67	0.59	0.12	1.16	0.13	8.75
	I-*sugE*	59	98	80	220	0.74	0.45	3.07	0.68	4.54

**List of abreviations: Sd **is the number of observed synonymous substitutions; **Nd **is the number of observed non-synonymous substitutions; **S **is the number of potential synonymous substitutions; **N **is the number of potential non-synonymous substitutions; **pS **is the proportion of observed synonymous substitutions; **pN **is the proportion of observed non-synonymous substitutions; **dS **is the Jukes-Cantor correction for multiple hits of pS; **dN **is the Jukes-Cantor correction for multiple hits of pN.

**Species are listed according to a three letter species abbreviation followed by the SMR subclass name: Bja ***Bradyrhizobium japonicum*; **Bme ***Brucella melitensis*; **Bsp ***Bradyrhizobium sp. ORS278*; **Bxe ***Burkholderia xenovorans*; **Cau ***Chloroflexus aurantiacus*; **Cfr ***Citrobacter freundii*; **Cli ***Chlorobium limicola*; **Fjo ***Flavobacterium johnsoniae*; **Gsu ***Geobacter sulfurreducens*; **Gvo **Gloeobacter violaceus; **Hla ***Halorubrum lacusprofundi*; **Hsa ***Halobacterium salinarum*; **Mba ***Methanosarcina barkeri*; **Pae ***Pseudomonas aeruginosa*; **Ser ***Saccharopolyspora erythraea*; **Ssp ***Synechococcus sp. RS9917*; **Tfu ***Thermobifida fusca*; **P **plasmid encoded; **I **integron encoded; **PI **plasmid/integron encoded; **NA **data not available.

Individual dS/dN values from pairwise SMP sequence comparisons indicated that the rate of synonymous and non-synonymous changes range from 1–6 among examined chromosomal SMP sequences (Table 3). These values are often lower than mean dS/dN SMP values due to differences in codon usage between Archaea, Gram-positive, and Gram-negative organisms. However, the values were still above 1.0 indicating similar selective pressure acts on SMP nucleotide sequences even across a broad taxonomic sampling range. The dS/dN values for chromosomally encoded SMP sequences increased when compared to plasmid and integron encoded counterparts (2.3–5.6) demonstrating their close relationship. Often, dS/dN values could not be determined as indicated by NA in the table. This was due largely to the proportion of observed synonymous substitutions (pS) exceeding the Jukes-Cantor cut-off value of 0.74 preventing dS values from being accurately determined. However, looking at the pS values among many of the pairwise SMP comparisons it is apparent that many of the SMP sequences are maintained under high selective pressure similar to other sequences (Table 3).

SUG homologues are under similar levels of selective pressures as SMP when comparing their dS/dN values (Table 3). SUG dS/dN values were high when examining the Archaeal *Halorubrum lacusprofundi sugE *(Hla-*sugE*) sequence relative to the values of Eco-*sugE *(Table 3). Based on these dS/dN values, selective constraints that maintain SugE homologues like those from Halobacteria appear to be higher in extremophilic microorganisms perhaps suggesting potential niches of ecological favoring SUG enrichment. The selective constraint that acts upon pairwise comparisons of Eco-*sugE *sequences to plasmid/integron encoded *sugE *sequences increased dramatically with dS/dN values ranging from 2.3–10.4 supporting a close relationship and origin. Among chromosomally encoded SUG pairwise comparisons, the highest increase in dS/dN ratios are observed primarily for the α-proteobacterial and γ-proteobacterial sequences we examined otherwise, all other pairwise SUG comparisons reflected the mean SUG dS/dN ratio value. The increase in selective constraint on the α-proteobacterial and γ-proteobacterial SUG sequences agree with studies that show a frequent association of multidrug resistance gene cassettes with integrons, particularly those among Proteobacteria [42-44]. Since SUG sequences are maintained under moderate to high selective pressures as reflected by their dS/dN values and their predominance over other SMR subclass members (Table 1), it emphasizes their potentially distinct functional differences.

Members of the PSMR subclass appear to have very different selective constraints placed upon their sequences as reflected by the variable mean dS/dN values for each PSMR homologue in comparison to either SMP or SUG subclasses (Table 3 and Additional file 2). *B. subtilis *PSMR subclass sequences were selected as the comparison set for Syn-SCAN analysis since they represent the most functionally and structurally characterized members of this subclass to date [21,22,25,26]. Bsu-*yvaE *had the highest mean dS/dN value (3.16) compared to other PSMR members, reflecting its frequent diverse distribution in the genomes of both Archaea and Bacteria. Although the rate of synonymous to non-synonymous changes among comparisons of Bsu-*yvaE *to other *yvaE *homologues are in many cases indeterminable due to pS values exceeding the Jukes-Cantor threshold limits, those that could be calculated resulted in high overall dS/dN values (Additional file 2). Looking at pS and pN values alone, a similar high dS/dN ratio would be expected among many of the *yvaE *homologue comparisons that are listed as NA within Table 3. As expected from phylogenetic analysis and protein alignments, Bsu-*yvaD *had the lowest mean dS/dN ratio (1.63) suggesting that it is maintained under the least amount of selective pressure among all the PSMR members we examined.

PSMR homologues *ebrA*/*ebrB *have a slightly lower mean dS/dN value (2.84) than the mean dS/dN values of *yvaE*, SMP, and SUG homologues (Table 3 and Additional file 2). Comparisons of Bsu-*ebrA *and Bsu-*ebrB *together or with other chromosomal *ebrA*/*ebrB *homologues demonstrated that both sequences are maintained under slightly lower selective pressures (dS/dN values of 1.0–4.1), but not when compared to integron encoded Qac homologues (dS/dN values of 4.5–5.6) (Additional file 2). Similar to our phylogram (Figure 1; Additional File 1), differences could be observed in dS/dN values when comparing *ebr*A/*ebr*B homologues identified from Bacilli and Proteobacterial hosts relative to the Actinobacterial *ebrA*/*ebrB *homologues (Additional file 2) which branched separately in the dendrogram. This finding supports the observation that Actinobacterial *ebrA/ebrB *homologues in particular are under different selective pressures from other *ebrA/B *homologues that may be altering these SMP clade members towards a particular function within Actinobacterial hosts. EbrA/EbrB homologues NepA and NepB encoded on the *Arthrobacter nicotinovorans *pAO1 plasmid confer host resistance to nicotinamide and its intermediates suggesting that these particular PSMR members may play specific roles in metabolite toxin excretion [10].

YdgE/YdgF homologues demonstrated a much lower mean dS/dN value (1.84) suggesting that these homologues are under far less selective pressure than the SMP or SUG members surveyed (Additional file 2). However, looking at *ydgE *and *ydgF *homologues separately shows a different reason for their relatively low mean dS/dN value as a pair. *ydgE *homologues had higher dS/dN values (0.6–2.6) than its counterpart *ydgF *(0.3–0.6) indicating that only *ydgE *is actively maintained. This trend is in good agreement with observations made by three dimensional cryo-electron microscopic structural studies of Eco-EmrE protein that support an asymmetrical arrangement of each protein monomer [45,46]. The lack of stringent selection on one of the two proteins within the YdgE/YdgF pair may enhance its functional and structural asymmetry. Experimental characterization of the paired YdgE/YdgF protein complex in *E. coli *has revealed its involvement in spermidine excretion and confirmed that only YdgE (MdtI) was essential for transport activity based on site-directed mutagenesis experiments of each active site Glu14 residue in the pair [11]. Furthermore, dS/dN comparisons of *ydgF *and *ydgE *homologues to plasmid and integron encoded SMR homologues indicated that only *ydgE *members were maintained at similarly high levels.

Finally, examination of mean dS/dN values for PSMR pairs, *ykkC*/*ykkD *(1.7) and *yvdR*/*yvdS *(2.0), revealed that each pair is maintained under moderately high selective pressures in comparison to all other PSMR members (Additional file 2). Unlike *ydgE*/*ydgF *homologues, pairwise dS/dN comparisons between *ykkC*/*ykkD *and *yvdR*/*yvdS *homolog pairs showed relatively high (> 1.0) selective pressures on both pairs. Active maintenance of both homologues in the pair may reflect functional interdependency. More importantly, only *ykkC *demonstrated high dS/dN values when compared to plasmid/integron encoded SUG homologues suggesting that *ykkC *has closer association to horizontally transferred SUG counterparts. This contrasts our phylogenetic analysis of these sequences which suggest YkkD has greater evolutionary relationships to plasmid and integron encoded SUG homologues (Figure 1, Additional File 1). The closely related *yvdR*/*yvdS *pair does not share dS/dN value differences observed between *ykkC *and *ykkD *when comparing either homologue to plasmid/integron encoded SUG homologues suggesting that both *yvdR *and *yvdS *are maintained under similar levels of selective pressures (Additional file 2). However, both PSMR pairs share far greater similarity to SUG homologues reinforcing their evolutionary relationship within the SUG clade.

So why is PSMR diversity within particular Bacterial classes so selective? Thus far, only the EbrA/EbrB homologues, NepA and NepB, from *Arthrobacter nicotinovorans *are plasmid encoded [10]. To elaborate, Bacilli lack isogenic SMP or SUG subclass homologues, which would suggest that there may be as yet unidentified integrons/plasmids harbouring these SMR homologues. Alternatively, current PSMR homologues may have derived from existing SUG and SMP subclass members through recent gene duplication and sequence divergence. The latter explanation may explain why two PSMR homologues are found almost exclusively within the chromosomes in contrast to their isogenic integron and plasmid encoded SMR counterparts. Laterally transferred SMR genes from either SUG or SMP subclasses should have more selective pressures acting to maintain their isogenically conferred resistance and this trend is reflected in our analyses of both subclass sequences (Table 3 and Additional file 2). Essentially, those isogenic SMP and SUG sequences that have incorporated into the genome are no longer subjected to the same selective pressures and can diverge functionally and structurally by duplication. Plasmid and integron encoded homologues must endure selective pressures that likely force them to be compact in size to thrive on mobile elements.

### SMR subclass members have unique amino acid conservation patterns highlighting different selective pressures acting upon loop and TM regions

**Figure 2:**
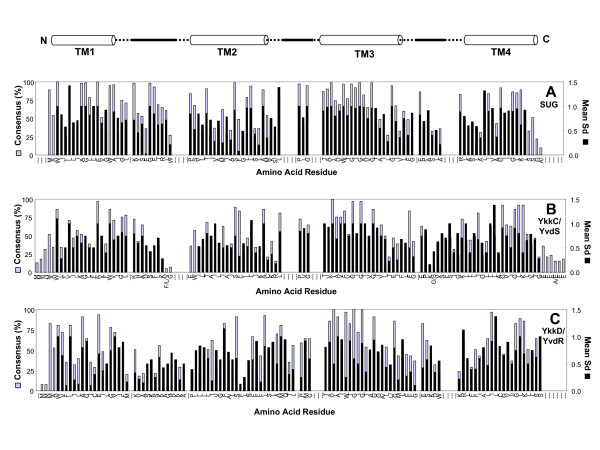
**Summary of SUG and PSMR (YkkC/YvdS and YkkD/YvdR) amino acid residue consensus and mean synonymous substitutions (Mean Sd) observed at each position within their overall sequence alignments**. Data is shown for SMR members in each panel as follows: **A**) SUG subclass, **B**) PSMR subclass members YkkC/YvdS, and **C**) PSMR subclass members YkkD/YvdR. The amino acid(s) that occurred with the highest frequency at each position within the alignment is indicated on the x-axis below each bar and dashes indicate positions lacking amino acid alignment. Each bar represents the degree of conservation of the listed amino acid(s) below based on its percentage (Consensus %; grey bars) according to the left- hand y-axis. Mean Sd values shown on the right- hand y-axis were calculated from Syn-SCAN pairwise comparisons of Sd values for 20 SMR homologues from each SMR subclass alignment. Mean Sd values, represented as black bars, indicate the level of observed synonymous nucleotide substitutions within the codon for each consensus amino acid listed below.

**Figure 3:**
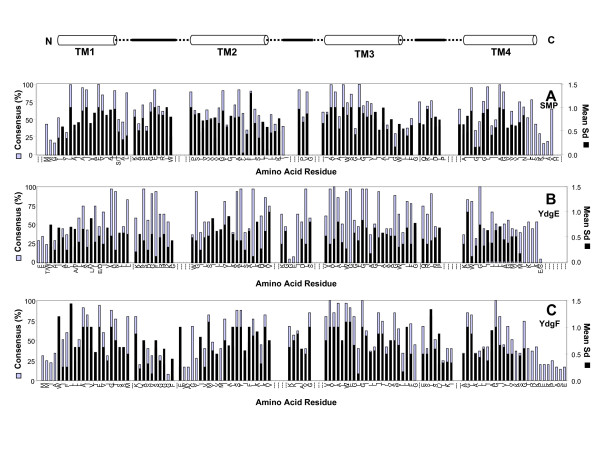
**Summary of SMP and PSMR (YdgF and YdgE) amino acid residue consensus and mean synonymous substitutions (Mean Sd) observed at each position within their overall sequence alignments**. Data is shown for SMR members in each panel as follows: **A**) SMP subclass including integron encoded Qac members, **B**) PSMR subclass member YdgE, **C**) PSMR subclass member YdgF. Figure legend details are identical to those described for Figure 2; refer to this figure for details.

**Figure 4:**
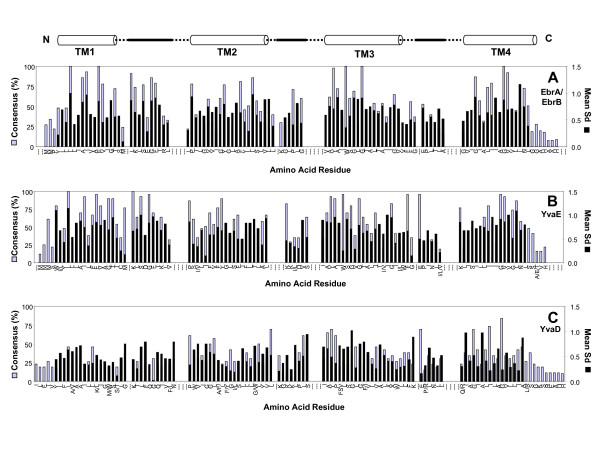
**Summary of PSMR (EbrA/EbrB, YvaE and YvaD) amino acid residue consensus and mean synonymous substitutions (Mean Sd) observed at each position within their overall sequence alignments**. Data is shown for SMR members in each panel as follows:**A**) PSMR subclass members, EbrA and EbrB, **B**) PSMR subclass member YvaE, and **C**) PSMR subclass member YvaD. Figure legend details are identical to those described for Figure 2; refer to this figure for details.

To investigate how selective pressure affects SMR subclass differentiation, we examined both SMR protein and nucleotide alignments in further detail. After generating an overall amino acid consensus from the protein alignment of each SMR subclass member we examined the extent of amino acid conservation at each position within loop and TM regions. Mean synonymous nucleotide substitution (Sd) values were also calculated at each codon position in the SMR nucleotide alignment using Syn-SCAN analysis to determine the selective pressures exerted on each residue of the protein. Sd values were determined from pairwise comparisons of selected members (10–20 species/SMR subclass) and then averaged to generate the mean Sd at each codon. A summary of both analyses are shown in Figures 2, 3, 4, where a distinct pattern of residue conservation can be observed for each SMR subclass member we analyzed.

Both SUG and SMP subclasses demonstrated a unique amino acid consensus in addition to the core SMR motif previously reported [9] (Figures 2A and 3A). Additionally, the amino acid consensus derived for each PSMR subclass member appears to be unique and generally maintains the core SMR motif within each predicted loop and TM strand. PSMR members also displayed residues particular to either the SMP (pairs YdgE/YdgF; Figures 3B and 3C and EbrA/EbrB; Figure 4A) or SUG (YkkC/YvdS; Figure 2B and YkkD/YvdR; Figure 2C) consensus motifs and in some cases both, as shown for YvaE (Figure 4B). The predominance of either SMP or SUG consensus motifs for paired PSMR subclass members in particular provides further support for gene duplication within the SMP or SUG clade (see Figure 1).

A striking feature within each panel of Figures 2, 3, 4, is the overall periodicity of amino acid residue conservation within each TM strand of the protein. Such periodicity was demonstrated in earlier analysis of SUG and SMP homologues performed by [47]. Examination of the amino acid consensus maps of SUG and SMP homologues in Figures 2, 3, 4 indicate that each TM strand appears to have the highest amino acid conservation in a periodic pattern similar to the turn of an α-helix. This periodicity shows that one face of each predicted TM α-helix (TM 1–4) is more conserved within all four TM strands. Moreover, this residue periodicity varies at different positions when comparing the SUG consensus map to SMP (Figures 2A and 3A). Hence, SUG homologues may have different structural orientations within the TM strands than SMP subclass members.

Periodic α-helix conservation is also maintained in many PSMR subclass consensus maps but distinctly varies from TM strand to TM strand, where the least TM conservation is observed in the last strand (TM4) (Figures 2, 3, and 4). Predicted loop/turn regions of most PSMR members, particularly loops 1 and 2, show higher levels of sequence divergence from SUG and SMP subclasses and generally much longer loop regions than those predicted for either SMP or SUG. Furthermore, comparisons of PSMR subclass pairs together show TM strands with opposing high levels of amino acid conservation. To clarify, YdgE and YdgF members have obvious differences in TM strand amino acid conservation patterns; TM strands 1 and 4 of YdgE have far fewer conserved residues than TM strands 1 and 4 of YdgF (Figures 3B and 3C). This is also observed in TM strands 1 and 3 of YkkD/YvdR which have more highly conserved residues than in TM strands 1 and 3 of YkkC/YvdS (Figures 2B and 2C). These amino acid consensus differences agree with results obtained from phylogenetic associations and estimated dS/dN values determined for these pairs (Figure 1; Additional file 2). Therefore, the consensus maps of PSMR pairs show that one member of a given pair or more specifically certain TM strands within each PSMR pair are maintained under far less selective pressure.

Amino acid sequence alignments for the PSMR subclass members EbrA and EbrB were difficult to distinguish from each other since they both had similar residue conservation patterns, especially when Actinobacterial EbrA and EbrB homologues were examined. The inability to reliably separate EbrA/EbrB homologues from each other forced an initial comparison of these PSMR members altogether. High amino acid conservation is observed for EbrA/EbrB homologues within the loop and C-terminus regions of the protein (Figure 4A). Conservation within these regions of EbrA/EbrB support site-directed mutagenesis experiments of Bsu-EbrA and Bsu-EbrB that demonstrate the importance of the loop and C-terminus regions on protein function as a pair; their removal in either EbrA or EbrB resulted in individual variants that could confer isogenic resistance to the host [23]. Closer examination of Actinobacterial EbrA/EbrB homologue alignments only showed reduced amino acid consensus values towards the end of the third TM strand in comparison to the overall EbrAB amino acid residue analysis (data not shown). This low overall residue consensus in the C-terminal portion of TM3 for Actinobacterial EbrA/EbrB homologues only suggests that this TM3 strand could be diverging toward a particular function specific for Actinobacteria only.

Despite having a distinct amino acid consensus motif from SMP and SUG, YvaE demonstrated a similarly strong periodic amino acid conservation pattern, particularly within predicted loop and turn regions. As expected, YvaD homologues show low overall amino acid conservation throughout the entire protein with the exception of the three to four moderately conserved residues at the ends of the last two TM strands towards the C-terminus. This clearly indicates that YvaD has diverged dramatically from other PSMR members. An important feature of YvaD homologues to consider is loss of the highly conserved residue E14 (according to Eco-EmrE); yet conserved residues within the C-terminal residues are only partially maintained. These infrequently conserved residues are shared with both YvaE and SMP subclass members strongly supporting the idea that YvaD likely originated from YvaE but is maintained under very little selective pressure within the hosts.

Examination of mean Sd values at each codon position within all SMR nucleotides demonstrated strong agreement to its corresponding amino acid consensus value within predicted loop and TM regions (Figures 2, 3, and 4). This result is important since the differences in codon usage by the various Archaeal and Bacterial hosts may have reduced the mean Sd values particularly at conserved amino acid residue positions. One explanation to account for these high mean Sd values is found when comparing the close relationship of integron and plasmid encoded SUG and SMP homologues to chromosomal SMR sequences. Lateral gene transfer of SMR sequences to evolutionarily unrelated hosts would act as its own codon bias keeping Sd values high and has been shown in studies of other horizontally transferred sequences (for an example refer to [38]).

The mean Sd values observed for both SUG and SMP subclass sequences show a similar α-helical periodicity pattern to conserved amino acid residue positions strongly validating the observation that specific TM α-helix faces are highly conserved. However, it should be noted that there are also occasional amino acid positions in each SMR subclass consensus where codons corresponding to loop or TM strand regions have high mean Sd values but very low amino acid consensus value and vice versa. For example, numerous positions in TM4 of the SUG consensus map have high mean Sd values but low values for its conserved amino acid at the same position (Figure 2A). Conversely, what would appear to be a highly conserved amino acid at particular positions in loop 1 of the YdgF consensus map show low mean Sd values (≤ 0.6) (Figure 3B). Together, this supports the observation that many positions and regions among all the SMR subclass members are undergoing different rates of divergence and reflect regions of the protein that may be actively evolving or undergoing sequence optimization for particular host.

### SMR homologues are linked to metabolite transport and regulation based on their association with putative operons

Experiments implicating SMR protein involvement in toxic metabolite transport suggest broader roles for this family of proteins during host metabolite regulation. Although members of the SMR protein family are known for conferring multidrug resistance to its host organism, very little is known about potential 'natural' substrates. PSMR members Eco-YdgE and Eco-YdgF and *A. nicotinovorans *plasmid pAO1 encoding NepA and NepB have specifically demonstrated efflux of potentially toxic metabolites such as spermidine and nicotine intermediates, respectively [10,11]. In addition to these metabolites, SUG members potentially influence the cellular chaperone activity, namely the GroES-GroEL complex [13,14,48] and efflux a limited subset of QAC compounds [14]. To explore the association of SMR proteins to metabolite trafficking and chaperones, we examined the arrangement of open reading frames (ORF) at the genomic loci of identified SMR subclass homologues (a total of 283 genomic loci from completed genomes of Archaea and Bacteria). Our goal was to explore the degree of association SMR subclass members had with other ORFs to identify putative substrates for these transporters.

After completing our SMR genomic loci survey, many trends in SMR genomic arrangement were noted. The first common characteristic identified from these surveys was detection of horizontal gene transfer genes that encoded for integrases, transposons, insertion sequences (IS), plasmid maintenance genes, and bacteriophage replication and coat proteins downstream or upstream from the SMR sequence (within our 10 ORF radius cut-off), strengthening our arguments for horizontal SMR inheritance as shown in the phylogram (Table 2). Mobile genetic elements were found to associate with all SMR homologues at a frequency of 22% within a 10 ORF radius of surveyed genomic loci (Table 2) and upon randomly expanding our genomic loci survey to a 25 ORF radius we saw an increase in mobile element detection (~34%) (data not shown).

The second characteristic we observed upon examining SMR genomic loci was frequency of SMR subclass co-occurrence to various metabolite biosynthetic genes (Table 2). In general, all the ORFs we could putatively identify associated with SMR genes from this survey fell into the following categories; amino acid transport and biosynthesis, vitamin and cofactor transport and biosynthesis, fatty acid biosynthesis, nucleotide biosynthesis, and multidrug resistance. Other genes identified were commonly associated with various mobile elements and were indirectly tied to metabolite transport, namely protein folding (chaperones and post-translational modification), signal transduction/two-component cell regulators, cell wall biogenesis and degradation, and DNA/RNA replication and regulation. Although many of these associations are likely random and are not expected to have a direct effect on SMR function, some ORF occurred too frequently with SMR genes to be discounted outright and are summarized in Table 2.

The last final characteristic observed from the surveys of genomic loci showed that PSMR homologues had characteristic arrangements in gene pair at the locus. This observation may reflect their potential to act as predecessors of much larger transporters. To clarify this statement, the PSMR gene pairs, *ydgE*/*ydgF *and *ykkC*/*ykkD*, were shown to frequently overlap (95%–98% surveyed respectively) in all proteobacterial genomic loci we surveyed. This contrasted the PSMR homologues *ebrA*/*ebrB *that did not frequently overlap and were often separated by other ORFs within their putative operons. In our survey EbrA/EbrB homologues were also found to correspond to a single gene only within species from Actinobacteria (32%), Clostridia (14%), Cyanophyceae (13%), Chloroflexi (25%) and Planctomycetacia (33%). This observation is similar to the situation we described for *yvaE *and *yvaD *pairing suggesting that some isogenic *ebrA*/*ebrB *homologues may possess functional activity alone or frequently undergo losses of either pair.

It is important to mention again that these surveys in no way prove the functional activities of the various SMR subclasses, but identify potential substrates or activities that could and should be examined experimentally. It also reaffirms that the distribution of particular SMR subclasses are focusing in on specific Archaeal and Bacterial groups, in addition to their shared proximity with other organisms enabling SMR mobile genetic element exchange.

### SMR family proteins demonstrate regions of conservation with other larger multidrug and metabolite transporters

SMR proteins are one of the 14 families that encompass the DMT superfamily [30] and include chloramphenicol resistance proteins of the RarD family and proteins such as amino acid metabolite efflux pump (EamA) and PhoPQ-activated protein (PagO) of the drug/metabolite exporter (DME) family. Since SMR proteins are thought to act as progenitors of these larger and topologically diverse multidrug transporter families, an evolutionary model was proposed to explain how SMR proteins may have contributed to the formation of families including BAT and DME [30]. Based on this model, topological rearrangements of SMR sequences and/or fusions of a TM forming domain are suggested to have contributed to the five TM stranded BAT family which upon fusion with itself led to the formation of 10 TM stranded DME proteins (SMR ↔ BAT → DME). By extension of this model, other larger TM stranded proteins such as major facilitator superfamily (MFS) could be formed by the fusion of 2 BAT sequences (2 BAT ↔ MFS). Support for the evolutionary model proposed by Jack *et al*. 2001 was shown from alignments of SMR TM domains to portions of DME and BAT family proteins in either the N- or C-terminus region of the protein. According to these alignments SMR proteins shared the greatest amount of sequence identity to the C-terminus regions of BAT or DME families [30]. Hence, if SMR sequences assemble to form larger transporters, we would expect to identify TM domain regions of protein within DMT family members with high sequence similarity as SMR sequences.

**Figure 5:**
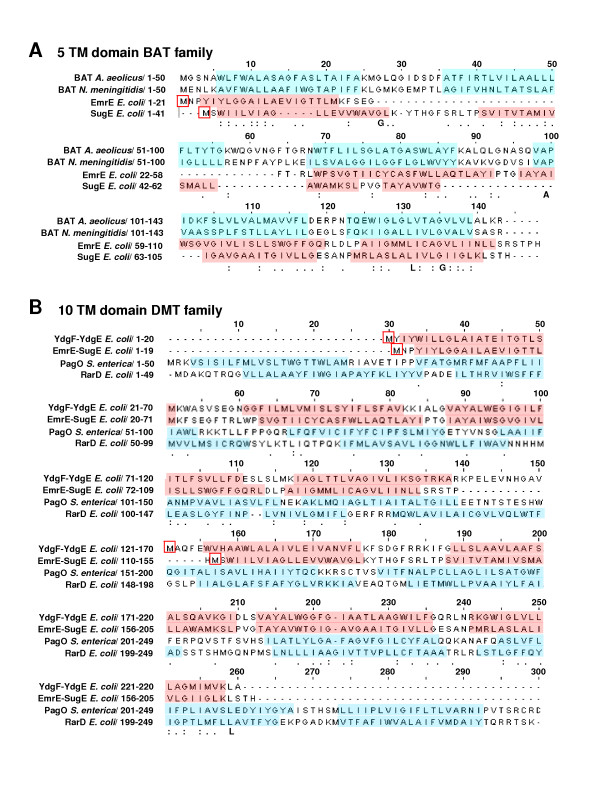
**TM domain alignments of SMR proteins to drug and metabolite transporters from BAT and DMT protein families**. Protein alignments were performed using ClustalW and manual editing using GeneDoc (v 2.5.010 [64]). Artificial fusions proteins of all SMR proteins from each subclass were performed (data not shown) and selected alignments for the fused protein pairs Eco-YdgF to Eco-YdgE and Eco-EmrE to Eco-SugE are shown in panel B. TM domains for SMR proteins are highlighted in red whereas all other predicted TM domains are highlighted in blue. M residues are boxed in red in each alignment in both panels to indicate the starting residue for SMR fusion sequences. Conserved residues at a given position within TM domains are indicated by the amino acid letter below the sequence and moderate to high amino acid similarity is indicated by a single or two dots respectively at each position. Panel A indicates the alignment of Eco-SugE and Eco-EmrE sequences to predicted TM domains of BAT family proteins from Archaeal *Aquifex aeolicus *(AAC07598; 143 a.a.) and Bacterial *Neisseria meningitidis *(AAF42175; 143 a.a.). Panel B shows alignments of Eco-EmrE to Eco-SugE and Eco-YdgF to Eco-YdgF fusions is aligned with the DMT superfamily members *E. coli *RarD (ZP_03000057; 286 a.a.) and *Salmonella enterica *PagO (ZP_03162845; 304 a.a).

**Figure 6:**
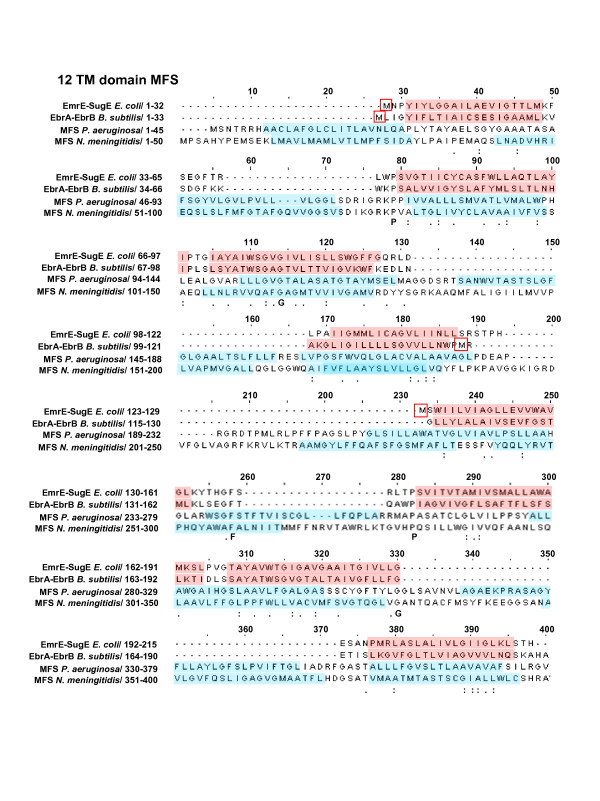
**TM domain alignments of SMR proteins with MFS members**. Artificial fusions proteins of all SMR proteins from each subclass were performed (data not shown) and selected alignments for the fused protein pairs Bsu-EbrA to Bsu-EbrB, and Eco-EmrE to Eco-SugE are shown with MFS transporters from *Pseudomonas aeruginosa *(NP_253969; 389 a.a) and from *N. meningitidis *(NP_273492; 400 a.a). For further details describing this figure refer to the description provided in Figure 5.

To explore potential SMR TM domain remnants within BAT and DMT superfamily members we created artificial fusions of SMR subclass pairs together (using *E. coli *and *B. subtilis *homologues) in both heterogeneous (e.g. Eco-EmrE to Eco-SugE) and homogenous (Eco-EmrE to Eco-EmrE) protein combinations to serve as seed sequences for phi-BLAST analyses of genomic databases. These searches identified numerous TM domain regions within SMR fusions that had similarity to various DMT superfamily members in addition to members of MFS (Figures 5 and 6). Sequence similarity within aligned TM domains of SMR protein with Archaeal and Bacterial BAT homologues were higher than expected (≤ 25%) based on previous examination [30]. Regions with the least TM domain alignability between SMR and BAT proteins were observed for the predicted TM2 strand of all BAT sequences examined (Figure 5A). SUG protein sequence alignments to BAT proteins showed SUG TM1 and N-terminal TM2 strand splitting within the alignment of BAT TM strands 1–3. As observed by Jack *et al*. 2001 [30], the highest proportion of amino acid residue conservation was found within the C-terminus alignments of BAT and SMR. Based on C-terminal TM domain conservation, the model by Jack *et al*. 2001 suggested that the evolutionary gain of another TM domain was likely to occur at either end of the SMR sequence [30]. Our results support this (SMR → BAT) model and suggest that SMR TM domain splitting or TM domain duplication gain within the SMR N-terminal region specifically resulted in BAT family formation. To determine if the SMR N-terminal TM domain region is poorly aligned to larger multidrug efflux protein alignments were preformed using homo- or hetero fusion SMR proteins. In general, almost all SMR fusions we examined demonstrated a similar TM domain alignment pattern to 10 TM strand DME members (in this case RarD and PagO shown in Figure 5B) and to 12 TM stranded MFS members (Figure 6). However, we also noticed that the order of the hetero-SMR protein fusions resulted in higher alignment scores to DMT TM strand alignment. For example, *E. coli *YdgF-YdgE (18%) but not *E. coli *YdgE-YdgF (10%) alignment to DMT superfamily member RarD (Figure 5B) resulted in higher sequence similarity values (data not shown). As observed for SMR-BAT alignments (Figure 5A), the SMR fusion 'N-terminus regions' (TM1–2 and TM5–6) showed the poorest TM domain alignments to N-terminus and central regions of DMT (TM1–2 and TM5–6) and MFS (TM1–3 and TM6–8) sequences. It is important to note that the lack of TM domain alignability within SMR TM domains 1–2 may simply be due to the higher levels of polar and aromatic residue conservation within these regions in comparison to TM strands 3–4 (more hydrophobic residues) (Figures 2, 3, and 4), making C-terminal alignments in SMR TM domains 3–4 more favourable to larger TM domain transporters based on hydrophobicity alone.

**Figure 7:**
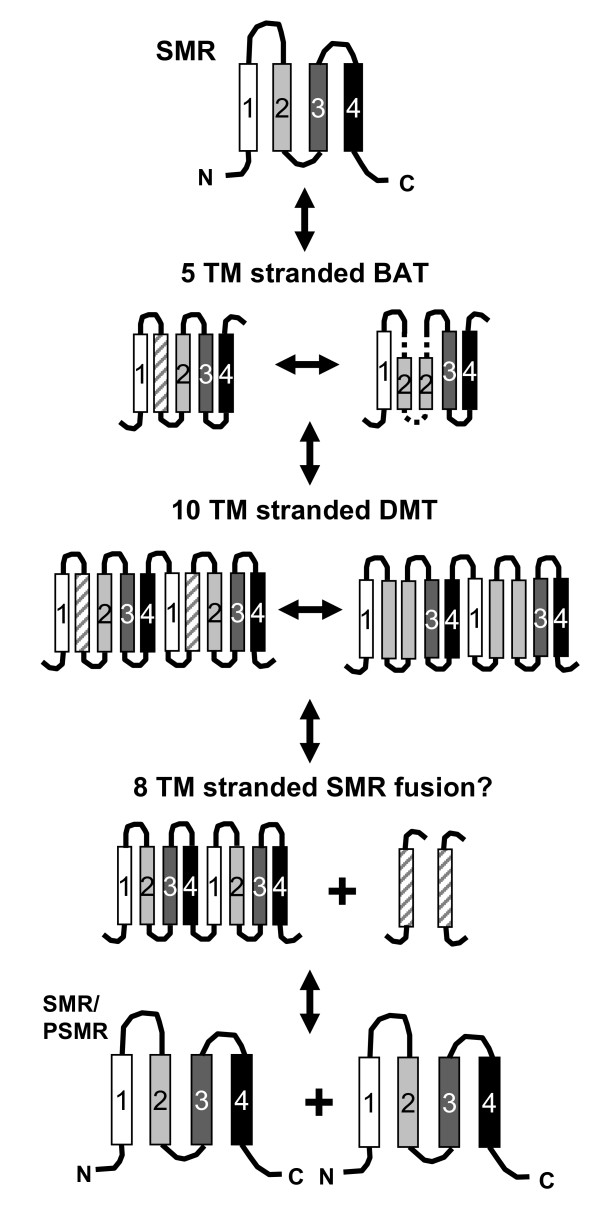
**A diagram cartoon showing SMR protein TM strand evolution to generate members of the BAT, and DMT protein families**. SMR TM domains conversion is based on TM alignments to larger drug transporters shown in Figure 5. TM strands from a putative SMR protein are shown as filled rectangles and numbered according to their position within the protein. Unnumbered vertically lined rectangles indicate TM strands gained within BAT and DME proteins. N- and C-termini are no longer shown in the figure to account for topological orientations changes that may contribute to this process. An alternative SMR/PSMR protein fusion model is also shown based on SMR fusion alignments which may also contribute to a 10 TM stranded DMT protein. For this model, fusion of two PSMR sequences and the subsequent gain of TM domains show the formation of 10 TM domain DMT transporters.

In light of the SMR TM domain alignments to larger multidrug transporters, we propose an extension of the SMR multidrug origin model (Figure 7). This updated model highlights TM domains in the N- (TM 1–2) and C-termini (TM 3–4) in SMR protein and its fusions that were identified within transporters of DMT and MFS. The observations noted from the SMR-BAT/DMT/MFS alignments (Figures 5 and 6) provide further support for the evolutionary models proposed by Jack *et al*. 2001 and Rapp *et al. *2007 [30,49]. It is important to note that additional transporters such as sodium/proline symporters (proline permease PutP of the sodium solute sugar transporter family) and other amino acid exporter proteins (such as YeeA) from Gram-Positive and Gram-Negative hosts outside of the DMT superfamily were also identified from our phi-BLAST searches and shown to similarly align according to TM domain (data not shown). This extends the scope of the current evolutionary model and also supports our bioinformatics genomic loci surveys that link SMR protein involvement to amine metabolism (Table 2). We predict that SMR gene duplications of either SMP or SUG subclasses gave rise to not only the PSMR subclass members but PSMR proteins themselves may have contributed to DMT superfamily diversity. The TM domain alignments of artificial PSMR fusions to similar TM regions within MFS and DME family members support this prediction.

The ability of SMR proteins to adopt dual-topologies within the plasma membrane as determined experimentally [28,49,50] would also make the SMR family an ideal candidate as a molecular building block for larger TM domain multidrug transporters. Unfortunately, the low degree of sequence identity (ranging from 10 – 30%) within the SMR to BAT, DMT or MFS alignments (Figures 5 and 6) and the relatively poor overall sequence conservation of R and K amino acid residues (topological determinants of the 'positive inside' rule as discussed in [51,52]) observed from our alignment analysis in Figures 2, 3, and 4 make it difficult to draw reliable conclusions that address the controversy surrounding SMR topology and subunit arrangements [53]. Evidence for and against SMR dual-topology can be equally taken from this work, making it insufficient to resolve specific SMR topology. To highlight this point, the functional asymmetry of YdgE and YdgF, discussed in sections above, strongly supports a dual-topology of PSMR proteins, but our SMR fusion alignments (Figures 5 and 6) and dS/dN results (Table 3 and Additional file 2) make topological orientation conclusions for both SMP and SUG subclasses uncertain due to the low overall degree of amino acid residue conservation in loop and turns. It should also be noted that our findings could easily be re-interpreted as MFS/DMT/BAT → SMR evolution, since degeneration and partial sequence losses within DMT superfamily members, such as PagO or RarD, via horizontal gene movement could have also resulted in a functional truncation leading to the current SMR family. What is clear from this study is that SMR family members are justifiably placed within the DMT superfamily and potentially share functional and structural characteristics with other DMT family members making SMR proteins essential for future experimental studies.

## Conclusion

In summary, SMR homologues grouped into two major clades based on our phylogram: SUG and SMP (Figure 1; Additional File 1). We have also suggested that gene duplication events within each clade likely resulted in the emergence of PSMR members among particular bacterial classes. Based on our analyses of both amino acid and nucleotide conservation among SMR homologues there is convincing evidence to support that chromosomally encoded SUG, SMP and PSMR have diverged in both sequence and function from their integron encoded counterparts. The high frequency of lateral gene transfer and rapid sequence divergence within the identified SMR homologues in Archaeal and Bacterial genomes precluded the reliable identification of a single common SMR progenitor. Similar to other associated multidrug resistance integron encoded genes such as sulphonamide resistance *sull *[54] or chloramphenicol resistance *cmlA *[5,55], SMR family origins will be difficult to determine due to the multitude of pressures exerted on the gain and loss of these sequences as they move from integrons, super-integrons, plasmids, megaplasmids, and chromosomes. However, the inherent plasticity of these SMR homologues based on their unique distribution within various microorganisms, small size, and structural similarity to other larger drug and metabolite transporters make them one of the most likely progenitors of α-helical drug/metabolite transporters.

Despite the apparent rapid emergence of drug resistance, numerous multidrug resistance mechanisms have been shown to predate anthropogenic antibiotic usage, strongly suggesting that SMR genes have existed long before human antibiotic and antiseptic introduction [56]. Bacterial adaptation to antimicrobial compounds secreted by plants (for example QAC osmoprotectants such as betaines) and fungi (for example antibiotic β-lactam derivatives) are some of the many toxins that have likely contributed to the development of current SMR similar to other resistance mechanisms (as reviewed by [57]). Taking this into account, the variation in SMR subclass distribution may make sense by simply examining the environment of the Bacteria and Archaea who harbour them. Controlling QAC and lipophilic compound concentrations by the host microorganism living in soil environments, areas enriched with industrial pollution, in hypersaline environments, or both opportunistic and strict pathogens is advantageous and would explain why SMR distribution via lateral gene transfer is an 'economic tool' due to its broad resistance to toxins for its size.

## Methods

### SMR protein dataset selection and SMR subclass designation

SMR sequences used for this study were obtained from NCBI genomic blast surveys (tBLASTn and BLASTp) using SMR sequences functionally characterized for transport activity [11,14,22,26,58]. Chromosomally encoded SMR homologues were identified in a total of 340 Archaeal and Bacterial species using query SMR sequences from *Bacillus subtilis *(Bsu-EbrA NP_389612 and Bsu-EbrB NP_389611; Bsu-YkkC NP_389192 and Bsu-YkkD NP_389193; Bsu-YvdR NP_391330 and Bsu-YvdS NP_391329; Bsu-YvaE NP_391237 and Bsu-YvaD NP_391236) and *Escherichia coli *(Eco-EmrE NP_415075; Eco-SugE NP_418572; Eco-YdgE/MdtI NP_416116 and Eco-YdgF/MdtJ NP_416117) [59]. As a result, a total of 685 non-identical SMR sequences (as of March 2008) were identified from the query SMR sequence blasts with e-values of ≤ 1 × 10^-4 ^and served as the cut-off value for positive SMR identification. The 685 putative SMR protein sequences were aligned using ClustalW [60,61] and TCoffee [62] (data not shown). This aligned data included smaller SMR protein datasets used in previous analyses [9,12,26,37,63]. The 685 protein ClustalW alignment was refined manually using the editing program GeneDoc (v 2.5.010 [64]). SMR subclass designations for all of the 685 SMR protein sequences were assigned based on their sequence similarity to the SMR protein seed sequences within the protein sequence alignment, and their evolutionary relationship to SMR subclass members using phylogenetic analysis by the Neighbour-Joining (NJ) program available from ClustalW [61]. The results of this SMR distribution survey are summarized in Table 1. An amino acid residue consensus was also determined for each SMR subclass from our alignment and the results are presented as percent occurrence in Figure 5.

SMR proteins alignments to BAT, DMT and MFS transporters were performed using ClustalW and manual alignment using the editing program GeneDoc (v 2.5.010 [64]). BAT, DMT and MFS sequences were selected based on their identity to the original SMR seed sequences through phi-BLAST searches available through NCBI. Transporter domains with ≤ 30% TM domain sequence similarity to the SMR TM domains. Artificial SMR fusions using homogenous (for example Eco-EmrE to Eco-EmrE) or heterogeneous (Eco-EmrE to Eco-SugE) sequences aligned to all the identified transporters from phi-BLAST (NCBI) searches. In some cases, TM domains for BAT, DMT and MFS proteins required TM domain prediction and were performed using online prediction algorithms TMpred [65], and transmembrane hidden Markov Model (TMMOD) [66].

### Synonymous/non-synonymous nucleotide substitution analysis of SMR sequences

SMR nucleotide sequences used for Syn-SCAN analysis [67] were selected from each SMR subclass protein dataset to represent the taxonomic variation from Archaea and Bacteria and represented those members with the most SMR subclass diversity. Selected nucleotide sequences were aligned manually using GeneDoc (v 2.5.010 [64]) by their codon using the 685 SMR protein alignment as a reference. Syn-SCAN analyses were performed on a minimum of 20 aligned SMR nucleotide sequences from each SMR subclass and additional sequence comparisons were made to plasmid/integron SMR homologues. Full length SMR nucleotide sequences were aligned according to Eco-*emrE *codon 3 (W3) to codon 107 (L107). The distribution of point mutations from SMR sequences was examined spanning a 300 bp region which included nucleotides that encoded each of the four predicted transmembrane strands as well as loop/turn regions and truncated poorly conserved codons from the encoded N- and C-termini. For *yvdR*/*yvdS *and *ebrA*/*ebrB *sequences, poor alignment of three codons within the region encoding the first putative loop1 had to be removed. The rate of synonymous to non-synonymous changes (dS/dN) was determined using the online Syn-SCAN program website [67-69] and the values of SMR sequence pairs representing each of the three SMR subclasses are shown in Table 3 and Additional file 2. Large values of dS/dN (> 1.0) imply that the encoded protein is selectively constrained and indicates that selective pressures are occurring within the sequences that minimize the number of amino acid changes, thus retaining the function and/or structure of the protein. Average dS/dN values were also calculated for all nucleotide sequences within each SMR subclass and summarized in Table 3 and Additional file 2. The observed number of synonymous nucleotide substitutions (Sd) at each codon within each SMR subclass alignment was also calculated and the mean Sd values are summarized in Figures 2, 3, and 4.

### SMR protein phylogenetic tree generation

To examine the evolutionary relatedness of the all SMR subclass members assigned from the original 685 SMR protein alignment, a smaller alignment of 338 SMR protein sequences was prepared to adequately represent the taxonomic diversity of sequence origin (Additional File 1). Phylogenetic trees based on Neighbour-Joining (NJ) were generated for the smaller SMR protein sequence data set (n = 338) using the program PROTDIST [Jones-Thornton-Taylor setting and NEIGHBOR (NJ setting)]; these programs are part of the program package PHYLIP (Version 3.63, [70,71]). Bootstrap replicates (1000) were generated using SEQBOOT (PHYLIP) and evaluated with NJ analysis in combination with the CONSENSE program (PHYLIP) for obtaining a majority rule consensus tree. All dendrograms presented were drawn with the TreeView program (Version 1.6.6, PHYLIP) using the PHYLIP tree out files and annotated manually. Among the Archaeal genomes explored, only three Crenarchaeal sequences were identified with e-values of ~5 × 10^-3 ^and each sequence produced highly similar tree arrangements and bootstrap values (data not shown). Therefore, *Archaeoglobus fulgidus *QacE sequence was selected arbitrarily as the outgroup for the final tree. The results of these analyses are presented in Figure 1 and Additional File 1.

### SMR genomic loci surveys

The genomic loci of SMR subclass members were examined from sequenced host microorganisms using NCBI online Entrez Genome Sequence Viewer . Genes peripheral to the SMR gene were surveyed using a 10 gene radius. Putative operons were deduced from the genomic sequence within the 10 gene radius using the online promoter scanning programs PromScan  and Virtual Footprint . The frequency of SMR occurrence in various putative metabolite operons were reported in Table 2 as the percentage of total loci surveyed. The frequency these metabolite genes occur within the gene radius surveyed was also reported in Table 2, by the percentage of total loci surveyed for each SMR subclass.

## Abbreviations

The following abbreviations used in the manuscript are listed here in alphabetical order: BAT: (Bacterial/Archaeal transporter); DME: (drug metabolite exporter); DMT: (drug metabolite transporter); dS/dN: (rate of synonymous to non-synonymous mutations); MFS: (Major facilitator superfamily); PSMR: (paired small multidrug resistance); Sd: (number of synonymous nucleotide substitutions); SMP: (small multidrug proteins); SMR: (small multidrug resistance); SUG: (suppressor of *groEL *mutation); TM: (transmembrane); QAC: (quaternary ammonium compounds).

## Authors' contributions

The dataset collection and bioinformatics analyses were performed by DCB. Manuscript writing including Table and Figure generation was performed by DCB and manuscript editing was performed by both RJT and DCB.

## Supplementary Material

Additional file 1 **A phylogenetic tree of the SMR protein family**. The rooted phylogenetic tree is based on Neighbour-Joining analysis of 338 SMR protein sequences. The Archaeal *Archaeoglobus *QacE sequence served as an outgroup for this analysis. In some cases, individual PSMR sequences are highlighted by parenthesis and listed around branches to indicate important groupings. One thousand bootstrap replicates were performed and confidence values (by percentage) are listed beside their respective nodes above 59%. Plasmid and integron encoded SMR proteins are underlined. SMR sequence accession numbers are indicated adjacent to its genus and species name. Click here for file

Additional file 2 Summary of synonymous to non-synonymous nucleotide substitution patterns within PSMR subclass members. Click here for file

Additional file 3 **An alignment of 338 SMR protein sequences identified from BLAST surveys of completed Archaeal and Bacterial genomes**. The 338 SMR protein sequence alignment was truncated from a larger alignment of 685 SMR sequences that was generated using a manually edited ClustalW alignment. It is important to note that this alignment may contain truncated versions of some SMR sequences. Click here for file
