# Supplementary figures and images for: Diversity and evolution of the small multidrug resistance protein family
Source: BMC Evol Biol. 2009 Jun 23;9:140. doi: 10.1186/1471-2148-9-140 (PMC2716321; doi:10.1186/1471-2148-9-140)

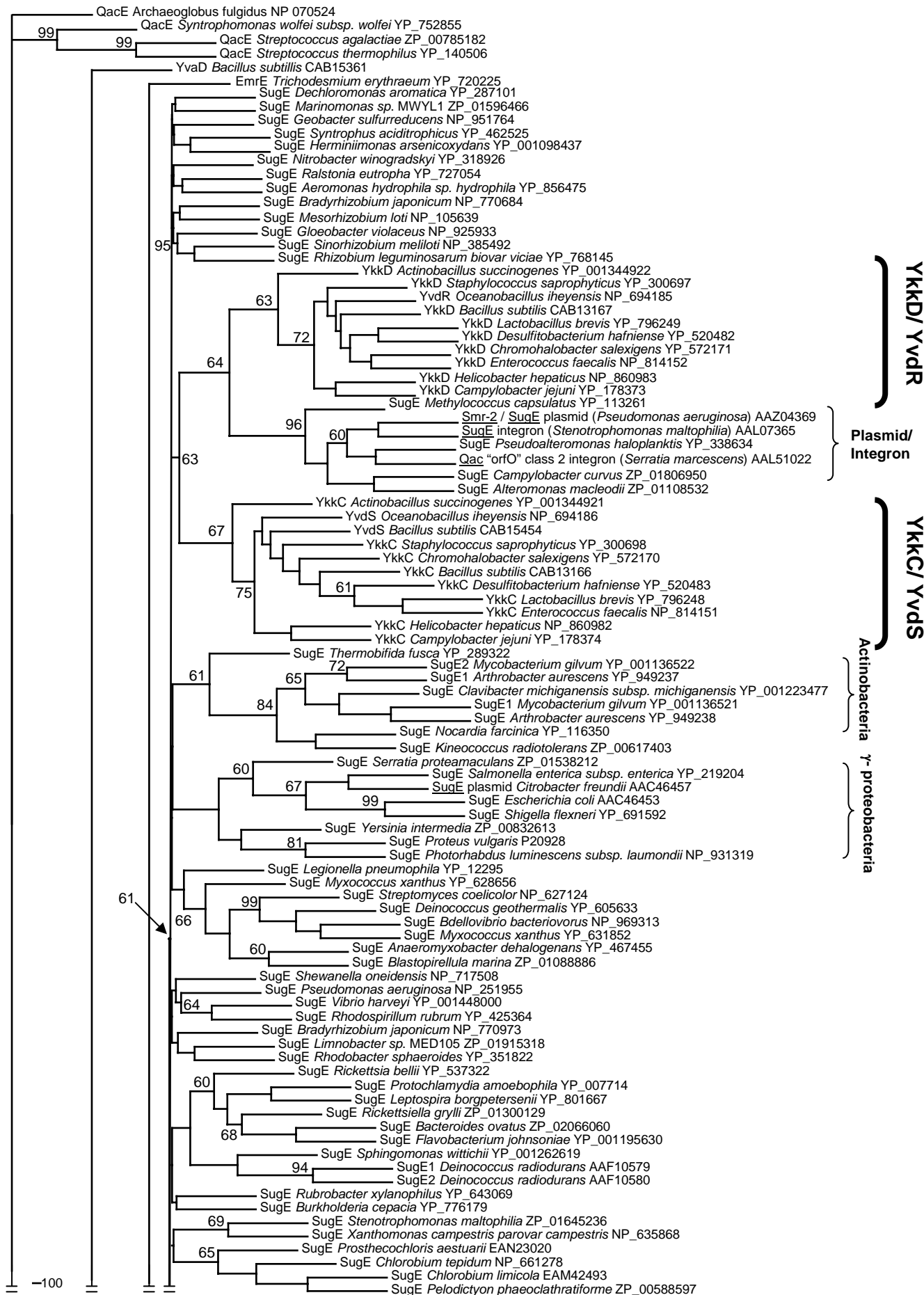

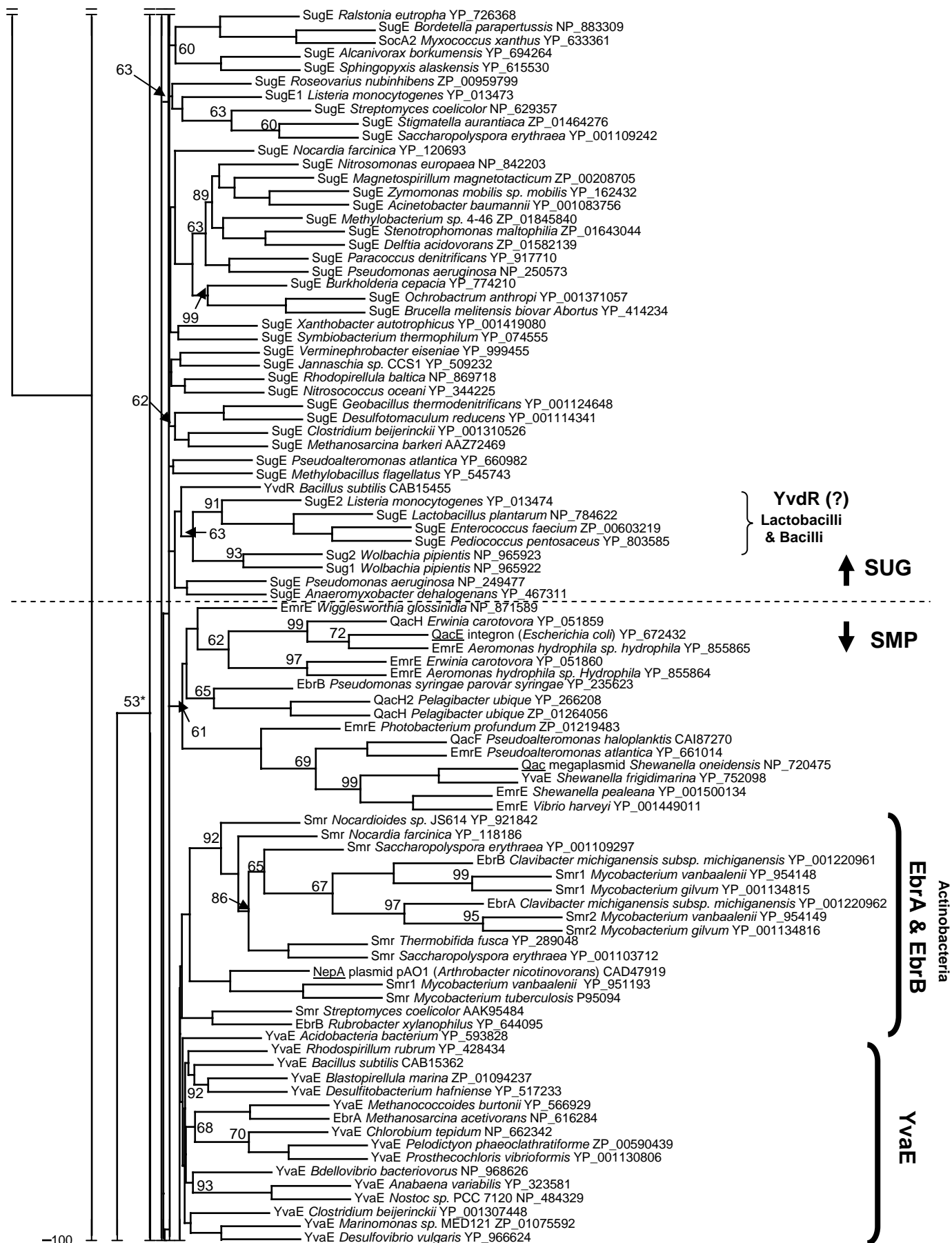

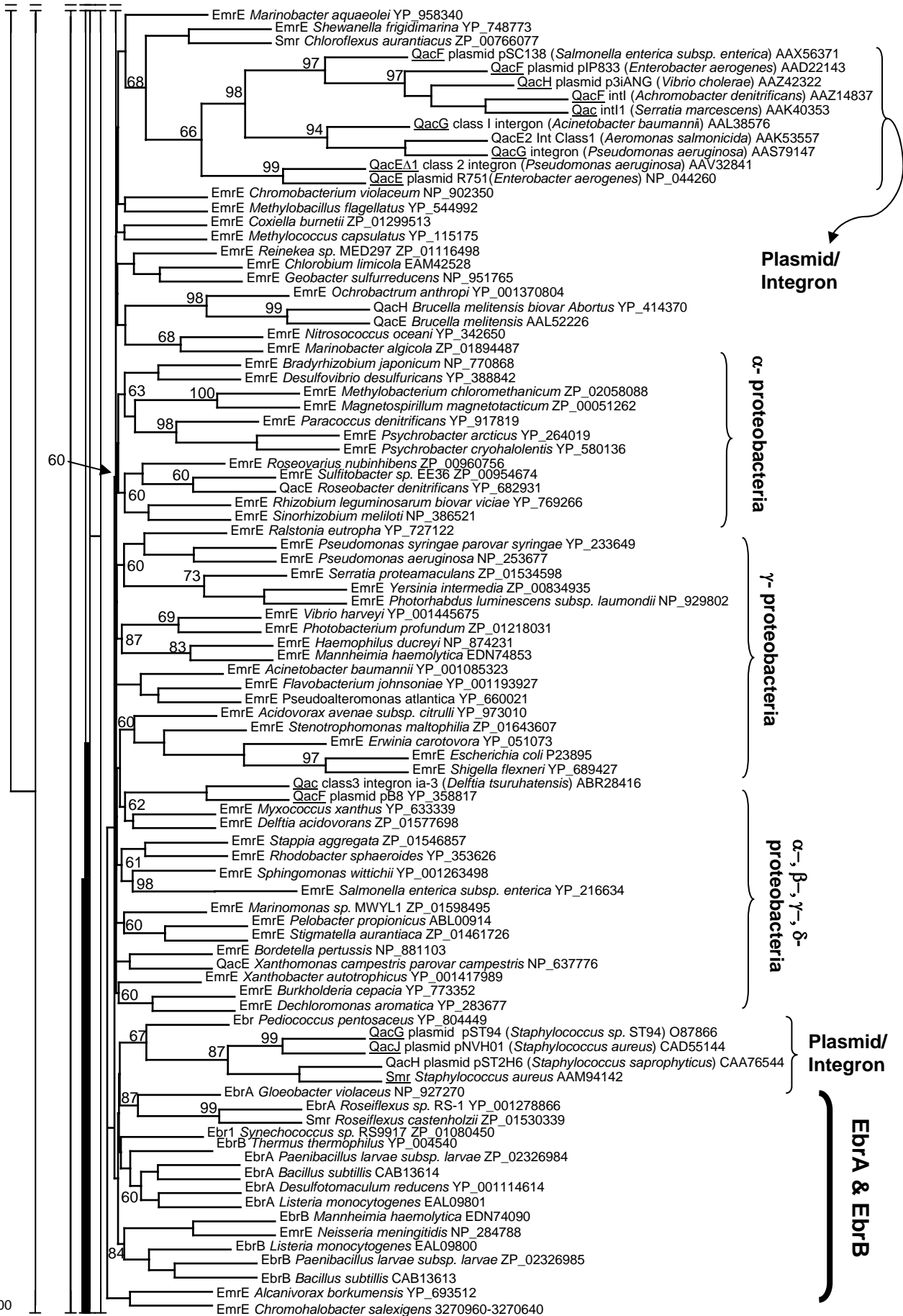

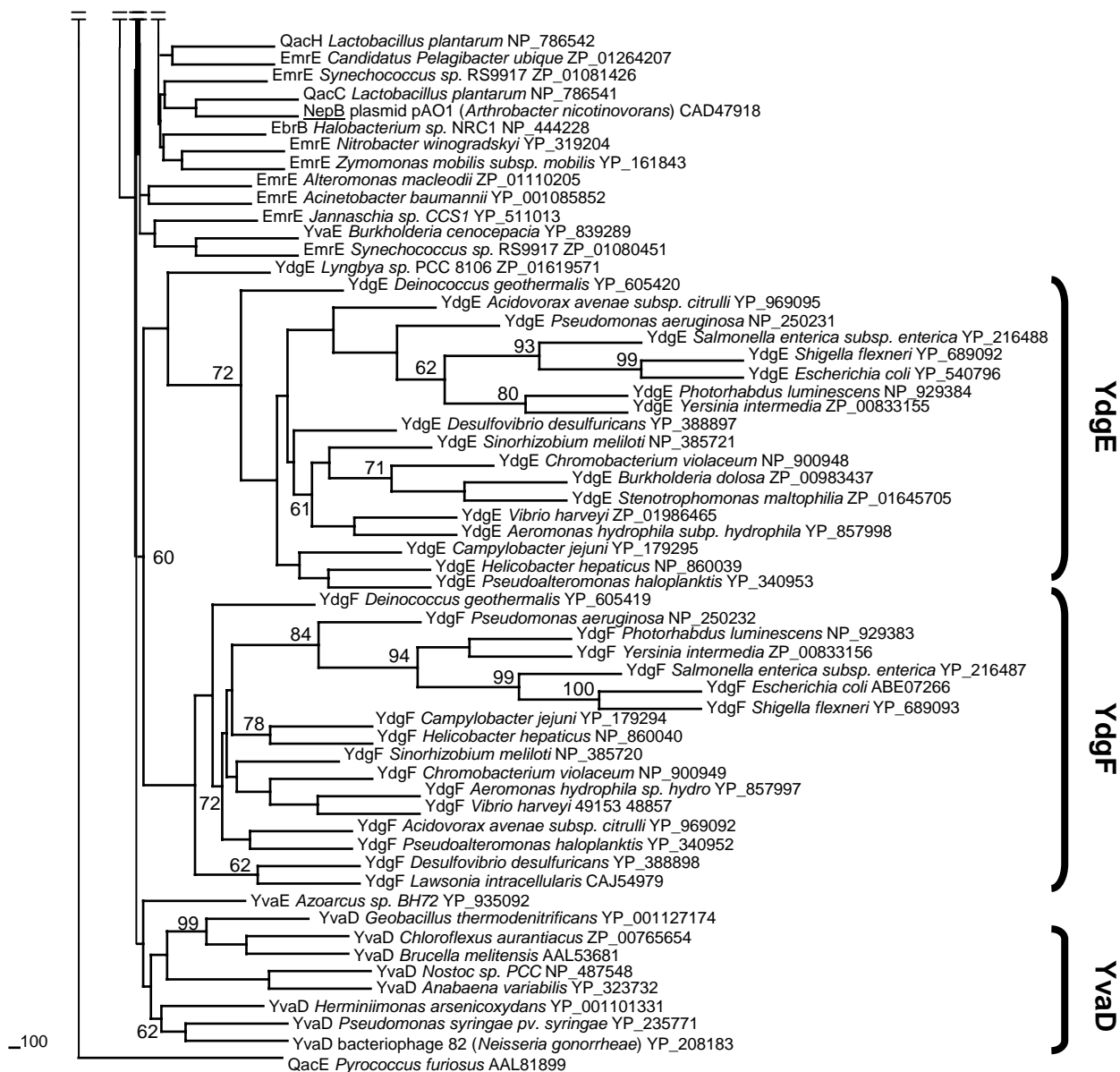

Supplement: Additional file 1 — A phylogenetic tree of the SMR protein family. The rooted phylogenetic tree is based on Neighbour-Joining analysis of 338 SMR protein sequences. The Archaeal Archaeoglobus QacE sequence served as an outgroup for this analysis. In some cases, individual PSMR sequences are highlighted by parenthesis and listed around branches to indicate important groupings. One thousand bootstrap replicates were performed and confidence values (by percentage) are listed beside their respective nodes above 59%. Plasmid and integron encoded SMR proteins are underlined. SMR sequence accession numbers are indicated adjacent to its genus and species name. [file 1471-2148-9-140-S1.pdf]
