# Supplementary material for: Diversity and evolution of the small multidrug resistance protein family
Source: BMC Evol Biol. 2009 Jun 23;9:140. doi: 10.1186/1471-2148-9-140 (PMC2716321; doi:10.1186/1471-2148-9-140)
Supplement: Additional file 2 — Summary of synonymous to non-synonymous nucleotide substitution patterns within PSMR subclass members. [file 1471-2148-9-140-S2.doc]

**Table 4.** Summary of synonymous to non- synonymous nucleotide substitution patterns within PSMR subclass members.

| **SMR 1** | **SMR 2** | **Sd** | **Nd** | **S** | **N** | **pS** | **pN** | **dS** | **dN** | **dS/dN** |
| --- | --- | --- | --- | --- | --- | --- | --- | --- | --- | --- |
| ***yvaE*** | **mean** | 56.24 | 108.2 | 77.11 | 222.89 | 0.73 | 0.49 | 2.21 | 0.84 | **3.16** |
| Bsu- *yvaE* | Mma- *yvaE* | 42.75 | 172.25 | 72.33 | 227.67 | 0.59 | 0.76 | 1.16 | NA | NA |
|  | Aba- *yvaE* | 64.12 | 80.88 | 76.5 | 223.5 | 0.84 | 0.36 | NA | 0.49 | NA |
|  | Cte- *yvaE* | 67.12 | 75.88 | 75.33 | 224.67 | 0.89 | 0.34 | NA | 0.45 | NA |
|  | Dha- *yvaE* | 63.62 | 72.38 | 75.67 | 224.33 | 0.84 | 0.32 | NA | 0.42 | NA |
|  | Bce- *yvaE* | 65.12 | 100.88 | 76.33 | 223.67 | 0.85 | 0.45 | NA | 0.69 | NA |
|  | Dsu- *yvaE* | 54.25 | 83.75 | 73 | 227 | 0.74 | 0.37 | 3.52 | 0.51 | 6.94 |
|  | Pae- *yvaE* | 58.88 | 98.12 | 77.83 | 222.17 | 0.76 | 0.44 | NA | 0.67 | NA |
|  | I- *qacE* | 51.12 | 90.88 | 75 | 225 | 0.68 | 0.4 | 1.8 | 0.58 | 3.1 |
|  | PI- *qacEΔ1* | 52 | 98 | 75.5 | 224.5 | 0.69 | 0.44 | 1.88 | 0.65 | 2.87 |
|  | P- *qacF* | 61.62 | 100.38 | 74.67 | 225.33 | 0.83 | 0.45 | NA | 0.68 | NA |
|  | P- *qacG* | 52.62 | 90.38 | 74.5 | 225.5 | 0.71 | 0.4 | 2.13 | 0.57 | 3.72 |
|  | P- *qacH* | 53.25 | 96.75 | 70.33 | 229.67 | 0.76 | 0.42 | NA | 0.62 | NA |
|  | P- *qacJ* | 49.12 | 92.88 | 71.83 | 228.17 | 0.68 | 0.41 | 1.82 | 0.59 | 3.1 |
|  | I- *sugE* | 54.38 | 117.62 | 76.33 | 223.67 | 0.71 | 0.53 | 2.24 | 0.91 | 2.48 |
|  | P-Cfr- *sugE* | 51.12 | 107.88 | 76.67 | 223.33 | 0.67 | 0.48 | 1.65 | 0.77 | 2.13 |
|  | P- *nepA* | 64.12 | 92.88 | 76.5 | 223.5 | 0.84 | 0.42 | NA | 0.61 | NA |
|  | P- *nepB* | 62.5 | 105.5 | 77 | 223 | 0.81 | 0.47 | NA | 0.75 | NA |
| ***yvaD*** | **mean** | 52.59 | 135.3 | 75.68 | 221.32 | 0.7 | 0.61 | 1.8 | 1.46 | **1.69** |
| Bsu- *yvaD* | Bsu- *yvaE* | 39.88 | 165.12 | 68.67 | 228.33 | 0.58 | 0.72 | 1.12 | 2.5 | 0.45 |
|  | Bth- *yvaD* | 39.62 | 120.38 | 61.67 | 235.33 | 0.64 | 0.51 | 1.46 | 0.86 | 1.7 |
|  | Cau- *yvaD* | 47.38 | 170.62 | 71.67 | 225.33 | 0.66 | 0.76 | 1.6 | NA | NA |
|  | Avi- *yvaD* | 44.75 | 171.25 | 70.5 | 226.5 | 0.63 | 0.76 | 1.4 | NA | NA |
|  | Pub- *yvaD* | 45 | 166 | 69.17 | 227.83 | 0.65 | 0.73 | 1.52 | 2.67 | 0.57 |
|  | Rme- *yvaD* | 47.38 | 153.62 | 74.5 | 222.5 | 0.64 | 0.69 | 1.41 | 1.9 | 0.74 |
|  | Pse- *yvaD* | 42.75 | 166.25 | 74.5 | 222.5 | 0.57 | 0.75 | 1.09 | 4.19 | 0.26 |
|  | Ade- *yvaD* | 55.38 | 164.62 | 76.17 | 220.83 | 0.73 | 0.75 | 2.61 | 3.83 | 0.68 |
|  | I- *qacE* | 44.12 | 164.88 | 71 | 226 | 0.62 | 0.73 | 1.32 | 2.7 | 0.49 |
|  | PI- *qacEΔ1* | 46.25 | 165.75 | 71.5 | 225.5 | 0.65 | 0.74 | 1.49 | 2.94 | 0.51 |
|  | P- *qacF* | 47.62 | 171.38 | 70.67 | 226.33 | 0.67 | 0.76 | 1.72 | NA | NA |
|  | P- *qacG* | 47.88 | 165.12 | 70.5 | 226.5 | 0.68 | 0.73 | 1.77 | 2.68 | 0.66 |
|  | P- *qacH* | 47.38 | 158.62 | 66.33 | 230.67 | 0.71 | 0.69 | 2.28 | 1.87 | 1.22 |
|  | P- *qacJ* | 44 | 157 | 67.83 | 229.17 | 0.65 | 0.69 | 1.5 | 1.84 | 0.82 |
|  | I- *sugE* | 41 | 163 | 72.33 | 224.67 | 0.57 | 0.73 | 1.06 | 2.57 | 0.41 |
|  | P-Cfr- *sugE* | 46 | 163 | 72.67 | 224.33 | 0.63 | 0.73 | 1.39 | 2.6 | 0.54 |
|  | P- *nepA* | 48.12 | 170.88 | 72.5 | 224.5 | 0.66 | 0.76 | 1.62 | NA | NA |
|  | P- *nepB* | 45.5 | 165.5 | 73 | 224 | 0.62 | 0.74 | 1.33 | 3.16 | 0.42 |
| ***ykkC*/ *ykkD*** | **mean** | 51.94 | 133.28 | 75.22 | 224.78 | 0.69 | 0.59 | 1.86 | 1.34 | **1.71** |
| Bsu- *ykkC* | Bsu- *ykkD* | 54.38 | 114.62 | 73.33 | 226.67 | 0.74 | 0.51 | 3.36 | 0.84 | 3.99 |
|  | Lbr- *ykkC* | 48.5 | 77.5 | 75.83 | 224.17 | 0.64 | 0.35 | 1.44 | 0.46 | 3.1 |
|  | Dsa- *ykkC* | 60 | 67 | 74.67 | 225.33 | 0.8 | 0.3 | NA | 0.38 | NA |
|  | Dsa- *ykkD* | 47.62 | 110.38 | 74 | 226 | 0.64 | 0.49 | 1.46 | 0.79 | 1.85 |
|  | Csa- *ykkD* | 56.38 | 160.62 | 80.33 | 219.67 | 0.7 | 0.73 | 2.06 | 2.77 | 0.74 |
|  | Csa- *ykkC* | 59.38 | 93.62 | 79.33 | 220.67 | 0.75 | 0.42 | 4.62 | 0.63 | 7.39 |
|  | Cje- *ykkC* | 57.5 | 102.5 | 70 | 230 | 0.82 | 0.45 | NA | 0.68 | NA |
|  | I- *qacE* | 56.12 | 144.88 | 76.5 | 223.5 | 0.73 | 0.65 | 2.87 | 1.5 | 1.92 |
|  | PI- *qacEΔ1* | 55.12 | 147.88 | 77 | 223 | 0.72 | 0.66 | 2.32 | 1.62 | 1.43 |
|  | P- *qacF* | 54.12 | 151.88 | 76.17 | 223.83 | 0.71 | 0.68 | 2.21 | 1.76 | 1.25 |
|  | P- *qacG* | 48 | 152 | 76 | 224 | 0.63 | 0.68 | 1.38 | 1.76 | 0.78 |
|  | P- *qacH* | 48.88 | 146.12 | 71.83 | 228.17 | 0.68 | 0.64 | 1.78 | 1.44 | 1.24 |
|  | P- *qacJ* | 47.5 | 150.5 | 73.33 | 226.67 | 0.65 | 0.66 | 1.49 | 1.62 | 0.92 |
|  | I- *sugE* | 54.88 | 143.12 | 77.83 | 222.17 | 0.71 | 0.64 | 2.11 | 1.47 | 1.44 |
|  | P-Cfr- *sugE* | 56.12 | 140.88 | 78.17 | 221.83 | 0.72 | 0.64 | 2.37 | 1.41 | 1.68 |
|  | P- *nepA* | 54.5 | 154.5 | 78 | 222 | 0.7 | 0.7 | 2.01 | 1.97 | 1.02 |
|  | P- *nepB* | 55.38 | 152.62 | 78.5 | 221.5 | 0.71 | 0.69 | 2.12 | 1.88 | 1.12 |
| Bsu- *ykkD* | Lbr- *ykkC* | 59 | 125 | 73.5 | 226.5 | 0.8 | 0.55 | NA | 1 | NA |
|  | Lbr- *ykkD* | 42.38 | 91.62 | 70.83 | 229.17 | 0.6 | 0.4 | 1.2 | 0.57 | 2.1 |
|  | Dsa- *ykkC* | 46.75 | 129.25 | 72.33 | 227.67 | 0.65 | 0.57 | 1.48 | 1.06 | 1.4 |
|  | Dsa- *ykkD* | 54.38 | 75.62 | 71.67 | 228.33 | 0.76 | 0.33 | NA | 0.44 | NA |
|  | Csa- *ykkC* | 49 | 136 | 77 | 223 | 0.64 | 0.61 | 1.42 | 1.26 | 1.12 |
|  | Csa- *ykkD* | 55.25 | 170.75 | 78 | 222 | 0.71 | 0.77 | 2.17 | NA | NA |
|  | Cje- *ykkD* | 46.38 | 143.62 | 66.83 | 233.17 | 0.69 | 0.62 | 1.94 | 1.29 | 1.51 |
|  | I- *qacE* | 48.5 | 164.5 | 74.17 | 225.83 | 0.65 | 0.73 | 1.54 | 2.66 | 0.58 |
|  | PI- *qacEΔ1* | 48.25 | 168.75 | 74.67 | 225.33 | 0.65 | 0.75 | 1.48 | 4.89 | 0.3 |
|  | P- *qacF* | 42.88 | 148.12 | 73.83 | 226.17 | 0.58 | 0.65 | 1.12 | 1.55 | 0.72 |
|  | P- *qacG* | 50.38 | 160.62 | 73.67 | 226.33 | 0.68 | 0.71 | 1.82 | 2.19 | 0.83 |
|  | P- *qacH* | 51.75 | 154.25 | 69.5 | 230.5 | 0.74 | 0.67 | 3.7 | 1.67 | 2.21 |
|  | P- *qacJ* | 51.75 | 155.25 | 71 | 229 | 0.73 | 0.68 | 2.68 | 1.76 | 1.52 |
|  | I- *sugE* | 45.12 | 156.88 | 75.5 | 224.5 | 0.6 | 0.7 | 1.2 | 2.01 | 0.59 |
|  | P-Cfr- *sugE* | 47.75 | 148.25 | 75.83 | 224.17 | 0.63 | 0.66 | 1.37 | 1.6 | 0.86 |
|  | P- *nepA* | 46.38 | 143.62 | 66.83 | 233.17 | 0.69 | 0.62 | 1.94 | 1.29 | 1.51 |
|  | P- *nepB* | 49.75 | 165.25 | 75.67 | 224.33 | 0.66 | 0.74 | 1.57 | 3.02 | 0.52 |
| ***yvdR*/ *yvdS*** | **mean** | 53.02 | 111.23 | 74.45 | 216.55 | 0.71 | 0.51 | 1.92 | 1.01 | **2.04** |
| Bsu- *yvdR* | Bsu- *yvdS* | 48 | 133 | 71.17 | 219.83 | 0.67 | 0.61 | 1.72 | 1.23 | 1.4 |
|  | Oih- *yvdR* | 55.62 | 93.38 | 72 | 219 | 0.77 | 0.43 | NA | 0.63 | NA |
|  | Oih- *yvdS* | 55.5 | 116.5 | 74.33 | 216.67 | 0.75 | 0.54 | 4.06 | 0.95 | 4.28 |
|  | I- *qacE* | 62.88 | 94.12 | 73.67 | 217.33 | 0.85 | 0.43 | NA | 0.65 | NA |
|  | PI- *qacEΔ1* | 58.88 | 97.12 | 74.17 | 216.83 | 0.79 | 0.45 | NA | 0.68 | NA |
|  | P- *qacF* | 57.25 | 96.75 | 72.83 | 218.17 | 0.79 | 0.44 | NA | 0.67 | NA |
|  | P- *qacG* | 56.62 | 97.38 | 73 | 218 | 0.78 | 0.45 | NA | 0.68 | NA |
|  | P- *qacH* | 57.5 | 114.5 | 69 | 222 | 0.83 | 0.52 | NA | 0.87 | NA |
|  | P- *qacJ* | 55.5 | 102.5 | 70.5 | 220.5 | 0.79 | 0.46 | NA | 0.73 | NA |
|  | I- *sugE* | 50 | 158 | 74.5 | 216.5 | 0.67 | 0.73 | 1.69 | 2.71 | 0.62 |
|  | P-Cfr- *sugE* | 47.75 | 91.25 | 75 | 216 | 0.64 | 0.42 | 1.42 | 0.62 | 2.28 |
|  | P- *nepA* | 49.62 | 110.38 | 75.33 | 215.67 | 0.66 | 0.51 | 1.58 | 0.86 | 1.84 |
|  | P- *nepB* | 54.88 | 113.12 | 75.67 | 215.33 | 0.73 | 0.53 | 2.56 | 0.9 | 2.83 |
| Bsu- *yvdS* | Oih- *yvdR* | 52.75 | 111.25 | 71.5 | 219.5 | 0.74 | 0.51 | 3.09 | 0.84 | 3.65 |
|  | Oih- *yvdS* | 43 | 97 | 73.83 | 217.17 | 0.58 | 0.45 | 1.12 | 0.68 | 1.66 |
|  | I- *qacE* | 46.12 | 128.88 | 73.17 | 217.83 | 0.63 | 0.59 | 1.38 | 1.17 | 1.18 |
|  | PI- *qacEΔ1* | 49.25 | 130.75 | 73.67 | 217.33 | 0.67 | 0.6 | 1.67 | 1.22 | 1.37 |
|  | P- *qacF* | 50.12 | 132.88 | 72.33 | 218.67 | 0.69 | 0.61 | 1.93 | 1.25 | 1.55 |
|  | P- *qacG* | 46 | 128 | 72.5 | 218.5 | 0.63 | 0.59 | 1.4 | 1.14 | 1.23 |
|  | P- *qacH* | 46.25 | 138.75 | 68.5 | 222.5 | 0.68 | 0.62 | 1.73 | 1.34 | 1.29 |
|  | P- *qacJ* | 47.25 | 133.75 | 70 | 221 | 0.68 | 0.61 | 1.73 | 1.23 | 1.4 |
|  | I- *sugE* | 56.88 | 128.12 | 74 | 217 | 0.77 | 0.59 | NA | 1.16 | NA |
|  | P-Cfr- *sugE* | 50 | 109 | 74.5 | 216.5 | 0.67 | 0.5 | 1.69 | 0.83 | 2.02 |
|  | P- *nepA* | 50.88 | 138.12 | 74.83 | 216.17 | 0.68 | 0.64 | 1.78 | 1.43 | 1.24 |
|  | P- *nepB* | 48.62 | 136.38 | 75.17 | 215.83 | 0.65 | 0.63 | 1.49 | 1.39 | 1.07 |
| ***ebrA*/ *ebrB*** | **mean** | 55.61 | 116.63 | 77.39 | 222.61 | 0.72 | 0.52 | 2.09 | 1.09 | **2.48** |
| Bsu- *ebrA* | Mac- *ebrA* | 54.5 | 100.5 | 74.5 | 225.5 | 0.73 | 0.45 | 2.78 | 0.68 | 4.11 |
|  | Lpl- *eacC* | 58.12 | 100.88 | 73.83 | 226.17 | 0.79 | 0.45 | NA | 0.68 | NA |
|  | Lpl- *qacH* | 60.25 | 111.75 | 74.83 | 225.17 | 0.81 | 0.5 | NA | 0.81 | NA |
|  | Bsu- *ebrB* | 59.38 | 93.62 | 75 | 225 | 0.79 | 0.42 | NA | 0.61 | NA |
|  | Mva- *ebr2* | 55.88 | 143.12 | 79.17 | 220.83 | 0.71 | 0.65 | 2.12 | 1.5 | 1.42 |
|  | Mva- *ebr1* | 55.62 | 153.38 | 80.33 | 219.67 | 0.69 | 0.7 | 1.93 | 2 | 0.96 |
|  | Ssp- *ebr1* | 55.12 | 108.88 | 75 | 225 | 0.73 | 0.48 | 2.93 | 0.78 | 3.78 |
|  | Ssp- *ebr2* | 59.12 | 101.88 | 75.33 | 224.67 | 0.78 | 0.45 | NA | 0.7 | NA |
|  | I- *qacE* | 58.38 | 95.62 | 75.5 | 224.5 | 0.77 | 0.43 | NA | 0.63 | NA |
|  | PI- *qacEΔ1* | 58.25 | 100.75 | 76 | 224 | 0.77 | 0.45 | NA | 0.69 | NA |
|  | P- *qacF* | 54.5 | 87.5 | 75.17 | 224.83 | 0.73 | 0.39 | 2.55 | 0.55 | 4.65 |
|  | P- *qacG* | 54.88 | 98.12 | 75 | 225 | 0.73 | 0.44 | 2.78 | 0.65 | 4.26 |
|  | P- *qacH* | 52.38 | 96.62 | 70.83 | 229.17 | 0.74 | 0.42 | 3.2 | 0.62 | 5.16 |
|  | P- *qacJ* | 57.5 | 96.5 | 72.33 | 227.67 | 0.79 | 0.42 | NA | 0.62 | NA |
|  | I- *sugE* | 48.62 | 157.38 | 76.83 | 223.17 | 0.63 | 0.71 | 1.39 | 2.11 | 0.66 |
|  | P-Cfr- *sugE* | 55.25 | 121.75 | 77.17 | 222.83 | 0.72 | 0.55 | 2.32 | 0.98 | 2.37 |
|  | P- *nepA* | 63.38 | 107.62 | 77 | 223 | 0.82 | 0.48 | NA | 0.77 | NA |
|  | P- *nepB* | 55.12 | 107.88 | 77.5 | 222.5 | 0.71 | 0.48 | 2.22 | 0.78 | 2.85 |
| Bsu- *ebrB* | Mac- *ebrA* | 57 | 94 | 75.83 | 224.17 | 0.75 | 0.42 | NA | 0.61 | NA |
|  | Lpl- *qacC* | 53.62 | 105.38 | 75.17 | 224.83 | 0.71 | 0.47 | 2.27 | 0.74 | 3.08 |
|  | Lpl- *qacH* | 49.62 | 112.38 | 76.17 | 223.83 | 0.65 | 0.5 | 1.52 | 0.83 | 1.83 |
|  | Mva- *ebr2* | 63 | 137 | 80.5 | 219.5 | 0.78 | 0.62 | NA | 1.34 | NA |
|  | Mva- *ebr1* | 59 | 150 | 81.67 | 218.33 | 0.72 | 0.69 | 2.48 | 1.86 | 1.33 |
|  | Ssp- *ebr1* | 60 | 109 | 76.33 | 223.67 | 0.79 | 0.49 | NA | 0.79 | NA |
|  | Ssp- *ebr2* | 59.62 | 104.38 | 76.67 | 223.33 | 0.78 | 0.47 | NA | 0.73 | NA |
|  | I- *qacE* | 59.88 | 92.12 | 76.83 | 223.17 | 0.78 | 0.41 | NA | 0.6 | NA |
|  | PI- *qacEΔ1* | 56.88 | 99.12 | 77.33 | 222.67 | 0.74 | 0.45 | 2.96 | 0.68 | 4.38 |
|  | P- *qacF* | 64.12 | 97.88 | 76.5 | 223.5 | 0.84 | 0.44 | NA | 0.66 | NA |
|  | P- *qacG* | 54.38 | 97.62 | 76.33 | 223.67 | 0.71 | 0.44 | 2.24 | 0.65 | 3.43 |
|  | P- *qacH* | 54 | 97 | 72.17 | 227.83 | 0.75 | 0.43 | 4.55 | 0.63 | 7.24 |
|  | P- *qacJ* | 53 | 92 | 73.67 | 226.33 | 0.72 | 0.41 | 2.4 | 0.59 | 4.1 |
|  | I- *sugE* | 54.62 | 156.38 | 78.17 | 221.83 | 0.7 | 0.7 | 2.01 | 2.11 | 0.95 |
|  | P-Cfr- *sugE* | 55.75 | 104.25 | 78.5 | 221.5 | 0.71 | 0.47 | 2.2 | 0.74 | 2.97 |
|  | P- *nepA* | 55.75 | 96.25 | 78.33 | 221.67 | 0.71 | 0.43 | 2.23 | 0.65 | 3.44 |
|  | P- *nepB* | 62.75 | 97.25 | 78.83 | 221.17 | 0.8 | 0.44 | NA | 0.66 | NA |
| ***ydgE*/ *ydgF*** | **mean** | 53.19 | 131 | 77.48 | 222.52 | 0.69 | 0.59 | 1.77 | 1.37 | **1.84** |
| Eco- *ydgE* | Eco- *ydgF* | 42.5 | 170.5 | 73.67 | 226.33 | 0.58 | 0.75 | 1.1 | NA | NA |
|  | Sme- *ydgE* | 51.12 | 156.88 | 79.17 | 220.83 | 0.65 | 0.71 | 1.48 | 2.21 | 0.67 |
|  | Sme- *ydgF* | 45.25 | 166.75 | 78.83 | 221.17 | 0.57 | 0.75 | 1.09 | NA | NA |
|  | Cvi- *ydgE* | 48 | 163 | 79.33 | 220.67 | 0.61 | 0.74 | 1.23 | 3.14 | 0.39 |
|  | Cvi- *ydgF* | 47.62 | 157.38 | 80.5 | 219.5 | 0.59 | 0.72 | 1.17 | 2.34 | 0.5 |
|  | Dvu- *ydgE* | 49.5 | 151.5 | 81 | 219 | 0.61 | 0.69 | 1.26 | 1.92 | 0.66 |
|  | Cje- *ydgE* | 40.88 | 177.12 | 72.17 | 227.83 | 0.57 | 0.78 | 1.06 | NA | NA |
|  | I- *qacE* | 47.75 | 162.25 | 76.33 | 223.67 | 0.63 | 0.73 | 1.35 | 2.56 | 0.53 |
|  | PI- *qacEΔ1* | 47 | 170 | 76.83 | 223.17 | 0.61 | 0.76 | 1.27 | NA | NA |
|  | P- *qacF* | 50.75 | 164.25 | 76 | 224 | 0.67 | 0.73 | 1.66 | 2.85 | 0.58 |
|  | P- *qacG* | 51.12 | 163.88 | 75.83 | 224.17 | 0.67 | 0.73 | 1.72 | 2.76 | 0.62 |
|  | P- *qacH* | 48.75 | 168.25 | 71.67 | 228.33 | 0.68 | 0.74 | 1.78 | 3.03 | 0.59 |
|  | P- *qacJ* | 47.5 | 163.5 | 73.17 | 226.83 | 0.65 | 0.72 | 1.51 | 2.43 | 0.62 |
|  | I- *sugE* | 51.75 | 164.25 | 77.67 | 222.33 | 0.67 | 0.74 | 1.64 | 3.15 | 0.52 |
|  | P-Cfr- *sugE* | 51.75 | 166.25 | 78 | 222 | 0.66 | 0.75 | 1.62 | 4.88 | 0.33 |
|  | P- *nepA* | 50.25 | 166.75 | 77.83 | 222.17 | 0.65 | 0.75 | 1.48 | NA | NA |
|  | P- *nepB* | 43.75 | 157.25 | 78.33 | 221.67 | 0.56 | 0.71 | 1.02 | 2.19 | 0.47 |
| Eco- *ydgF* | Sme- *ydgE* | 48.75 | 166.25 | 77.5 | 222.5 | 0.63 | 0.75 | 1.37 | 4.19 | 0.33 |
|  | Sme- *ydgF* | 65.75 | 87.25 | 77.17 | 222.83 | 0.85 | 0.39 | NA | 0.55 | NA |
|  | Cvi- *ydgE* | 46.88 | 162.12 | 77.67 | 222.33 | 0.6 | 0.73 | 1.22 | 2.69 | 0.46 |
|  | Cvi- *ydgF* | 50 | 95 | 78.83 | 221.17 | 0.63 | 0.43 | 1.4 | 0.64 | 2.2 |
|  | Dvu- *ydgF* | 50.25 | 101.75 | 75 | 225 | 0.67 | 0.45 | 1.68 | 0.69 | 2.42 |
|  | Cje- *ydgF* | 44.75 | 94.25 | 68.17 | 231.83 | 0.66 | 0.41 | 1.56 | 0.59 | 2.67 |
|  | I- *qacE* | 48.88 | 119.12 | 74.67 | 225.33 | 0.65 | 0.53 | 1.55 | 0.92 | 1.69 |
|  | PI- *qacEΔ1* | 47.75 | 120.25 | 75.17 | 224.83 | 0.64 | 0.53 | 1.41 | 0.94 | 1.5 |
|  | P- *qacF* | 60.62 | 114.38 | 74.33 | 225.67 | 0.82 | 0.51 | NA | 0.84 | NA |
|  | P- *qacG* | 55.38 | 115.62 | 74.17 | 225.83 | 0.75 | 0.51 | 4.05 | 0.86 | 4.71 |
|  | P- *qacH* | 50.5 | 126.5 | 70 | 230 | 0.72 | 0.55 | 2.45 | 0.99 | 2.47 |
|  | P- *qacJ* | 51 | 120 | 71.5 | 228.5 | 0.71 | 0.53 | 2.26 | 0.9 | 2.5 |
|  | I- *sugE* | 47.38 | 152.62 | 76 | 224 | 0.62 | 0.68 | 1.33 | 1.79 | 0.74 |
|  | P-Cfr- *sugE* | 45.5 | 132.5 | 76.33 | 223.67 | 0.6 | 0.59 | 1.19 | 1.17 | 1.02 |
|  | P- *nepA* | 56.62 | 121.38 | 76.17 | 223.83 | 0.74 | 0.54 | 3.55 | 0.96 | 3.69 |
|  | P- *nepB* | 60.12 | 111.88 | 76.67 | 223.33 | 0.78 | 0.5 | NA | 0.83 | NA |
|  |  |  |  |  |  |  |  |  |  |  |

Refer to footnotes on Table 3 for definitions of the following abbreviations: Sd, Nd, S, N, pS, pN, dS, and dN.

Species are listed on the table according to their three letter species abbreviation followed by the SMR subclass name: **Aba** *Acidobacteria bacterium*; **Ade** *Anaeromyxobacter dehalogenans*; **Avi** *Anabaena variabilis*; **Bce** *Burkholderia cenocepacia*; **Bsu** *Bacillus subtilis*; **Bth** *Bacillus thuringiensis*; **Cau** *Chloroflexus aurantiacus*; **Cfr** *Citrobacter freundii*; **Cje** *Campylobacter jejuni*; **Csa** *Chromohalobacter* *salexigens*; **Cte** *Chlorobium tepidum*; **Cvi** *Chromobacterium violaceum*; **Dha** *Desulfitobacterium hafniense*; **Dsu** *Desulfovibrio vulgaris*; **Eco** *Escherichia coli*; **Lbr** *Lactobacillus brevis*; **Mac** *Methanosarcina acetivorans*; **Mma** *Methanosarcina mazei*; **Mva** *Mycobacterium vanbaalenii*; **Oih** *Oceanobacillus iheyensis*; **Pae** *Pseudomonas aeruginosa*; **Psy** *Pseudomonas syringae*; **Pub** *Pelagibacter ubique*; **Sme** *Sinorhizobium meliloti*; **Ssp** *Synechococcus sp. RS9917*; **Rme** *Ralstonia metallidurans*; **P** plasmid encoded; **I** integron encoded; **PI** plasmid/ integron encoded; **NA** data not available
