# Supplementary material for: Diversity and evolution of the small multidrug resistance protein family
Source: BMC Evol Biol. 2009 Jun 23;9:140. doi: 10.1186/1471-2148-9-140 (PMC2716321; doi:10.1186/1471-2148-9-140)
Supplement: Additional file 3 — An alignment of 338 SMR protein sequences identified from BLAST surveys of completed Archaeal and Bacterial genomes. The 338 SMR protein sequence alignment was truncated from a larger alignment of 685 SMR sequences that was generated using a manually edited ClustalW alignment. It is important to note that this alignment may contain truncated versions of some SMR sequences. [file 1471-2148-9-140-S3.pdf]

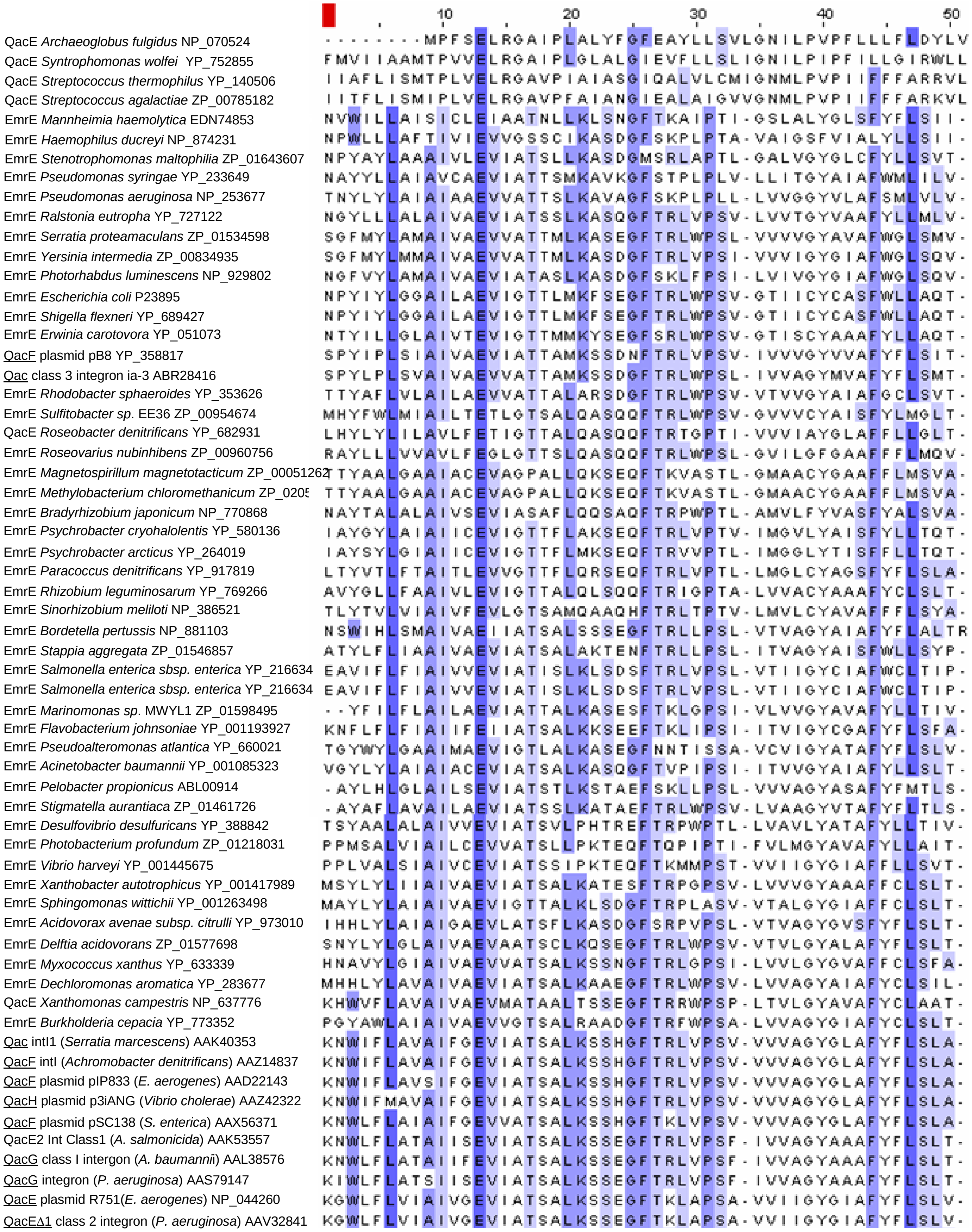
10
20
30
40
50
QacE Archaeoglobus fulgidus NP_070524
QacE Syntrophomonas wolfei YP_752855
QacE Streptococcus thermophilus YP_140506
QacE Streptococcus agalactiae ZP_00785182
EmrE Mannheimia haemolytica EDN74853
EmrE Haemophilus ducreyi NP_874231
EmrE Stenotrophomonas maltophilia ZP_01643607
EmrE Pseudomonas syringae YP_233649
EmrE Pseudomonas aeruginosa NP_253677
EmrE Ralstonia eutropha YP_727122
EmrE Serratia proteamaculans ZP_01534598
EmrE Yersinia intermedia ZP_00834935
EmrE Photorhabdus luminescens NP_929802
EmrE Escherichia coli P23895
EmrE Shigella flexneri YP_689427
EmrE Erwinia carotovora YP_051073
QacF plasmid pB8 YP_358817
Qac class 3 integron ia-3 ABR28416
EmrE Rhodobacter sphaeroides YP_353626
EmrE Sulfitobacter sp. EE36 ZP_00954674
QacE Roseobacter denitrificans YP_682931
EmrE Roseovarius nubinhibens ZP_00960756
EmrE Magnetospirillum magnetotacticum ZP_00051262
EmrE Methylobacterium chloromethanicum ZP_0205
EmrE Bradyrhizobium japonicum NP_770868
EmrE Psychrobacter cryohalolentis YP_580136
EmrE Psychrobacter arcticus YP_264019
EmrE Paracoccus denitrificans YP_917819
EmrE Rhizobium leguminosarum YP_769266
EmrE Sinorhizobium meliloti NP_386521
EmrE Bordetella pertussis NP_881103
EmrE Stappia aggregata ZP_01546857
EmrE Salmonella enterica sbsp. enterica YP_216634
EmrE Salmonella enterica sbsp. enterica YP_216634
EmrE Marinomonas sp. MWYL1 ZP_01598495
EmrE Flavobacterium johnsoniae YP_001193927
EmrE Pseudoalteromonas atlantica YP_660021
EmrE Acinetobacter baumannii YP_001085323
EmrE Pelobacter propionicus ABL00914
EmrE Stigmatella aurantiaca ZP_01461726
EmrE Desulfovibrio desulfuricans YP_388842
EmrE Photobacterium profundum ZP_01218031
EmrE Vibrio harveyi YP_001445675
EmrE Xanthobacter autotrophicus YP_001417989
EmrE Sphingomonas wittichii YP_001263498
EmrE Acidovorax avenae subsp. citrulli YP_973010
EmrE Delftia acidovorans ZP_01577698
EmrE Myxococcus xanthus YP_633339
EmrE Dechloromonas aromatica YP_283677
QacE Xanthomonas campestris NP_637776
EmrE Burkholderia cepacia YP_773352
Qac intI1 (Serratia marcescens) AAK40353
QacF intI (Achromobacter denitrificans) AAZ14837
QacF plasmid pIP833 (E. aerogenes) AAD22143
QacH plasmid p3iANG (Vibrio cholerae) AAZ42322
QacF plasmid pSC138 (S. enterica) AAX56371
QacE2 Int Class1 (A. salmonicida) AAK53557
QacG class I intergon (A. baumannii) AAL38576
QacG integron (P. aeruginosa) AAS79147
QacE plasmid R751(E. aerogenes) NP_044260
QacEΔ1 class 2 integron (P. aeruginosa) AAV32841

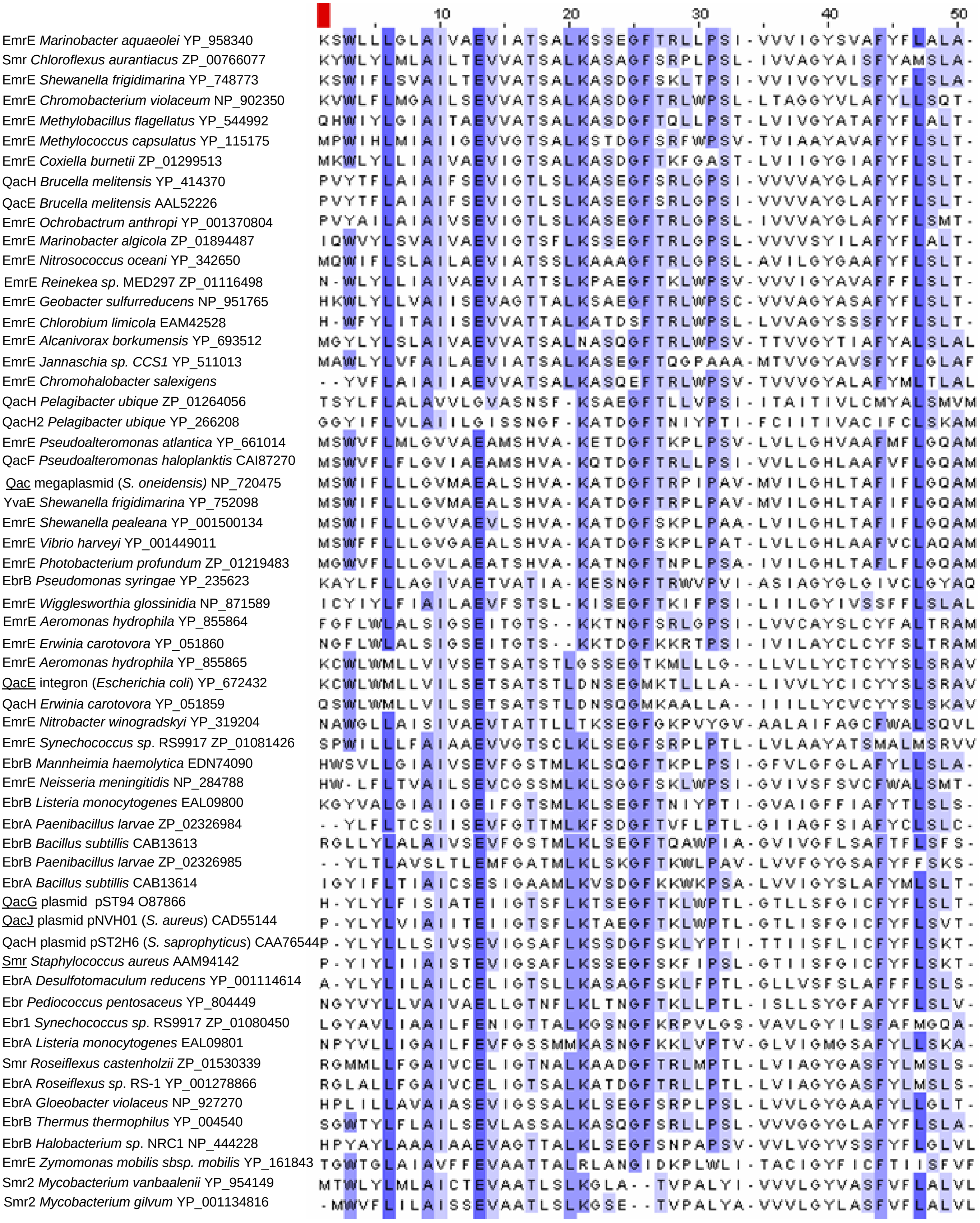
10 20 30 40 50
EmrE Marinobacter aquaeolei YP_958340 KSWLLLGLAIVAEVIATSALKSSEGFTRLLPSI-VVVIGYSVAFYFLALA-
Smr Chloroflexus aurantiacus ZP_00766077 KYWLYLMLAILTEVVATSALKASAGFSRPLPSL-IVVAGYAISFYAMSLA-
EmrE Shewanella frigidimarina YP_748773 KSWIFLSVAILTEVVATSALKASDGFSKLTPSI-VVIVGYVLSFYFLSLA-
EmrE Chromobacterium violaceum NP_902350 KVWLFLMGAILSEVVATSALKASDGFTRLWPSL-LTAGGYVLAFYLLSQT-
EmrE Methylobacillus flagellatus YP_544992 QHWIYLGIAITAEVVATSALKASDGFTQLLPST-LVIVGYATAFYFLALT-
EmrE Methylococcus capsulatus YP_115175 MPWIHLMIAIIGEVVGTSALKSTDGFSRFWPSV-TVIAAYAVAFYFLSLT-
EmrE Coxiella burnetii ZP_01299513 MKWLYLLIAIVAEVIGTSALKASDGFTKFGAST-LVIIGYGIAFYFLSLT-
QacH Brucella melitensis YP_414370 PVYTFLAIAIFSEVIGTLSLKASEGFSRLGPSI-VVVVAYGLAFYFLSLT-
QacE Brucella melitensis AAL52226 PVYTFLAIAIFSEVIGTLSLKASEGFSRLGPSI-VVVVAYGLAFYFLSLT-
EmrE Ochrobactrum anthropi YP_001370804 PVYAILAIAIVSEVIGTLSLKASEGFTRLGPSL-IVVVAYGLAFYFLSMT-
EmrE Marinobacter algicola ZP_01894487 IQWVYLSVAIVAEVIGTSFLKSSEGFTRLGPSL-VVVVSYILAFYFLALT-
EmrE Nitrosococcus oceani YP_342650 MQWIFLSLAILAEVIATSSLKAAAGFTRLGPSL-AVILGYGAAFYFLSLT-
EmrE Reinekea sp. MED297 ZP_01116498 N-WLYLLIAIVAEVIATTSLKPAEGFTKLWPSV-VVVIGYAVAFFFLSLT-
EmrE Geobacter sulfurreducens NP_951765 HKWLYLLVAIISEVAGTTALKSAEGFTRLWPSC-VVVAGYASAFYFLSLT-
EmrE Chlorobium limicola EAM42528 H-WFYLITAIISEVVATTALKATDSFTRLWPSL-LVVAGYSSSFYFLSLT-
EmrE Alcanivorax borkumensis YP_693512 MGYLYLSLAIVAEVVATSALNASQGFTRLWPSV-TTVVGYTIAFYALSLAL
EmrE Jannaschia sp. CCS1 YP_511013 MAWLYLVFAILAEVIATSALKASEGFTQGPAAA-MTVVGYAVSFYFLGLAF
EmrE Chromohalobacter salexigens --YVFLAIAIIAEVVATSALKASQEFTRLWPSV-TVVVGYALAFYMLTLAL
QacH Pelagibacter ubique ZP_01264056 TSYLFLALAVVLGVASNSF-KSAEGFTLLVPSI-ITAITIVLCMYALSMVM
QacH2 Pelagibacter ubique YP_266208 GGYIFLVLAIILGISSNGF-KATDGFTNIYPTI-FCIITIVACIFCLSKAM
EmrE Pseudoalteromonas atlantica YP_661014 MSWVFLMLGVVAEAMSHVA-KETDGFTKPLPSV-LVLLGHVAAFMFLGQAM
QacF Pseudoalteromonas haloplanktis CAI87270 MSWVFLFLGVIAEAMSHVA-KQTDGFTRLLPSI-VVLLGHLAAFVFLGQAM
Qac megaplasmid (S. oneidensis) NP_720475 MSWIFLLLGVMAEALSHVA-KATDGFTRPIPAV-MVILGHLTAFIFLGQAM
YvaE Shewanella frigidimarina YP_752098 MSWIFLLLGVMAEALSHVA-KATDGFTRPLPAV-MVILGHLTAFIFLGQAM
EmrE Shewanella pealeana YP_001500134 MSWIFLLLGVVAEVLSHVA-KATDGFSKPLPAA-LVILGHLTAFIFLGQAM
EmrE Vibrio harveyi YP_001449011 MSWFFLLLGVGAEALSHVA-KATDGFSKPLPAT-LVLLGHLAAFVCLAQAM
EmrE Photobacterium profundum ZP_01219483 MGWVFLLLGVLAEATSHVA-KATNGFTNPLPSA-IVILGHLTAFLFLGQAM
EbrB Pseudomonas syringae YP_235623 KAYLFLLAGIVAETVATIA-KESNGFTRWVPVI-ASIAGYGLGIVCLGYAQ
EmrE Wigglesworthia glossinidia NP_871589 ICYIYLFIAILAEVFSTSL-KISEGFTKIFPSI-LIILGYIVSSFFLSLAL
EmrE Aeromonas hydrophila YP_855864 FGFLWLALSIGSEITGTS--KKTNGFSRLGPSI-LVVCAYSLCYFALTRAM
EmrE Erwinia carotovora YP_051860 NGFLWLALSIGSEITGTS--KKTDGFKKRTPSI-IVILAYCLCYFSLTRAM
EmrE Aeromonas hydrophila YP_855865 KCWLWMLLVIVSETSATSTLGSSEGTKMLLLG--LLVLLYCTCYYSLSRAV
QacE integron (Escherichia coli) YP_672432 KCWLWMLLVILSETSATSTLDNSEGMKTLLLA--LIVVLYCICYYSLSRAV
QacH Erwinia carotovora YP_051859 QSWLWMLLVILSETSATSTLDNSQGMKAALLA--IIILLYCVCYYSLSKAV
EmrE Nitrobacter winogradskyi YP_319204 NAWGLLAISIVAEVTATTLLTKSEGFGKPVYGV-AALAIFAGCFWALSQVL
EmrE Synechococcus sp. RS9917 ZP_01081426 SPWILLLFAIAAEVVGTSCLKLSEGFSRPLPTL-LVLAAYATSMALMSRVV
EbrB Mannheimia haemolytica EDN74090 HWSVLLGIAIVSEVFGSTMLKLSQGFTKPLPSI-GFVLGFGLAFYLLSLA-
EmrE Neisseria meningitidis NP_284788 HW-LFLTVAILSEVCGSSMLKLSGGFSKLWPSI-GVIVSFSVCFWALSMT-
EbrB Listeria monocytogenes EAL09800 KGYVALGIAIIGEIFGTSMLKLSEGFTNIYPTI-GVAIGFFIAFYTLSLS-
EbrA Paenibacillus larvae ZP_02326984 --YLFLTCSIISEVFGTTMLKFSDGFTVFLPTL-GIIAGFSIAFYCLSLC-
EbrB Bacillus subtillis CAB13613 RGLLYLALAIVSEVFGSTMLKLSEGFTQAWPIA-GVIVGFLSAFTFLSFS-
EbrB Paenibacillus larvae ZP_02326985 --YLTLAVSLTLEMFGATMLKLSKGFTKWLPAV-LVVFGYGSAFYFFSKS-
EbrA Bacillus subtillis CAB13614 IGYIFLTIAICSESIGAAMLKVSDGFKKWKPSA-LVVIGYSLAFYMLSLT-
QacG plasmid pST94 O87866 H-YLYLFISIATEIIGTSFLKTSEGFTKLWPTL-GTLLSFGICFYFLSLT-
QacJ plasmid pNVH01 (S. aureus) CAD55144 P-YLYLVIAIITEIIGTSFLKTAEGFTKLWPTL-GTLISFGICFYFLSVT-
QacH plasmid pST2H6 (S. saprophyticus) CAA76544 P-YLYLLLSIVSEVIGSAFLKSSDGFSKLYPTI-TTIISFLICFYFLSKT-
Smr Staphylococcus aureus AAM94142 P-YIYLIIAISTEVIGSAFLKSSEGFSKFIPSL-GTIISFGICFYFLSKT-
EbrA Desulfotomaculum reducens YP_001114614 A-YLYLILAILCELIGTSLLKASAGFSKLFPTL-GLLVSFSLAFFFLSLS-
Ebr Pediococcus pentosaceus YP_804449 NGYVYLLVAIVAELLGTNFLKLTNGFTKLLPTL-ISLLSYGFAFYFLSLV-
Ebr1 Synechococcus sp. RS9917 ZP_01080450 LGYAVLIAAILFENIGTTALKGSNGFKRPVLGS-VAVLGYILSFAFMGQA-
EbrA Listeria monocytogenes EAL09801 NPYVLLIGAILFEVFGSSMMKASNGFKKLVPTV-GLVIGMGSAFYLLSKA-
Smr Roseiflexus castenholzii ZP_01530339 RGMMLLFGAIVCELIGTNALKAADGFTRLMPTL-LVIAGYGASFYLMSLS-
EbrA Roseiflexus sp. RS-1 YP_001278866 RGLALLFGAIVCELIGTSALKATDGFTRLLPTL-LVIAGYGASFYLMSLS-
EbrA Gloeobacter violaceus NP_927270 HPLILLAVAIASEVIGSSALKLSEGFSRPLPSL-LVVLGYGAAFYLLGLT-
EbrB Thermus thermophilus YP_004540 SGWTYLFLAILSEVLASSALKASQGFSRLLPSL-VVVGGYGLAFYFLSLA-
EbrB Halobacterium sp. NRC1 NP_444228 HPYAYLAAAIAAEVAGTTALKLSEGFSNPAPSV-VVLVGYVSSFYFLGLVL
EmrE Zymomonas mobilis sbsp. mobilis YP_161843 TGWTGLAIAVFFEVAATTALRLANGIDKPLWLI-TACIGYFICFTIISFVF
Smr2 Mycobacterium vanbaalenii YP_954149 MTWLYLMLAICTEVAATLSLKGLA--TVPALYI-VVVLGYVASFVFLALVL
Smr2 Mycobacterium gilvum YP_001134816 -MWVFLILAISSEVAATLSLKGSE--TVPALYA-VVVLGYLASFVFLALVL

Sequence	10 20 30 40 50
EbrB *Clavibacter michiganensis* YP_001220961	MAWLLLGIAIVTEVAATISLKLATD-GRKRWYA-VVAVGYVTAFSMLAGAL
Smr *Nocardia farcinica* YP_118186	MTLLLLALAIASEVTATVSLKLSEGFTKLTPSI-VVVVGYCAAFYFLSQAL
Smr *Saccharopolyspora erythraea* YP_001109297	--YLLLAVAILSEVTATVSLKLSEGFSKPVPSV-MVVVGYLAAFAALGAVL
Smr *Thermobifida fusca* YP_289048	MQWLLLIGAILTEVTGTISLRLSDGFTRLVPSL-IALTSYGIAFTLLAQVL
Smr *Saccharopolyspora erythraea* YP_001103712	MAYLLLLGAVASEVVAALATRFSAGFTKLVPSA-VAITGVVGAYYLLSLAL
Smr *Nocardioides sp.* JS614 YP_921842	VTYLLLGLAIAVEVMATLSLRASEGFSRPLPSL-IVVSGYGLSFFLLSVVL
Smr *Nocardioides sp.* JS614 YP_921842	VAWTLLGGAILAEVIATSMLKSTEGFTRLWPTL-ACLLLYGAAFMLLAQSI
Smr *Mycobacterium tuberculosis* P95094	MIYLYLLCAIFAEVVATSLLKSTEGFTRLWPTV-GCLVGYGIAFALLALSI
NepA plasmid pAO1 (*A. nicotinovorans*) CAD47919	TIWPLLLLAIAAEVAATSLLPQTNGFRKLKPTV-AVACLYTVAFALLAQIL
EmrE *Candidatus Pelagibacter ubique* ZP_01264207	KTYLFLTIAIFCEVGGTMLLPVSQNFTKIIPTT-TLAILYLSSFYLLTFVV
QacH *Lactobacillus plantarum* NP_786542	MAYGLLFGGIFMEVLGSFFLKRANGFRNLIPTI-MVLVGYFSSLVLVTLAM
YdgF *Escherichia coli* ABE07266	IYWILLGLAIATEITGTLSMWASVSE---NGGFILMLVMISLSYIFLSFAV
YdgF *Shigella flexneri* YP_689093	IYWILLGLAIATEITGTLSMWASVSE---NGGFILMLVMISLSYIFLSFAV
YdgF *Salmonella enterica sbsp. enterica* YP_216487	FYWILLALAIATEITGTLSMWASVGN---NAGFILMLVMITLSYIFLSFAV
YdgF *Yersinia intermedia* ZP_00833156	IYWIFLGLAIVAEIIGTLSMYASVSG---MAGHIVMYFMITGSYILLALAV
YdgF *Photorhabdus luminescens* NP_929383	IYWLFLAMAIITEVIGTLSMHASVSG---VVGMAVMYIMIATSYILLAMAV
YdgF *Pseudomonas aeruginosa* NP_250232	RSWIYLLLAIGAEVIGTTSMLAATHA---VAGMLLMYGMIGLSYFFLALAV
YdgF *Sinorhizobium meliloti* NP_385720	LAWLFLVLAIATEVVGLTVMAASGSG---YWGHVVMYASIALSYVFLARAV
YdgF *Aeromonas hydrophila* YP_857997	LARIFLTGAILTEVAGTSSMMIPENEG--WLNYLPMWLLISFSYLLLAKAA
YdgF *Vibrio harveyi*	---LFLLLAIVAEVAGTSTMFIGESN---WEGYLVMYVLIAISYYFLSKAA
YdgF *Chromobacterium violaceum* NP_900949	VAWLVLLAAIVCEIVGTSFMVAARSG---YLGYVWMSLALALSYYLLSLAL
YdgF *Campylobacter jejuni* YP_179294	VAWFFLIAAIVFEVLGTSFLKM-ENQ---VLGYIFMALFIAFSYFFMGKAI
YdgF *Desulfovibrio desulfuricans* YP_388898	-HWLCLTGAVFFEVAGTTLMSQSWSMFGAEAGVVIMLLFIGVSYYLLSLST
YdgF *Lawsonia intracellularis* CAJ54979	KHWIYLIIAILLEVIGTSVMSQLGTWFEPNIGYLLMLICIGLSYYMLALAA
YdgF *Deinococcus geothermalis* YP_605419	-AWIALFSAIACEVTGTLALLGLHTP---WLALAVTGSLIVLSYLLLSLVF
YdgF *Pseudoalteromonas haloplanktis* YP_340952	MHWIYLIIAVTLEVIGTTVMWLVNED-QLLLGSLFVTLMVGLAYLALSQAT
YdgF *Acidovorax avenae subsp. citrulli* YP_969092	QPWLFLLAAVASEVVGVTTMLVAQEH--DWGALLFMYSTIGLSFAFLAMAM
YdgF *Helicobacter hepaticus* NP_860040	-SYLFLMFAILTEVLAVNLMKASEGE---IWGLMCMYVLLVLSYYFMALSL
EmrE *Alteromonas macleodii* ZP_01110205	MTYLLLAIAIVTEVTATLLLKMSNGWKWAFGYG--AIFFYTVSGMLFAMVL
YvaE *Bacillus subtilis* CAB15362	MNWVFLCLAILFEVAGTVSMKLSSGFTKLIPSL-LLIFFYGGSLFFLTLTL
YvaE *Blastopirellula marina* ZP_01094237	QPWVFLAGAILLEVAGTTSMKMSEGFTRPLPSL-LLFFFYAGSFTALTFAL
YvaE *Desulfitobacterium hafniense* YP_517233	MQWVYLALAILLEILGTTLMKMSEGLTKMLPTL-GMFLAYVLCFSSFALAL
YvaE *Rhodospirillum rubrum* YP_428434	MTWLTLTGAILFELAGTTAMKLSEGFTRLLPSV-ALVVCYAISFTLLTLTL
YvaE *Bdellovibrio bacteriovorus* NP_968626	MAYVYLAAAIIFEVFGTITMKYSEGFTKVLPSV-LTVACHGICFISLTVAL
Smr *Streptomyces coelicolor* AAK95484	MGYLLLAGAIAAEVAGTTAMKYSEGFSRLVPSL-LTALAYVLSFALLAQTL
YvaE *Acidobacteria bacterium* YP_593828	MCWLWLFLAIALEVAATILMKYSDGFTRFGPTV-GMLVLYALSLAPLARVL
EbrB *Rubrobacter xylanophilus* YP_644095	MSWLLLLGAIAAEVAGTFSLKLSDGLSRAGPTL-MVVVFYGLSFYLLSLVL
YvaE *Desulfovibrio vulgaris* YP_966624	ESWIILFLSIFCEVTGTSCLKLSDGFSKIIPTI-SVFLFYGLALWGLSVVV
YvaE *Pelodictyon phaeoclathratiforme* ZP_00590439	MHWIYLIMAILFEVSGTTCMKISDGFSKLAPTL-FIFIFYGLSFTFLSLAL
YvaE *Chlorobium tepidum* NP_662342	MPWLYLILAIIAEVSGTTSMKLSAGFTKPVPSV-FIFVFYATSLTFLTLAL
YvaE *Prosthecochloris vibrioformis* YP_001130806	MHWIALFMAIATEVIGTTFLKLSDGFSKPLPAV-LVFIFYGLSFTLLSQAL
YvaE *Anabaena variabilis* YP_323581	TSLVYLLMAIIFEVSGTTCMKLSDGFNKLTPSI-LIFVFYGLCFTFQTLSL
YvaE *Nostoc sp.* PCC 7120 NP_484329	TSLVYLLIAIIFEVSGTTCMKLSDGFNKLTPSI-LIFVFYGLCFTFQTLSL
EbrA *Methanosarcina acetivorans* NP_616284	NYYYLLLLAIAFEVCGTTCMKLSEGFTKLLPSV-LIFVFYAISFFLFTLAL
YvaE *Methanococcoides burtonii* YP_566929	MSYSLLAISIFFEICGTVCMKISDGYSNISASL-MIFVFYGISFSIFPFAL
YvaE *Clostridium beijerinckii* YP_001307448	ISWIYLIFAIIFEVSGTISMKLSEGFTNIKYAI-VMLGFYILSLSMLTLAL
YvaE *Marinomonas sp.* MED121 ZP_01075592	SGWTLLIIGIAFEVAGSLCLKLSHGFQNFVPTI-FCFFFFALALSMINLSV
QacC *Lactobacillus plantarum* NP_786541	RAWIQLVIAIGVEIVGTSLLKLSSGFKYPLIGI-VGMLLYGLAIFSFSRAL
NepB plasmid pAO1 (*A. nicotinovorans*) CAD47918	HAWLYLGSAITTEVTGTVILDFSEGFQLPAQTT-AAMALYAFSFFLLTRAL
YvaE *Burkholderia cenocepacia* YP_839289	FAWFVLGLSVIAEVFGTIGLKFSSGFTHLPSSV-FTVVCYAGAVWLMSITT
EmrE *Synechococcus sp.* RS9917 ZP_01080451	GALFELFLAIAAEQIGTSAMKASNGFTQLPLTV-LALAGYALSMLWFGRSM
SugE *Escherichia coli* AAC46453	--WIILVIAGLLEVVWAVGLKYTHGFSRLTPSV-ITVTAMIVSMALLAWAM
SugE *Shigella flexneri* YP_691592	--WIILVIAGLLEVVWAVGLKYTHGFSRLTPSV-ITVTAMIVSMALLAWAM
SugE *Salmonella enterica sbsp. enterica* YP_219204	--WIILLIAGLLEVVWAVGLKYTHGFSRLTPSI-ITITAMVISMALLSWAM
SugE plasmid/ *Citrobacter freundii* AAC46457	--WIVLLIAGLLEVVWAIGLKYTHGFTRLTPSI-ITIAAMIVSIAMLSWAM
SugE *Serratia proteamaculans* ZP_01538212	--WIILLIAGLLEVVWAIGLKYTHGFTRLTPSL-ITIAAMVVSMLLLAHAM
SugE *Yersinia intermedia* ZP_00832613	--WIILVIAGLLEVIWAIGLKYSHGFTRLTPSV-ITLVAMAASVFLLAYAM
SugE *Photorhabdus luminescens* NP_931319	--WFVLVIAGLFEVVWAVGLKYTHGFTRLIPSL-ITASAVALSMGLLAYAM
SugE *Proteus vulgaris* P20928	--WIILFVAGLLEIVWAVGLKYTHGFTRLTPSI-ITISAMIVSMGMLSYAM

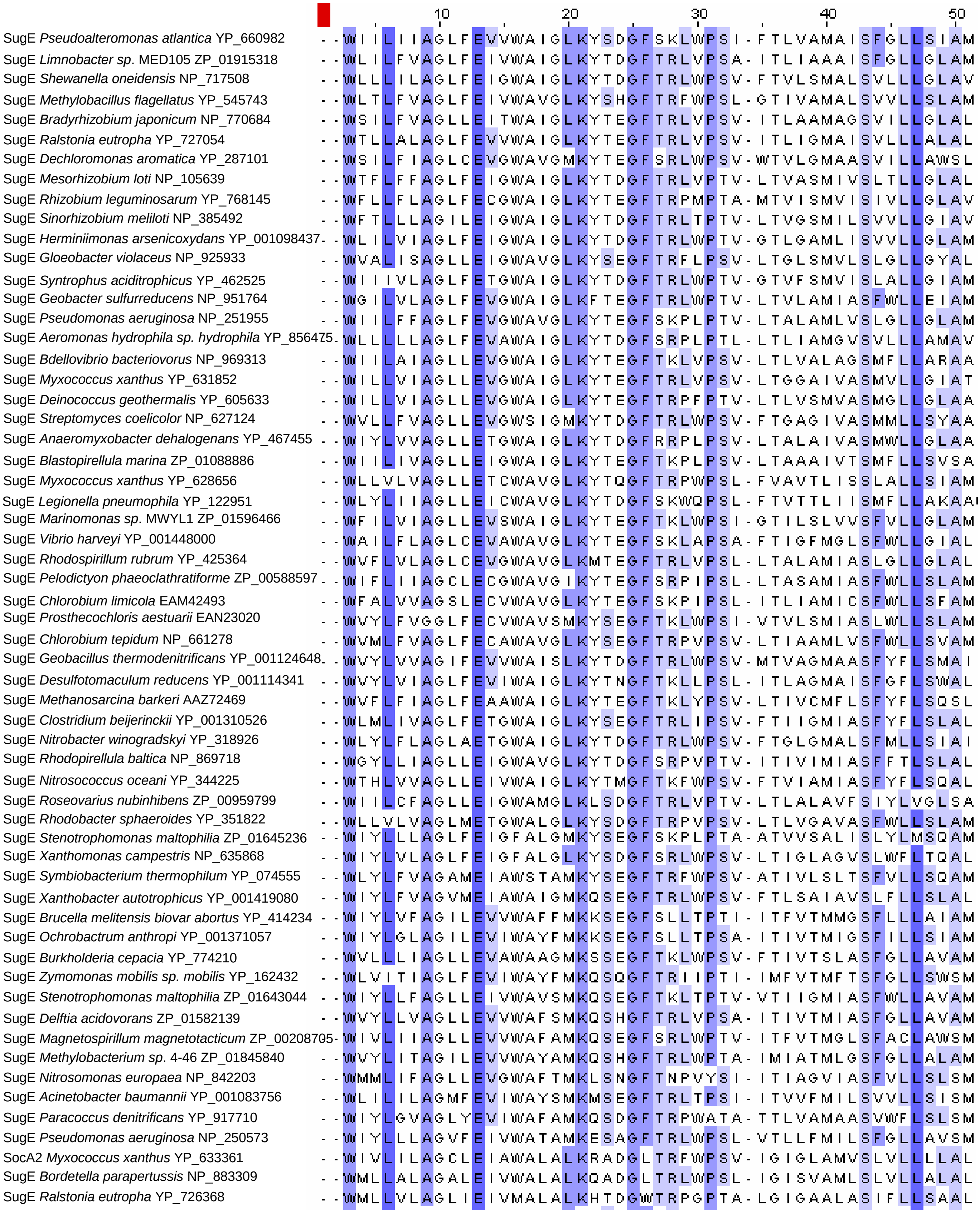

10 20 30 40 50
SugE Pseudoalteromonas atlantica YP_660982 --WIILIIAGLFEVVWAIGLKYSDGFSKLWPSI-FTLVAMAISFGLLSIAM
SugE Limnobacter sp. MED105 ZP_01915318 --WLILFVAGLFEIVWAIGLKYTDGFTRLVPSA-ITLIAAAISFGLLGLAM
SugE Shewanella oneidensis NP_717508 --WLLLILAGLFEIGWAIGLKYTDGFTRLWPSV-FTVLSMALSVLLLGLAV
SugE Methylobacillus flagellatus YP_545743 --WLTLFVAGLFEIVWAVGLKYSHGFTRFWPSL-GTIVAMALSVVLLSLAM
SugE Bradyrhizobium japonicum NP_770684 --WSILFVAGLLEITWAIGLKYTEGFTRLVPSV-ITLAAMAGSVILLGLAL
SugE Ralstonia eutropha YP_727054 --WTLLALAGLFEVVWAIGLKYTEGFTRLVPSV-ITLIGMAISVLLLALAL
SugE Dechloromonas aromatica YP_287101 --WSILFIAGLCEVGWAVGMKYTEGFSRLWPSV-WTVLGMAASVILLAWSL
SugE Mesorhizobium loti NP_105639 --WTFLFFAGLFEIGWAIGLKYTDGFTRLVPTV-LTVASMIVSLTLLGLAL
SugE Rhizobium leguminosarum YP_768145 --WFLLFLAGLFECGWAIGLKYTEGFTRPMPTA-MTVISMVISIVLLGLAV
SugE Sinorhizobium meliloti NP_385492 --WFTLLLAGILEIGWAIGLKYTDGFTRLTPTV-LTVGSMILSVVLLGIAV
SugE Herminiimonas arsenicoxydans YP_001098437 -WLILVIAGLFEIGWAIGLKYTDGFTRLWPTV-GTLGAMLISVVLLGLAM
SugE Gloeobacter violaceus NP_925933 --WVALISAGLLEIGWAVGLKYSEGFTRFLPSV-LTGLSMVLSLGLLGYAL
SugE Syntrophus aciditrophicus YP_462525 --WIIIVLAGLFETGWAIGLKYTDGFTRLWPTV-GTVFSMVISLALLGIAM
SugE Geobacter sulfurreducens NP_951764 --WGILVLAGLFEVGWAIGLKFTEGFTRLWPTV-LTVLAMIASFWLLEIAM
SugE Pseudomonas aeruginosa NP_251955 --WIILFFAGLFEVGWAVGLKYTEGFSKPLPTV-LTALAMLVSLGLLGLAM
SugE Aeromonas hydrophila sp. hydrophila YP_856475 -WLLLLLAGLFEVAWAIGLKYTDGFSRPLPTL-LTLIAMGVSVLLLAMAV
SugE Bdellovibrio bacteriovorus NP_969313 --WIILAIAGLLEVGWAIGLKYTEGFTKLVPSV-LTLVALAGSMFLLARAA
SugE Myxococcus xanthus YP_631852 --WILLVIAGLLEVGWAIGLKYTEGFTRLVPSV-LTGGAIVASMVLLGIAT
SugE Deinococcus geothermalis YP_605633 --WILLVIAGLLEVGWAIGLKYTEGFTRPFPTV-LTLVSMVASMGLLGLAA
SugE Streptomyces coelicolor NP_627124 --WVLLFVAGLLEVGWSIGMKYTDGFTRLWPSV-FTGAGIVASMMLLSYAA
SugE Anaeromyxobacter dehalogenans YP_467455 --WIYLVVAGLLETGWAIGLKYTDGFRRPLPSV-LTALAIVASMWLLGLAA
SugE Blastopirellula marina ZP_01088886 --WIILIVAGLLEIGWAIGLKYTEGFTKPLPSV-LTAAAIVTSMFLLSVSA
SugE Myxococcus xanthus YP_628656 --WLLVLVAGLLETCWAVGLKYTQGFTRPWPSL-FVAVTLISSLALLSIAM
SugE Legionella pneumophila YP_122951 --WLYLIIAGLLEICWAVGLKYTDGFSKWQPSL-FTVTTLIISMFLLAKAA
SugE Marinomonas sp. MWYL1 ZP_01596466 --WFILVIAGLLEVSWAIGLKYTEGFTKLWPSI-GTILSLVVSFVLLGLAM
SugE Vibrio harveyi YP_001448000 --WAILFLAGLCEVAWAVGLKYTEGFSKLAPSA-FTIGFMGLSFWLLGIAL
SugE Rhodospirillum rubrum YP_425364 --WVFLVLAGLCEVGWAVGLKMTEGFTRLVPSL-LTALAMIASLGLLGLAL
SugE Pelodictyon phaeoclathratiforme ZP_00588597 --WIFLIIAGCLECGWAVGIKYTEGFSRPIPSL-LTASAMIASFWLLSLAM
SugE Chlorobium limicola EAM42493 --WFALVVAGSLECVWAVGLKYTEGFSKPIPSL-ITLIAMICSFWLLSFAM
SugE Prosthecochloris aestuarii EAN23020 --WVYLFVGGLFECVWAVSMKYSEGFTKLWPSI-VTVLSMIASLWLLSLAM
SugE Chlorobium tepidum NP_661278 --WVMLFVAGLFECAWAVGLKYSEGFTRPVPSV-LTIAAMLVSFWLLSVAM
SugE Geobacillus thermodenitrificans YP_001124648 -WVYLVVAGIFEVVWAISLKYTDGFTRLWPSV-MTVAGMAASFYFLSMAI
SugE Desulfotomaculum reducens YP_001114341 --WVYLVIAGLFEVIWAIGLKYTNGFTKLLPSL-ITLAGMAISFGFLSWAL
SugE Methanosarcina barkeri AAZ72469 --WVFLFIAGLFEAAWAIGLKYTEGFTKLYPSV-LTIVCMFLSFYFLSQSL
SugE Clostridium beijerinckii YP_001310526 --WLMLIVAGLFETGWAIGLKYSEGFTRLIPSV-FTIIGMIASFYFLSLAL
SugE Nitrobacter winogradskyi YP_318926 --WLYLFLAGLAETGWAIGLKYTDGFTRLWPSV-FTGLGMALSFMLLSIAI
SugE Rhodopirellula baltica NP_869718 --WGYLLIAGLLEIGWAVGLKYTDGFSRPVPTV-ITIVIMIASFFTLSLAL
SugE Nitrosococcus oceani YP_344225 --WTHLVVAGLLEIVWAIGLKYTMGFTKFWPSV-FTVIAMIASFYFLSQAL
SugE Roseovarius nubinhibens ZP_00959799 --WIILCFAGLLEIGWAMGLKLSDGFTRLVPTV-LTLALAVFSIYLVGLSA
SugE Rhodobacter sphaeroides YP_351822 --WLLVLVAGLMETGWALGLKYSDGFTRPVPSV-LTLVGAVASFWLLSLAM
SugE Stenotrophomonas maltophilia ZP_01645236 --WIYLLLAGLFEIGFALGMKYSEGFSKPLPTA-ATVVSALISLYLMSQAM
SugE Xanthomonas campestris NP_635868 --WIYLVLAGLFEIGFALGLKYSDGFSRLWPSV-LTIGLAGVSLWFLTQAL
SugE Symbiobacterium thermophilum YP_074555 --WLYLFVAGAMEIAWSTAMKYSEGFTRFWPSV-ATIVLSLTSFVLLSQAM
SugE Xanthobacter autotrophicus YP_001419080 --WIYLFVAGVMEIAWAIGMKQSEGFTRLWPSL-FTLSAIAVSLFLLSLAL
SugE Brucella melitensis biovar abortus YP_414234 --WIYLVFAGILEVVWAFFMKKSEGFSLLTPTI-ITFVTMMGSFLLLAIAM
SugE Ochrobactrum anthropi YP_001371057 --WIYLGLAGILEVIWAYFMKKSEGFSLLTPSA-ITIVTMIGSFILLSIAM
SugE Burkholderia cepacia YP_774210 --WVLLLIAGLLEVAWAAGMKSSEGFTKLWPSV-FTIVTSLASFGLLAVAM
SugE Zymomonas mobilis sp. mobilis YP_162432 --WLVITIAGLFEVIWAYFMKQSQGFTRIIPTI-IMFVTMFTSFGLLSWSM
SugE Stenotrophomonas maltophilia ZP_01643044 --WIYLLFAGLLEIVWAVSMKQSEGFTKLTPTV-VTIIGMIASFWLLAVAM
SugE Delftia acidovorans ZP_01582139 --WVYLLVAGLLEVVWAFSMKQSHGFTRLVPSA-ITIVTMIASFGLLAVAM
SugE Magnetospirillum magnetotacticum ZP_00208705 -WIVLIIAGLLEVVWAFAMKQSEGFSRLWPTV-ITFVTMGLSFACLAWSM
SugE Methylobacterium sp. 4-46 ZP_01845840 --WVYLITAGILEVVWAYAMKQSHGFTRLWPTA-IMIATMLGSFGLLALAM
SugE Nitrosomonas europaea NP_842203 --WMMLIFAGLLEVGWAFTMKLSNGFTNPVYSI-ITIAGVIASFVLLSLSM
SugE Acinetobacter baumannii YP_001083756 --WLILILAGMFEVIWAYSMKMSEGFTRLTPSI-ITVVFMILSVVLLSISM
SugE Paracoccus denitrificans YP_917710 --WIYLGVAGLYEVIWAFAMKQSDGFTRPWATA-TTLVAMAASVWFLSLSM
SugE Pseudomonas aeruginosa NP_250573 --WIYLLLAGVFEIVWATAMKESAGFTRLWPSL-VTLLFMILSFGLLAVSM
SocA2 Myxococcus xanthus YP_633361 --WIVLILAGCLEIAWALALKRADGLTRFWPSV-IGIGLAMVSLVLLLLAL
SugE Bordetella parapertussis NP_883309 --WMLLALAGALEIVWALALKQADGLTRLWPSL-IGISVAMLSLVLLALAL
SugE Ralstonia eutropha YP_726368 --WMLLVLAGLIEIVMALALKHTDGWTRPGPTA-LGIGAALASIFLLSAAL

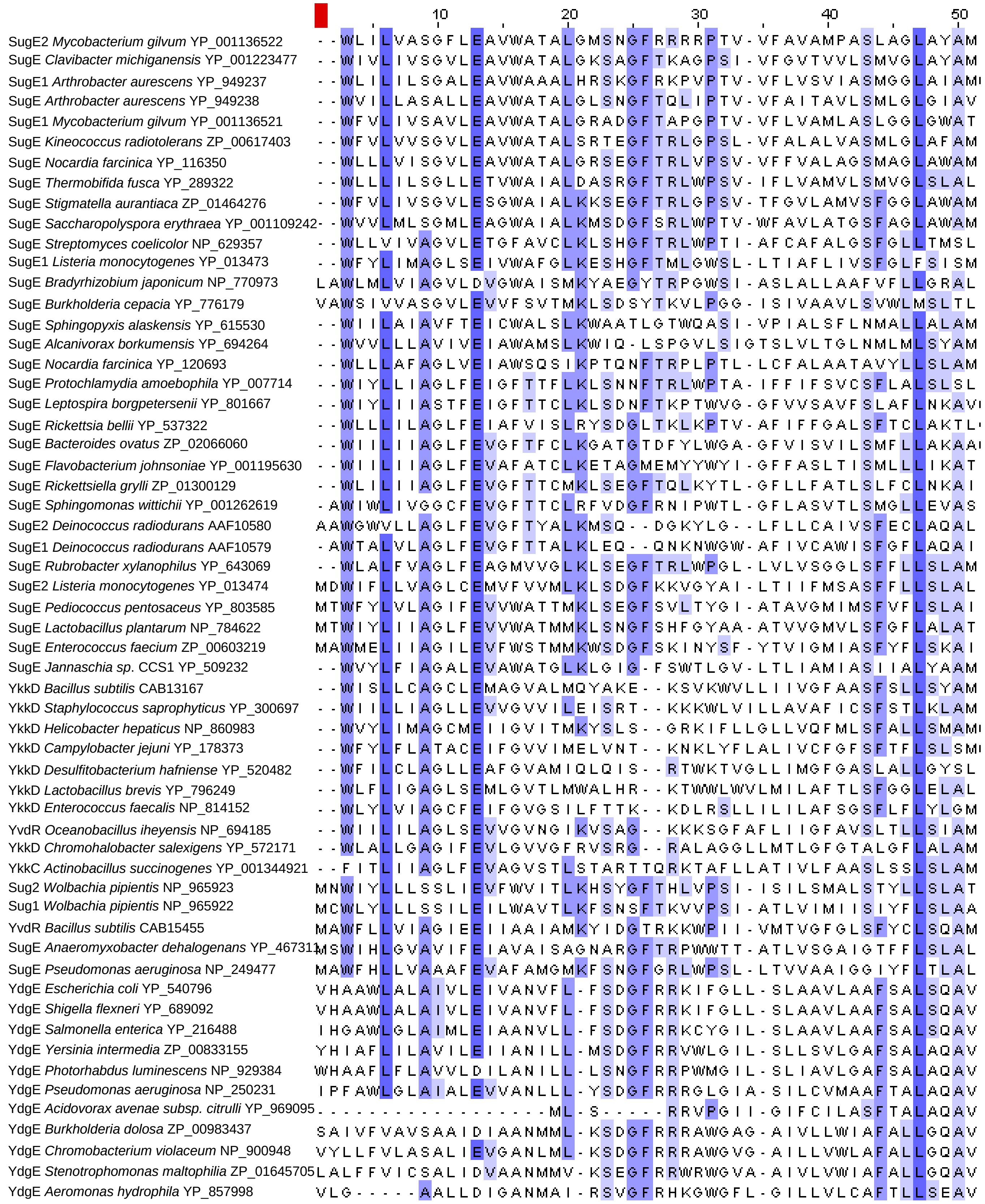
10 20 30 40 50
SugE2 Mycobacterium gilvum YP_001136522 --WLILVASGFLEAVWATALGMSNGFRRRRPTV-VFAVAMPASLAGLAYAM
SugE Clavibacter michiganensis YP_001223477 --WIVLIVSGVLEAVWATALGKSAGFTKAGPSI-VFGVTVVLSMVGLAYAM
SugE1 Arthrobacter aurescens YP_949237 --WLILILSGALEAVWAAALHRSKGFRKPVPTV-VFLVSVIASMGGLAIAM
SugE Arthrobacter aurescens YP_949238 --WVILLASALLEAVWATALGLSNGFTQLIPTV-VFAITAVLSMLGLGIAV
SugE1 Mycobacterium gilvum YP_001136521 --WFVLIVSAVLEAVWATALGRADGFTAPGPTV-VFLVAMLASLGGLGWAT
SugE Kineococcus radiotolerans ZP_00617403 --WFVLVVSGVLEAVWATALSRTEGFTRLGPSL-VFALALVASMLGLAFAM
SugE Nocardia farcinica YP_116350 --WLLLVISGVLEAVWATALGRSEGFTRLVPSV-VFFVALAGSMAGLAWAM
SugE Thermobifida fusca YP_289322 --WLLLILSGLLETVWAIALDASRGFTRLWPSV-IFLVAMVLSMVGLSLAL
SugE Stigmatella aurantiaca ZP_01464276 --WFVLIVSGVLESGWAIALKKSEGFTRLGPSV-TFGVLAMVSFGGLAWAM
SugE Saccharopolyspora erythraea YP_001109242 --WVVLMLSGMLEAGWAIALKMSDGFSRLWPTV-WFAVLATGSFAGLAWAM
SugE Streptomyces coelicolor NP_629357 --WLLVIVAGVLETGFAVCLKLSHGFTRLWPTI-AFCAFALGSFGLLTMSL
SugE1 Listeria monocytogenes YP_013473 --WFYLIMAGLSEIVWAFGLKESHGFTMLGWSL-LTIAFLIVSFGLFSISM
SugE Bradyrhizobium japonicum NP_770973 LAWLMLVIAGVLDVGWAISMKYAEGYTRPGWSI-ASLALLAAFVFLLGRAL
SugE Burkholderia cepacia YP_776179 VAWSIVVASGVLEVVFSVTMKLSDSYTKVLPGG-ISIVAAVLSVWLMSLTL
SugE Sphingopyxis alaskensis YP_615530 --WIILAIAVFTEICWALSLKWAATLGTWQASI-VPIALSFLNMALLALAM
SugE Alcanivorax borkumensis YP_694264 --WVVLLLAVIVEIAWAMSLKWIQ-LSPGVLSIGTSLVLTGLNMLMLSYAM
SugE Nocardia farcinica YP_120693 --WLLLAFAGLVEIAWSQSIKPTQNFTRPLPTL-LCFALAATAVYLLSLAM
SugE Protochlamydia amoebophila YP_007714 --WIYLLIAGLFEIGFTTFLKLSNNFTRLWPTA-IFFIFSVCSFLALSLSL
SugE Leptospira borgpetersenii YP_801667 --WIYLIIASTFEIGFTTCLKLSDNFTKPTWVG-GFVVSAVFSLAFLNKAV
SugE Rickettsia bellii YP_537322 --WLLLILAGLFEIAFVISLRYSDGLTKLKPTV-AFIFFGALSFTCLAKTL
SugE Bacteroides ovatus ZP_02066060 --WIILIIAGLFEVGFTFCLKGATGTDFYLWGA-GFVISVILSMFLLAKAA
SugE Flavobacterium johnsoniae YP_001195630 --WIILIIAGLFEVAFATCLKETAGMEMYYWYI-GFFASLTISMLLLIKAT
SugE Rickettsiella grylli ZP_01300129 --WLILIIAGLFEVGFTTCMKLSEGFTQLKYTL-GFLLFATLSLFCLNKAI
SugE Sphingomonas wittichii YP_001262619 -AWIWLIVGGCFEVGFTTCLRFVDGFRNIPWTL-GFLASVTLSMGLLEVAS
SugE2 Deinococcus radiodurans AAF10580 AAWGWVLLAGLFEVGFTYALKMSQ--DGKYLG--LFLLCAIVSFECLAQAL
SugE1 Deinococcus radiodurans AAF10579 -AWTALVLAGLFEVGFTTALKLEQ--QNKNWGW-AFIVCAWISFGFLAQAI
SugE Rubrobacter xylanophilus YP_643069 --WLALFVAGLFEAGMVVGLKLSEGFTRLWPGL-LVLVSGGLSFFLLSLAM
SugE2 Listeria monocytogenes YP_013474 MDWIFLLVAGLCEMVFVVMLKLSDGFKKVGYAI-LTIIFMSASFFLLSLAL
SugE Pediococcus pentosaceus YP_803585 MTWFYLVLAGIFEVVWATTMKLSEGFSVLTYGI-ATAVGMIMSFVFLSLAI
SugE Lactobacillus plantarum NP_784622 MTWIYLIIAGLFEVVWATMMKLSNGFSHFGYAA-ATVVGMVLSFGFLALAT
SugE Enterococcus faecium ZP_00603219 MAWMELIIAGILEVFWSTMMKWSDGFSKINYSF-YTVIGMIASFYFLSKAI
SugE Jannaschia sp. CCS1 YP_509232 --WVYLFIAGALEVAWATGLKLGIG-FSWTLGV-LTLIAMIASIIALYAAM
YkkD Bacillus subtilis CAB13167 --WISLLCAGCLEMAGVALMQYAKE--KSVKWVLLIIVGFAASFSLLSYAM
YkkD Staphylococcus saprophyticus YP_300697 --WIILLIAGLLEVVGVVILEISRT--KKKWLVILLAVAFICSFSTLKLAM
YkkD Helicobacter hepaticus NP_860983 --WVYLIMAGCMEIIGVITMKYSLS--GRKIFLLGLLVQFMLSFALLSMAM
YkkD Campylobacter jejuni YP_178373 --WFYLFLATACEIFGVVIMELVNT--KNKLYFLALIVCFGFSFTFLSLSM
YkkD Desulfitobacterium hafniense YP_520482 --WFILCLAGLLEAFGVAMIQLQIS--RTWKTVGLLIMGFGASLALLGYSL
YkkD Lactobacillus brevis YP_796249 --WLFLIGAGLSEMLGVTLMWALHR--KTWWLWVLMILAFTLSFGGLELAL
YkkD Enterococcus faecalis NP_814152 --WLYLVIAGCFEIFGVGSILFTTK--KDLRSLLILILAFSGSFLFLYLGM
YvdR Oceanobacillus iheyensis NP_694185 --WIILILAGLSEVVGVNGIKVSAG--KKKSGFAFLIIGFAVSLTLLSIAM
YkkD Chromohalobacter salexigens YP_572171 --WLALLGAGIFEVLGVVGFRVSRG--RALAGGLLMTLGFGTALGFLALAM
YkkC Actinobacillus succinogenes YP_001344921 --FITLIIAGLFEVAGVSTLSTARTTQRKTAFLLATIVLFAASLSSLSLAM
Sug2 Wolbachia pipientis NP_965923 MNWIYLLLSSLIEVFWVITLKHSYGFTHLVPSI-ISILSMALSTYLLSLAT
Sug1 Wolbachia pipientis NP_965922 MCWLYLLLSSILEILWAVTLKFSNSFTKVVPSI-ATLVIMIISIYFLSLAA
YvdR Bacillus subtilis CAB15455 MAWFLLVIAGIEEIIAAIAMKYIDGTRKKWPII-VMTVGFGLSFYCLSQAM
SugE Anaeromyxobacter dehalogenans YP_467311 MSWIHLGVAVIFEIAVAISAGNARGFTRPWWTT-ATLVSGAIGTFFLSLAL
SugE Pseudomonas aeruginosa NP_249477 MAWFHLLVAAAFEVAFAMGMKFSNGFGRLWPSL-LTVVAAIGGIYFLTLAL
YdgE Escherichia coli YP_540796 VHAAWLALAIVLEIVANVFL-FSDGFRRKIFGLL-SLAAVLAAFSALSQAV
YdgE Shigella flexneri YP_689092 VHAAWLALAIVLEIVANVFL-FSDGFRRKIFGLL-SLAAVLAAFSALSQAV
YdgE Salmonella enterica YP_216488 IHGAWLGLAIMLEIAANVLL-FSDGFRRKCYGIL-SLAAVLAAFSALSQAV
YdgE Yersinia intermedia ZP_00833155 YHIAFLILAVILEIIANILL-MSDGFRRVWLGIL-SLLSVLGAFSALAQAV
YdgE Photorhabdus luminescens NP_929384 WHAAFLFLAVVLDILANILL-LSNGFRRPWMGIL-SLIAVLGAFSALAQAV
YdgE Pseudomonas aeruginosa NP_250231 IPFAWLGLAIALEVVANLLL-YSDGFRRRGLGIA-SILCVMAAFTALAQAV
YdgE Acidovorax avenae subsp. citrulli YP_969095 ------------------ML-S-----RRVPGII-GIFCILASFTALAQAV
YdgE Burkholderia dolosa ZP_00983437 SAIVFVAVSAAIDIAANMML-KSDGFRRRAWGAG-AIVLLWIAFALLGQAV
YdgE Chromobacterium violaceum NP_900948 VYLLFVLASALIEVGANLML-KSDGFRRRAWGVG-AILLVWLAFALLGQAV
YdgE Stenotrophomonas maltophilia ZP_01645705 LALFFVICSALIDVAANMMV-KSEGFRRWRWGVA-AIVLVWIAFALLGQAV
YdgE Aeromonas hydrophila YP_857998 VLG-----AALLDIGANMAI-RSVGFRHKGWGFL-GILLVLCAFTLLSEAV

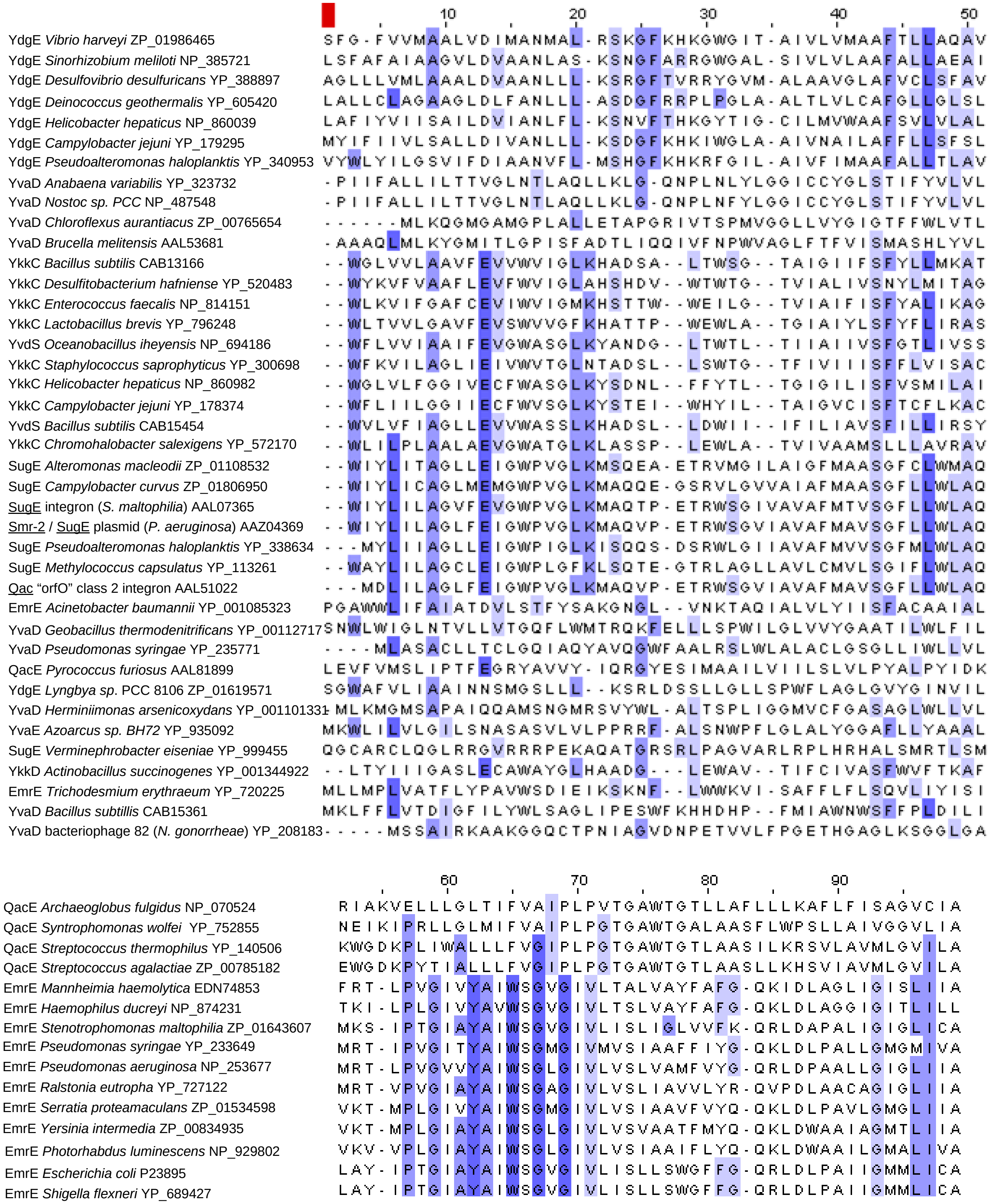
10
20
30
40
50
YdgE Vibrio harveyi ZP_01986465
YdgE Sinorhizobium meliloti NP_385721
YdgE Desulfovibrio desulfuricans YP_388897
YdgE Deinococcus geothermalis YP_605420
YdgE Helicobacter hepaticus NP_860039
YdgE Campylobacter jejuni YP_179295
YdgE Pseudoalteromonas haloplanktis YP_340953
YvaD Anabaena variabilis YP_323732
YvaD Nostoc sp. PCC NP_487548
YvaD Chloroflexus aurantiacus ZP_00765654
YvaD Brucella melitensis AAL53681
YkkC Bacillus subtilis CAB13166
YkkC Desulfitobacterium hafniense YP_520483
YkkC Enterococcus faecalis NP_814151
YkkC Lactobacillus brevis YP_796248
YvdS Oceanobacillus iheyensis NP_694186
YkkC Staphylococcus saprophyticus YP_300698
YkkC Helicobacter hepaticus NP_860982
YkkC Campylobacter jejuni YP_178374
YvdS Bacillus subtilis CAB15454
YkkC Chromohalobacter salexigens YP_572170
SugE Alteromonas macleodii ZP_01108532
SugE Campylobacter curvus ZP_01806950
SugE integron (S. maltophilia) AAL07365
Smr-2 / SugE plasmid (P. aeruginosa) AAZ04369
SugE Pseudoalteromonas haloplanktis YP_338634
SugE Methylococcus capsulatus YP_113261
Qac "orfO" class 2 integron AAL51022
EmrE Acinetobacter baumannii YP_001085323
YvaD Geobacillus thermodenitrificans YP_001127717
YvaD Pseudomonas syringae YP_235771
QacE Pyrococcus furiosus AAL81899
YdgE Lyngbya sp. PCC 8106 ZP_01619571
YvaD Herminiimonas arsenicoxydans YP_001101331
YvaE Azoarcus sp. BH72 YP_935092
SugE Verminephrobacter eiseniae YP_999455
YkkD Actinobacillus succinogenes YP_001344922
EmrE Trichodesmium erythraeum YP_720225
YvaD Bacillus subtillis CAB15361
YvaD bacteriophage 82 (N. gonorrheae) YP_208183
60
70
80
90
QacE Archaeoglobus fulgidus NP_070524
QacE Syntrophomonas wolfei YP_752855
QacE Streptococcus thermophilus YP_140506
QacE Streptococcus agalactiae ZP_00785182
EmrE Mannheimia haemolytica EDN74853
EmrE Haemophilus ducreyi NP_874231
EmrE Stenotrophomonas maltophilia ZP_01643607
EmrE Pseudomonas syringae YP_233649
EmrE Pseudomonas aeruginosa NP_253677
EmrE Ralstonia eutropha YP_727122
EmrE Serratia proteamaculans ZP_01534598
EmrE Yersinia intermedia ZP_00834935
EmrE Photorhabdus luminescens NP_929802
EmrE Escherichia coli P23895
EmrE Shigella flexneri YP_689427

Sequence	60 · 70 · 80 · 90
EmrE *Erwinia carotovora* YP_051073	LAH-IPTGIAYAIWSGAGIVLISLLGWLVSG-QKLDLPAILGMGLICA
QacF plasmid pB8 YP_358817	FKF-IPTGIAYAIWAGAGIVLITLMGWIVHK-QTLDLAAMLGMGLIVS
Qac class 3 integron ia-3 ABR28416	LRT-LPTGIAYAIWAGAGIVLITLVGWIIHK-QTLDLPALLGMSLIVS
EmrE *Rhodobacter sphaeroides* YP_353626	LKV-MPTGIVYAVWSGLGIVFIAAIGWIVYG-ERLDLPAILGLGLILL
EmrE *Sulfitobacter sp.* EE36 ZP_00954674	LKV-MPVGIVYAIWSGLGIVCIAGIGYIVFG-QKLDLAAVVGLSMIIG
QacE *Roseobacter denitrificans* YP_682931	LKY-MPVGVVYAIWSGLGIVFIACIGFVVFG-QRLDLPAIIGLGLILS
EmrE *Roseovarius nubinhibens* ZP_00960756	LKV-MPLGVTYALWSGLGICLTVFLGWLVFN-QKVDLPAFLGLSMIVG
EmrE *Magnetospirillum magnetotacticum* ZP_00051262	LKA-MPLGVVYAIWSGVGIVLTAVVGVLVSK-QSLDGPAMVGMAMIVG
EmrE *Methylobacterium chloromethanicum* ZP_02058088	LKA-MPLGVVYAIWSGVGIVLTAVVGVLVFK-QSLDGPAMFGMAMIVC
EmrE *Bradyrhizobium japonicum* NP_770868	IRV-IPLSIAYAIWGGVGIILTATVSFVLFR-QMLDAAAFVGIGLIVS
EmrE *Psychrobacter cryohalolentis* YP_580136	IKT-IPLGIAYALWAGLGIVLTSLVGLVFFK-QTLDTAAVVGIAMIVG
EmrE *Psychrobacter arcticus* YP_264019	LKT-LPLGIAYAMWGGLGIVLTSVIGLVMFK-QHLDTAAVSGITMIVG
EmrE *Paracoccus denitrificans* YP_917819	LRA-MPLGIAYAIWSGLGIVLVSLIGLFLFG-QRLDFAAVIGLTMIVA
EmrE *Rhizobium leguminosarum* YP_769266	LKS-IPVGIAYAIWSALGIVLISSVGLVFFK-QRLDLPAIVGLGLIIS
EmrE *Sinorhizobium meliloti* NP_386521	LRY-IPVGIAYALWSGLGIVLISIAGYVVFG-QKLDLPAILGLALIIA
EmrE *Bordetella pertussis* NP_881103	LRV-IPVGVAYAIWSGVGIVLISLVGALLFKHQHLDLPAIIGIALILA
EmrE *Stappia aggregata* ZP_01546857	IRV-LPTGIVYAIWSGAGIVLITMVAWLVFD-QKLDLPALIGLGLILA
EmrE *Salmonella enterica sbsp. enterica* YP_216634	MRT-IPAGIIYAIWSGVGIVLIGLIGWLFLG-QKLDMPAIIGMLLIIC
EmrE *Salmonella enterica sbsp. enterica* YP_216634	MRT-IPAGIIYAIWSGVGIVLIGLIGWLFLG-QKLDMPAIIGMLLIIC
EmrE *Marinomonas sp.* MWYL1 ZP_01598495	MRS-MPTGVTYAIWSGLGIVLISVFGYLFNS-EKLDLAACIGMSLIVA
EmrE *Flavobacterium johnsoniae* YP_001193927	IRT-IPVGFAYAIWSGVGIVLITAIGAIFFK-EIPDLPAIIGLSLIIL
EmrE *Pseudoalteromonas atlantica* YP_660021	LKT-VPVGIAYAIWAGMGIVLIALFSAIFYK-ELPDLAAIAGMSLILS
EmrE *Acinetobacter baumannii* YP_001085323	LKT-IPMGIAYAIWSGAGIILISAIGWIFYK-QHLDVAACIGLTLMIA
EmrE *Pelobacter propionicus* ABL00914	LRS-IPIGISYAIWSGVGTVLIAGIAAILHR-QLLDIPALVGIGLIVS
EmrE *Stigmatella aurantiaca* ZP_01461726	LRS-IPIGIAYALWSGVGIILVAIAGYVLYQ-QKLDPAAIAGIAMILG
EmrE *Desulfovibrio desulfuricans* YP_388842	TRA-VPIGIAYALWSGFGIVLIAAVNWLIFR-QRLDTPAIIGLGLLIS
EmrE *Photobacterium profundum* ZP_01218031	VKV-VPIGIAYAIWCGAGIVLVSMVSWLWHG-QQLDMFAVLGISLILA
EmrE *Vibrio harveyi* YP_001445675	VKS-MPVGIVYAIWSGAGIVLVAAVGYFLYG-QKLDLAALVGIGFILT
EmrE *Xanthobacter autotrophicus* YP_001417989	LKT-MPVGIAYAIWSGVGIVLVTAIAWLWYG-QRLDLPALIGLGLIIA
EmrE *Sphingomonas wittichii* YP_001263498	LRT-MPTGVAYAIWSGVGLVLITTVAWLFQG-QKLDIAALIGMALIVA
EmrE *Acidovorax avenae subsp. citrulli* YP_973010	LQA-IPTGIAYAIWSGVGIVLVSTVGWLVYG-QRLDGPALAGMGLILA
EmrE *Delftia acidovorans* ZP_01577698	LRT-VPTGVAYAIWSGAGIVLISLVGWLWQG-QTLDLPALAGMGLIVA
EmrE *Myxococcus xanthus* YP_633339	LRT-IPTGIAYAIWSGAGIVLVSGVAWLLHG-QRLDVPALVGIGLILA
EmrE *Dechloromonas aromatica* YP_283677	QNL-MPVGIIYAIWSGVGIVLVSLVAVFVYG-QVLDLAAMLGIALIVA
QacE *Xanthomonas campestris* NP_637776	LRV-IPVGIAYALCSGAGIVLISLVAWLVHG-QRLDLPAMLGRALIVA
EmrE *Burkholderia cepacia* YP_773352	LRT-MPVGIIYAVWSGAGIVLITLVAMLLYR-QVPDVPAVIGLGLIVA
Qac intI1 (*Serratia marcescens*) AAK40353	LKS-IPVGIAYAVWAGLGIVLVAAIAWIFHG-QKLDFWAFIGIGLIVS
QacF intI (*Achromobacter denitrificans*) AAZ14837	LKS-IPVGIAYAVWAGLGIVLVAAIAWIFHG-QKLDFWAFIGIGLIVS
QacF plasmid pIP833 (*E. aerogenes*) AAD22143	LKS-IPVGIAYAVWAGLGIVLVAAIAWIFHG-QKLDFWAFIGMGLIVS
QacH plasmid p3iANG (*Vibrio cholerae*) AAZ42322	LKS-IPVGIAYAVWAGLGIVLVAAIAWIFHG-QKLDFWAFIGMGLIVS
QacF plasmid pSC138 (*S. enterica*) AAX56371	LKS-IPVGIAYAVWAGLGIVLVAAIAWIFHG-QKLDLWAFVGMGLIVS
QacE2 Int Class1 (*A. salmonicida*) AAK53557	LKS-IPVAIAYAVWSGLGIVLVTAIAWVLHG-QKLDMWGFVGVGFIIS
QacG class I intergon (*A. baumannii*) AAL38576	LKS-IPVGIAYAVWSGLGIVLVTAIAWVLHG-QKLDMWGFVGVGFIIS
QacG integron (*P. aeruginosa*) AAS79147	LKS-IPVGIAYAVWSGLGIVLVTAIAWVLHG-QKLDMWGFVGVGFIIS
QacE plasmid R751(*E. aerogenes*) NP_044260	LKS-IPVGVAYAVWSGLGVVIITAIAWLLHG-QKLDAWGFVGMGLIVS
QacEΔ1 class 2 integron (*P. aeruginosa*) AAV32841	LKS-IPVGVAYAVWSGLGVVIITAIAWLLHG-QKLDAWGFVGMGLIIA
EmrE *Marinobacter aquaeolei* YP_958340	IKV-IPVGIAYAVWAGLGIVLISLIGWLLLG-QKLDFPAVVGMLLIVA
Smr *Chloroflexus aurantiacus* ZP_00766077	LEA-IPVGIAYAVWSGIGIVLITVAAWFLYG-QRLDVWALIGIGFIIV
EmrE *Shewanella frigidimarina* YP_748773	LKG-IPVGIAYATWAGLGIVLITAIAWVMYG-QKLDLGALVGMTFILV
EmrE *Chromobacterium violaceum* NP_902350	LRH-IPVGIAYALWSGIGIVLVSLIAWLLYG-QKLDLAAVAGMGLIIA
EmrE *Methylobacillus flagellatus* YP_544992	LRS-IPVGVAYAVWSGLGIVLVSVIASLLYG-QKLDLPAVIGMALIIA
EmrE *Methylococcus capsulatus* YP_115175	VDK-IPVGISYAIWSALGIVLVSLIAWILHG-QALDRPAIAGMGLIIA
EmrE *Coxiella burnetii* ZP_01299513	LKS-IPVGLAYAIWAGLGIVLISIIGWVVFH-QHLDIAAIVGLVLIIS
QacH *Brucella melitensis* YP_414370	LKT-IPVGVAYAIWSGVGVTLVALIGWLVFG-QKLDLPAIVGMGLIIA
QacE *Brucella melitensis* AAL52226	LKT-IPVGVAYAIWSGVGVTLVALIGWLVFG-QKLDLPAIVGMGLIIA
EmrE *Ochrobactrum anthropi* YP_001370804	LKS-IPVGIAYAVWSGIGVTLVALIGWLVFG-QKLDLAAVLGMGLIIA
EmrE *Marinobacter algicola* ZP_01894487	LKT-LPVGVAYAVWAGAGVALIALAGYLFFG-QTLDLPAILGIGLIVA
EmrE *Nitrosococcus oceani* YP_342650	LRT-LPVGVAYAVWSGVGVALITLVAWLFYG-QTLDTPALLGLALIIA
EmrE *Reinekea sp.* MED297 ZP_01116498	LKT-IPVGVAYAIWSGLGVALVALLSWWFYG-QAFDLAGVIGVCLIVS

	60 70 80 90
EmrE *Marinobacter algicola* ZP_01894487	LKA-IPVGIAYAIWSGLGTALVAVVAWVFMG-QRLDLPAVFGILLLIIA
EmrE *Nitrosococcus oceani* YP_342650	LRY-FPVGIVYAIWSGVGVALVTLAGWFFYH-QGLDAGAIAGIVLIVI
EmrE *Reinekea sp.* MED297 ZP_01116498	RT--IPMGIAYAVWAGVGIVLIALVGAVFFK-QLPDVPAMVGMGCIIT
EmrE *Geobacter sulfurreducens* NP_951765	R--TIPLGVAYAVWAGVGIVTVTLIGVYWFNQPI-GAGQAAGIAMILG
EmrE *Chlorobium limicola* EAM42528	RD--LPVGIAYAFWAGLGIVLVTLIGIVVYG-EKPDLPALLGLGLIIA
EmrE *Alcanivorax borkumensis* YP_693512	KN--IPMGITYASFAGLAIISTVGVGIIKYNQVP-NLYSIVGLCFIIV
EmrE *Jannaschia sp. CCS1* YP_511013	TI--IPVGFTYATYGGLTITAVTIFGVVKYNQTP-NLYAVLGISLIII
EmrE *Chromohalobacter salexigens*	KG--MPIGIVHALWAGLAIVTVTLLSTVIYRQHL-DLTTWVGMAFVAV
QacH *Pelagibacter ubique* ZP_01264056	KG--MPVGIVHAMWAGLAIVTVTLLSTLVYKQHL-DLSTWLGMALVAI
QacH2 *Pelagibacter ubique* YP_266208	KG--MPVGVVHALWAGMAIVTVTLLSALFYRQHL-DMTAWIGMLLVAL
EmrE *Pseudoalteromonas atlantica* YP_661014	KG--MPVGVVHALWAGMAIVTVTLLSALFYRQHL-DMTAWIGMLLVAL
QacF *Pseudoalteromonas haloplanktis* CAI87270	KG--MPVGVVHALWAGLAIVSVTLISALFYREHL-DMTAWIGMVLVAA
Qac megaplasmid (*S. oneidensis)* NP_720475	KG-GMPVGIVHALWAGLAIVSVTLISQLVYRQHM-DTSLWIGMALIAA
YvaE *Shewanella frigidimarina* YP_752098	KN--LPVGIVHASWAGLAIVLVTLMSNIIYKQHL-DAKVWLGMVVIGI
EmrE *Shewanella pealeana* YP_001500134	RD--LPMSLITSMWSGLGITLITVIAAFRYQQVP-TLMAIGGISLIIA
EmrE *Vibrio harveyi* YP_001449011	KD--IPIGIAYSSWSGFGIVFVTISGYFLYNQKI-NLIEIFGIILIIT
EmrE *Photobacterium profundum* ZP_01219483	ST--IPVGVAYSLWCGFGIVGVTICSMILYKQKP-DLPAILAMALIIS
EbrB *Pseudomonas syringae* YP_235623	GY--LPVGIAYSLWCGFGIIGVTFISMLLYKQKP-DLPAVFSMVLIIS
EmrE *Wigglesworthia glossinidia* NP_871589	KD--IPVGLAYATWSGTGILVVSTLGMAFYGQHP-DTAAMIGMAVIAS
EmrE *Aeromonas hydrophila* YP_855864	KD--IPVGLAYATWSGTGILMVSTLGILFYGQHP-DTAAIIGMVIIAS
EmrE *Erwinia carotovora* YP_051860	RH--LPVGLAYATWSGTGILLVSTLSIIFYGQHP-DIPAIVGMIVIAT
EmrE *Aeromonas hydrophila* YP_855865	TR--IPVGVAYAIWSGAGVVLISLIGAIFLR-QSPSMIQILFISLIVV
QacE integron (*Escherichia coli*) YP_672432	QS--IPLGLTYALWSGIGIVAIVLVGVLAYR-QVPSSGQLIGMGLIAA
QacH *Erwinia carotovora* YP_051859	LKS-IPLGMAYAIWSGVGLVLTAIVGVVVFG-EKVDFWGIASIGLILA
EmrE *Nitrobacter winogradskyi* YP_319204	LKT-MPLATAYAIWAGVGLVLTALVSVVFFG-EKADFIGIVSIGLILL
EmrE *Synechococcus sp.* RS9917 ZP_01081426	LKT-LPLSLAYAIWSGVGTALTALIGVLVWN-EPFNILTFIGLVMIVG
EbrB *Mannheimia haemolytica* EDN74090	LRY-LSMSLAYATWAGAGTALTALISVVVFR-ESLNVIAVFGLLFIIG
EmrE *Neisseria meningitidis* NP_284788	LKT-IDLSSAYATWSGVGTALTAIVGFLLFG-ETISLKGVFGLTLVIA
EbrB *Listeria monocytogenes* EAL09800	LTY-LPLNLAYATWAGLGTSLTVLVDYFFFK-ESLTKRSLLGLVLIVC
EbrA *Paenibacillus larvae* ZP_02326984	LNH-IPLSLSYATWSGAGTVLTTVIGVKWFK-EDLNAKGLIGILLLLS
EbrB *Bacillus subtillis* CAB13613	IKF-LPLNITYATWAGLGLVLTTIISVIVFK-ENVNLISIISIGLIVI
EbrB *Paenibacillus larvae* ZP_02326985	MKY-LPLNVSYATWAGLGLVLTTIVSVVIFK-ESVNLISIFSIILIII
EbrA *Bacillus subtillis* CAB13614	MQH-LPLNITYASWAGLGLVLTTIVSVLIFK-EQINLISIISIILIIF
QacG plasmid pST94 O87866	MQH-LPLNITYATWAGLGLVLTTVVSIIIFK-EQINLITIVSIVLIIV
QacJ plasmid pNVH01 (*S. aureus*) CAD55144	LKT-IPLNLAYAIWSGLGTIATVIISILIWQ-ERVNIASITGISLIIV
QacH plasmid pST2H6 (*S. saprophyticus*) CAA76544	VKN-IPINIAYAIWSGVGIILMTVISVFVFH-NPINVPTVLGILFITL
Smr *Staphylococcus aureus* AAM94142	LAR-LPLAIAYSIWCGLGMSVVTLTGILVYK-EKFGWKVALGLLLIFS
EbrA *Desulfotomaculum reducens* YP_001114614	LEH-IPLGTAYAIWSGGGTALTAIVGILVWK-EKFNLKILLGLLIIIA
Ebr *Pediococcus pentosaceus* YP_804449	LRT-IPLGVAYAIWSGLGTAAIAVIGVLVWR-EQLNITGVIGIVLIIA
Ebr1 *Synechococcus sp.* RS9917 ZP_01080450	LRT-IPLGIAYAIWSGLGTAAIAVIGVIVWR-EQLNTAGVIGIVLIII
EbrA *Listeria monocytogenes* EAL09801	LKA-MPLSVVYAIWSGVGTAATAFIGVVLFR-EVLDAPRLIGIALIIV
Smr *Roseiflexus castenholzii* ZP_01530339	LKT-VPLSLAYAVWAGVGTAAIALIGAFFFG-EAISPKGWLGIALVAV
EbrA *Roseiflexus sp.* RS-1 YP_001278866	EE--LPVGVVYGTWAAVGIVATALVGVVVFE-ESVDVAGVVGLALIVA
EbrA *Gloeobacter violaceus* NP_927270	KT--LPLGIVYAVWAGGGLAIMTIVSQWIFN-ETLDLRQIGCIALILI
EbrB *Thermus thermophilus* YP_004540	KR-GMGLGVAYGIWGATGVALTAVLSSVMFG-EALTAVMGVGLSCIIA
EbrB *Halobacterium sp.* NRC1 NP_444228	RR-GMGLGVAYGIWGASGVAMTALLSAVLFG-EELTALMIAGLGCIIA
EmrE *Zymomonas mobilis sbsp. mobilis* YP_161843	KA-GMAVGVAYGIWGASGVALTAVGATVLFG-QPLTGLMILGLVLIVA
Smr2 *Mycobacterium vanbaalenii* YP_954149	RQ-GMPLGIAYGIWAAAGVALTALASHVLFA-EPLTRRMVGGIALIAA
Smr2 *Mycobacterium gilvum* YP_001134816	RH-GMPLGVAYGIWAAAGVALTAAFSRLLFD-EPLTRKMLGGIALIMV
EbrB *Clavibacter michiganensis* YP_001220961	SL-GLPIGVAYGIWAATGVALTAILGRVLFR-DPLTRTMLAGIALIIG
Smr *Nocardia farcinica* YP_118186	KR-GMAIGVAYGIWSAVGVAAIALIGVLFLN-ERLTLVQVGGIGLVIL
Smr *Saccharopolyspora erythraea* YP_001109297	KL-GLPVGVVYAIWAGAGVALVAVIGAVFLG-ETITPVQIGGVVLIVG
Smr *Thermobifida fusca* YP_289048	KL-GMGVGVAYGIWSALGVTLVAVIGAVFLG-DTLTWVQIVGIVLVIA
Smr *Saccharopolyspora erythraea* YP_001103712	KQ-GMDMGVAYGIWAALGVTAVALVGAAFLG-DTLTWAQMIGIVLVIG
Smr *Nocardioides sp*. JS614 YP_921842	QR-GLNVAIVYALWSATGIVAIAVIGATFLG-ERLTSAQVLGMGLIVS
Smr *Nocardioides sp*. JS614 YP_921842	SHG-MQVGIAYALWSAIGTTVIVAVGVTFLG-EPISTLKVIGVALVVS
Smr *Mycobacterium tuberculosis* P95094	SHG-MQTDVAYALWSAIGTAAIVLVAVLFLG-SPISVMKVVGVGLIVV
NepA plasmid pAO1 (*A. nicotinovorans*) CAD47919	KF--TDIGIAYALWAGLGTASVAVIGVLFRN-ERFSWKHAIGLALVVT

Sequence	Alignment (positions ~55–97)
EmrE *Candidatus Pelagibacter ubique* ZP_01264207	DK--LPIAIVYATWSGLGIFTIAILGYIFFK-QSLSWQAVLGLFFIVT
QacH *Lactobacillus plantarum* NP_786542	QR--LPLGVTYAMWAGLGTFATVILAVVVYR-ERLSLSRLSGLIAIVV
YdgF *Escherichia coli* ABE07266	K--KIALGVAYALWEGIGILFITLFSVLLFDESL-SLMKIAGLTTLVA
YdgF *Shigella flexneri* YP_689093	K--KIALGVAYALWEGIGILFITLFSVLLFDESL-SLMKIAGLTTLVA
YdgF *Salmonella enterica sbsp. enterica* YP_216487	K--KIALGVAYALWEGIGILFITIFSVLLFDEAL-STMKIAGLLTLVA
YdgF *Yersinia intermedia* ZP_00833156	K--KVALGVAYALWEGIGILIITVFSVLWFDESL-SPLKIAGLVTLIG
YdgF *Photorhabdus luminescens* NP_929383	K--KVALGVAYALWEGVGILFITVFSVMWFDESL-SLMKVGGLALLIT
YdgF *Pseudomonas aeruginosa* NP_250232	K--RVPVGVAYALWEGIGIVLITAVSVAWLGESI-GLYKAVGLGVMIA
YdgF *Sinorhizobium meliloti* NP_385720	K--TIPVGVAYAVWEGSGVAVITLVSVFVFGHAL-SGREMLGLAMAVA
YdgF *Aeromonas hydrophila* YP_857997	K--TISIGIAFALWEGLGIALITVVSVLFLDYHL-NAQELIGLALAIV
YdgF *Vibrio harveyi*	K--KISIGVAYATWEGLGIALITLVSIVLFDANL-STQQLLGLALAVV
YdgF *Chromobacterium violaceum* NP_900949	R--AISVGVAYAVWEGLGLVGLTAVSVLLFGEKL-VPQEIVGLGLALL
YdgF *Campylobacter jejuni* YP_179294	K--KIQVGIAYAVWELLGIILILLVSFIVFKESL-TLTQILGIILSIV
YdgF *Desulfovibrio desulfuricans* YP_388898	T--ALPVGVAFAFWEGVGLAFITLASVFILGEQM-SLLRFAALCAVLG
YdgF *Lawsonia intracellularis* CAJ54979	T--GLPIGVAYAFWEGLGLILITLVSIFVIDEKM-TITRLISLFALLS
YdgF *Deinococcus geothermalis* YP_605419	R--RIPVAVAFAVWEAVGLAAVTLLSVWLLGDHL-TQLQLLALGGLLL
YdgF *Pseudoalteromonas haloplanktis* YP_340952	S--KIPIALANAFWEGLGMILIASVSFVFLGEAI-SIGQMFALLLAII
YdgF *Acidovorax avenae subsp. citrulli* YP_969092	K--QIPMAVAYATWETLGLLAIAFIGYRYFGESM-SAGKLLGMSVLIV
YdgF *Helicobacter hepaticus* NP_860040	K--RISVGVAYAIWEVLGAICVVCISVFYFGEEI-SFIQKIGIVLSIS
EmrE *Alteromonas macleodii* ZP_01110205	K--NMGIGVAYAIWSGMGIALITAASVVFWKQTF-DIYAVLGIMLIIS
YvaE *Bacillus subtilis* CAB15362	KS--IDVSVAYAVWSGMGIVLITVVGFLFFQ-EHVSVMKVISIGLIIA
YvaE *Blastopirellula marina* ZP_01094237	NK--IDVSHAYAVWSGVGTALIAVIGICFFR-ETFSPVKLISILLIIA
YvaE *Desulfitobacterium hafniense* YP_517233	KK--IPVSVAYAVWSGVGIVVISAIGIAVFK-ETVTTLKVVSIVLIVA
YvaE *Rhodospirillum rubrum* YP_428434	KK--MDVSIAYAIWSGAGTVLITVIGILVFK-EPATLLKVLCIALIVA
YvaE *Bdellovibrio bacteriovorus* NP_968626	KY--LPVSNVYAIWAGVGTALMAFLGLVIFN-EPLPLQKVVATTLIIA
Smr *Streptomyces coelicolor* AAK95484	KI--LAVGTAYAIWAGVGTAAIATIGVAFMG-EGMTLTKAAGIALIII
YvaE *Acidobacteria bacterium* YP_593828	KE--LEVGAVYAIWSALGTVAVALLGMVLFH-EPANALKVFSIVLIIV
EbrB *Rubrobacter xylanophilus* YP_644095	RR--MDVGVAYAVWSGLGTALVAIVGIALFG-EEMTAARALALALIIA
YvaE *Desulfovibrio vulgaris* YP_966624	KK--MDVSIAYAVWSGVGTATVAMIGICIFG-ERAT-VKICSLLLIII
YvaE *Pelodictyon phaeoclathratiforme* ZP_00590439	KV--LPIALTYAIWSGIGTAAITVIGVIWFG-EGINAIKLASLLLIII
YvaE *Chlorobium tepidum* NP_662342	RT--LPVGMSYAIWSALGTALITAIGVLWFG-EGLNALKIISLILIIA
YvaE *Prosthecochloris vibrioformis* YP_001130806	KV--LPIGMTYAIWAGLGTAVVSMIGVIWFA-ETLTPVKLFSLALIII
YvaE *Anabaena variabilis* YP_323581	KN--IDISIAYSVWAGLGTALIAGVGLIWFR-ESMTLVKFISMTLIIV
YvaE *Nostoc sp.* PCC 7120 NP_484329	KN--IDISIAYSVWAGLGTALIAGVGLIWFR-ESMTMVKFISMTLIIV
EbrA *Methanosarcina acetivorans* NP_616284	KG--IDVSVAYAIWAGLGTALITIVGILWFR-EPATALKMISLIVVIT
YvaE *Methanococcoides burtonii* YP_566929	KN--IDVSLAYAIWSGVGTASMTLIGVYYFK-EPATTIKMVSIFIVMA
YvaE *Clostridium beijerinckii* YP_001307448	KK--VQIGVAYATWSGIGIILLSAIGIVFFK-ESINLQKTVFIGLIII
YvaE *Marinomonas sp.* MED121 ZP_01075592	KT--LEISIAYAVWSGAGIVIITFIGTIWFA-EQFSLAKFAFISLILI
QacC *Lactobacillus plantarum* NP_786541	SK--IPLSVGYAIWAGVGTAATGLIGIWAFG-EILTGLKTLGFMAIIT
NepB plasmid pAO1 (*A. nicotinovorans*) CAD47918	RA--VPLSVAYATWSGLGTVAVAFAGAIIHG-EAVTLGRITAITAVIG
YvaE *Burkholderia cenocepacia* YP_839289	L--NVEVGLAYAAWAGASTAITAGLGILLFGESV-SVSKLCGLAMAAG
EmrE *Synechococcus sp.* RS9917 ZP_01080451	R--VLPMGFAYALWVGVGMVVASVSGVFLFSEAL-TPSVFLGLMFVFV
SugE *Escherichia coli* AAC46453	KS--LPVGTAYAVWTGIGAVGAAITGIVLLGESA-NPMRLASLALIVL
SugE *Shigella flexneri* YP_691592	KS--LPVGTAYAVWTGIGAVGAAITGIVLLGESA-NPMRLASLALIVL
SugE *Salmonella enterica sbsp. enterica* YP_219204	KT--LPVGTAYAIWTGIGAVGAAITGILLLGESA-SPARLLSLGLIVA
SugE plasmid/ *Citrobacter freundii* AAC46457	RT--LPVGTAYAVWTGIGAVGAAITGILLLGESA-SPARLLSLGLIVA
SugE *Serratia proteamaculans* ZP_01538212	KT--LPAGTAYAVWTGIGAVGAAIMGMVLLGEST-NIARILSLCLIVV
SugE *Yersinia intermedia* ZP_00832613	KT--LPAGTAYAVWTGIGAIGTAILGIALLGESA-SLARVLSLGLILA
SugE *Photorhabdus luminescens* NP_931319	KG--LPIGTAYAVWTGIGAVGTAIFGIIVFGESA-SFARILSFALIIA
SugE *Proteus vulgaris* P20928	KG--LPAGTAYAIWTGIGAVGTAIFGIIVFGESA-NIYRLLSLAMIVF
SugE *Pseudoalteromonas atlantica* YP_660982	KS--LPAGTAYAVWVVIGLIGTAIVGIVMLNEPL-NFWRVVSLITITV
SugE *Limnobacter sp.* MED105 ZP_01915318	KT--LPVGTAYSVWVGIGAAGTVLMGVVLFNEPV-NALRIGSVVLIVL
SugE *Shewanella oneidensis* NP_717508	KQ--LPIGTAYGVWVGIGAMGTAIAGIILLGEGV-SLLKIASLILILL
SugE *Methylobacillus flagellatus* YP_545743	RS--LPMGTAYAIWVGIGAVGTVIMGIFLFNEPT-SLVRLFSVALIIV
SugE *Bradyrhizobium japonicum* NP_770684	KS--LPVGTAYAVWTGIGAVGTATLGIILFGEPA-TALRLASIGLIVA
SugE *Ralstonia eutropha* YP_727054	RH--IPVGTGYAVWTGIGAVGTAILGIILFNEPA-TAARLACIGLIVC
SugE *Dechloromonas aromatica* YP_287101	KT--LPLGTAYAVWTGIGAVGTAILGIYLFDESR-EVLRFVCIGLIVA
SugE *Mesorhizobium loti* NP_105639	KA--LPVGTAYAVWTGIGTVGTALLGIWLLGEPA-TAIRLACIALIVC

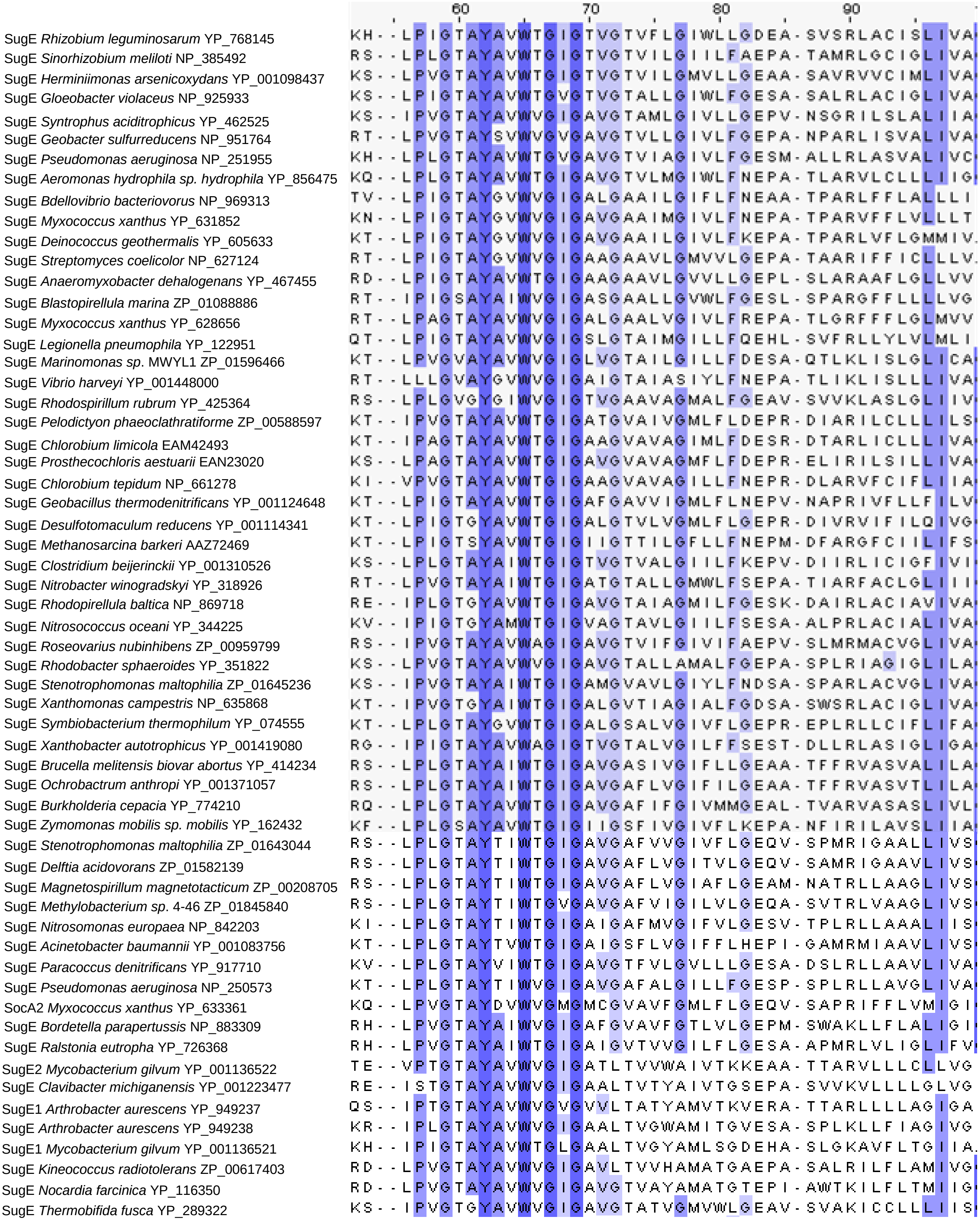
60 70 80 90
SugE Rhizobium leguminosarum YP_768145 KH--LPIGTAYAVWTGIGTVGTVFLGIWLLGDEA-SVSRLACISLIVA
SugE Sinorhizobium meliloti NP_385492 RS--LPLGTAYAVWTGIGTVGTVILGIILFAEPA-TAMRLGCIGLIVA
SugE Herminiimonas arsenicoxydans YP_001098437 KS--LPVGTAYAVWTGIGTVGTVILGMVLLGEAA-SAVRVVCIMLIVA
SugE Gloeobacter violaceus NP_925933 KS--LPIGTAYAVWTGVGTVGTALLGIWLFGESA-SALRLACIGLIVA
SugE Syntrophus aciditrophicus YP_462525 KS--IPVGTAYAVWVGIGAVGTAMLGIVLLGEPV-NSGRILSLALIIA
SugE Geobacter sulfurreducens NP_951764 RT--LPVGTAYSVWVGVGAVGTVLLGIVLFGEPA-NPARLISVALIVA
SugE Pseudomonas aeruginosa NP_251955 KH--LPLGTAYAVWTGVGAVGTVIAGIVLFGESM-ALLRLASVALIVC
SugE Aeromonas hydrophila sp. hydrophila YP_856475 KQ--LPLGTAYAVWTGIGAVGTVLMGIWLFNEPA-TLARVLCLLLIIG
SugE Bdellovibrio bacteriovorus NP_969313 TV--LPIGTAYGVWVGIGALGAAILGIFLFNEAA-TPARLFFLALLLI
SugE Myxococcus xanthus YP_631852 KN--LPIGTAYGVWVGIGAVGAAIMGIVLFNEPA-TPARVFFLVLLLT
SugE Deinococcus geothermalis YP_605633 KT--LPIGTAYGVWVGIGAVGAAILGIVLFKEPA-TPARLVFLGMMIV.
SugE Streptomyces coelicolor NP_627124 RT--LPIGTAYGVWVGIGAAGAAVLGMVVLGEPA-TAARIFFICLLLV.
SugE Anaeromyxobacter dehalogenans YP_467455 RD--LPIGTAYAVWTGIGAAGAAVLGVVLLGEPL-SLARAAFLGLLVV
SugE Blastopirellula marina ZP_01088886 RT--IPIGSAYAIWVGIGASGAALLGVWLFGESL-SPARGFFLLLLVG
SugE Myxococcus xanthus YP_628656 RT--LPAGTAYAVWVGIGALGAALVGIVLFREPA-TLGRFFFLGLMVV
SugE Legionella pneumophila YP_122951 QT--LPIGTAYAVWVGIGSLGTAIMGILLFQEHL-SVFRLLYLVLMLI
SugE Marinomonas sp. MWYL1 ZP_01596466 KT--LPVGVAYAVWVGIGLVGTAILGILLFDESA-QTLKLISLGLICA
SugE Vibrio harveyi YP_001448000 RT--LLLGVAYGVWVGIGAIGTAIASIYLFNEPA-TLIKLISLLLIVA
SugE Rhodospirillum rubrum YP_425364 RS--LPLGVGYGIWVGIGTVGAAVAGMALFGEAV-SVVKLASLGLIIV
SugE Pelodictyon phaeoclathratiforme ZP_00588597 KT--IPVGTAYAVWTGIGATGVAIVGMLFLDEPR-DIARILCLLLILS
SugE Chlorobium limicola EAM42493 KT--IPAGTAYAVWTGIGAAGVAVAGIMLFDESR-DTARLICLLLIVA
SugE Prosthecochloris aestuarii EAN23020 KS--LPAGTAYAVWTGIGAVGVAVAGMFLFDEPR-ELIRILSILLIVA
SugE Chlorobium tepidum NP_661278 KI--VPVGTAYAVWTGIGAAGVAVAGILLFNEPR-DLARVFCIFLIIA
SugE Geobacillus thermodenitrificans YP_001124648 KT--LPIGTAYAVWTGIGAFGAVVIGMLFLNEPV-NAPRIVFLLFILV
SugE Desulfotomaculum reducens YP_001114341 KT--LPIGTGYAVWTGIGALGTVLVGMLFLGEPR-DIVRVIFILQIVG
SugE Methanosarcina barkeri AAZ72469 KT--LPIGTSYAVWTGIGIIGTTILGFLLFNEPM-DFARGFCIILIFS
SugE Clostridium beijerinckii YP_001310526 KS--LPLGTAYAIWTGIGTVGTVALGIILFKEPV-DIIRLICIGFIVI
SugE Nitrobacter winogradskyi YP_318926 RT--LPVGTAYAIWTGIGATGTALLGMWLFSEPA-TIARFACLGLIII
SugE Rhodopirellula baltica NP_869718 RE--IPLGTGYAVWTGIGAVGTAIAGMILFGESK-DAIRLACIAVIVA
SugE Nitrosococcus oceani YP_344225 KV--IPIGTGYAMWTGIGVAGTAVLGIILFSESA-ALPRLACIALIVA
SugE Roseovarius nubinhibens ZP_00959799 RS--IPVGTAYAVWAGIGAVGTVIFGIVIFAEPV-SLMRMACVGLIVA
SugE Rhodobacter sphaeroides YP_351822 KS--LPVGTAYAVWVGIGAVGTALLAMALFGEPA-SPLRIAGIGLILA
SugE Stenotrophomonas maltophilia ZP_01645236 KS--IPVGTAYAIWTGIGAMGVAVLGIYLFNDSA-SPARLACVGLIVA
SugE Xanthomonas campestris NP_635868 KT--IPVGTGYAIWTGIGALGVTIAGIALFGDSA-SWSRLACIGLIVA
SugE Symbiobacterium thermophilum YP_074555 KT--LPLGTAYGVWTGIGALGSALVGIVFLGEPR-EPLRLLCIFLIFA
SugE Xanthobacter autotrophicus YP_001419080 RG--IPIGTAYAVWAGIGTVGTALVGILFFSEST-DLLRLASIGLIGA
SugE Brucella melitensis biovar abortus YP_414234 RS--LPLGTAYAIWTGIGAVGASIVGIFLLGEAA-TFFRVASVALILA
SugE Ochrobactrum anthropi YP_001371057 RS--LPLGTAYAIWTGIGAVGAFLVGIFILGEAA-TFFRVASVTLILA
SugE Burkholderia cepacia YP_774210 RQ--LPLGTAYAVWTGIGAVGAFIFGIVMMGEAL-TVARVASASLIVL
SugE Zymomonas mobilis sp. mobilis YP_162432 KF--LPLGSAYAVWTGIGIIGSFIVGIVFLKEPA-NFIRILAVSLIIA
SugE Stenotrophomonas maltophilia ZP_01643044 RS--LPLGTAYTIWTGIGAVGAFVVGIVFLGEQV-SPMRIGAALLIVS
SugE Delftia acidovorans ZP_01582139 RS--LPLGTAYTIWTGIGAVGAFLVGITVLGEQV-SAMRIGAAVLIVS
SugE Magnetospirillum magnetotacticum ZP_00208705 RS--LPLGTAYTIWTGIGAVGAFLVGIAFLGEAM-NATRLLAAGLIVS
SugE Methylobacterium sp. 4-46 ZP_01845840 RS--LPLGTAYTIWTGVGAVGAFVIGILVLGEQA-SVTRLVAAGLIVS
SugE Nitrosomonas europaea NP_842203 KI--LPLGTAYTIWTGIGAIGAFMVGIFVLGESV-TPLRLLAAALIIS
SugE Acinetobacter baumannii YP_001083756 KT--LPLGTAYTVWTGIGAIGSFLVGIFFLHEPI-GAMRMIAAVLIVS
SugE Paracoccus denitrificans YP_917710 KV--LPLGTAYVIWTGIGAVGTFVLGVLLLGESA-DSLRLLAAVLIVA
SugE Pseudomonas aeruginosa NP_250573 KT--LPLGTAYTIWVGIGAVGAFALGILLFGESP-SPLRLLAVGLIVA
SocA2 Myxococcus xanthus YP_633361 KQ--LPVGTAYDVWVGMGMCGVAVFGMLFLGEQV-SAPRIFFLVMIGI
SugE Bordetella parapertussis NP_883309 RH--LPVGTAYAIWVGIGAFGVAVFGTLVLGEPM-SWAKLLFLALIGI
SugE Ralstonia eutropha YP_726368 RH--LPVGTAYAIWTGIGAIGVTVVGILFLGESA-APMRLVLIGLIFV
SugE2 Mycobacterium gilvum YP_001136522 TE--VPTGTAYAVWVGIGATLTVVWAIVTKKEAA-TTARVLLLCLLVG
SugE Clavibacter michiganensis YP_001223477 RE--ISTGTAYAVWVGIGAALTVTYAIVTGSEPA-SVVKVLLLLGLVG
SugE1 Arthrobacter aurescens YP_949237 QS--IPTGTAYAVWVGVGVVLTATYAMVTKVERA-TTARLLLLAGIGA
SugE Arthrobacter aurescens YP_949238 KR--IPLGTAYAVWVGIGAALTVGWAMITGVESA-SPLKLLFIAGIVG
SugE1 Mycobacterium gilvum YP_001136521 KH--IPIGTAYAVWTGLGAALTVGYAMLSGDEHA-SLGKAVFLTGIIA.
SugE Kineococcus radiotolerans ZP_00617403 RD--LPVGTAYAVWVGIGAVLTVVHAMATGAEPA-SALRILFLAMIVG
SugE Nocardia farcinica YP_116350 RD--LPVGTAYAVWVGIGAVGTVAYAMATGTEPI-AWTKILFLTMIIG
SugE Thermobifida fusca YP_289322 KS--IPVGTGYAVWVGIGAVGTATVGMVWLGEAV-SVAKICCLLLIIS

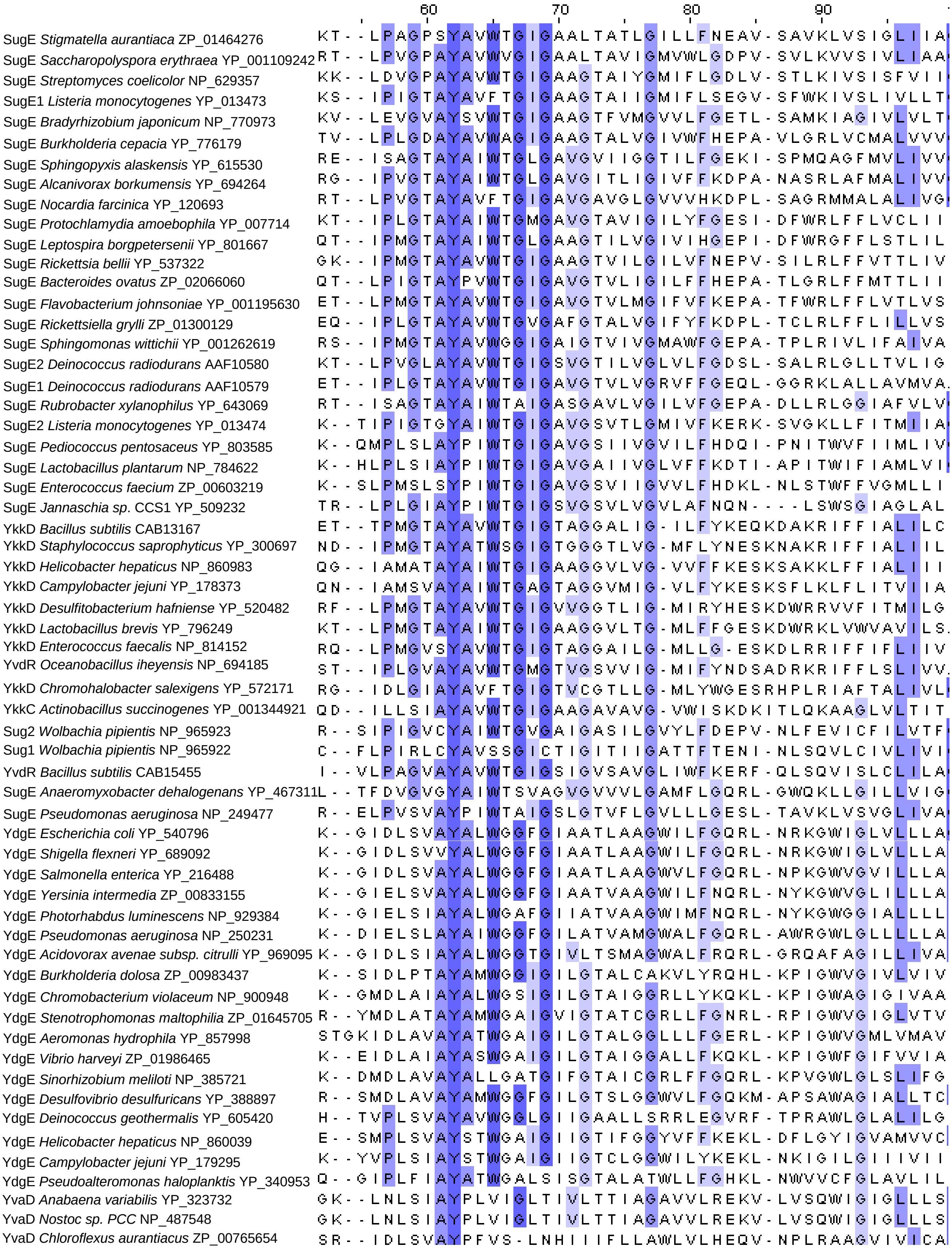
60
70
80
90
SugE Stigmatella aurantiaca ZP_01464276
SugE Saccharopolyspora erythraea YP_001109242
SugE Streptomyces coelicolor NP_629357
SugE1 Listeria monocytogenes YP_013473
SugE Bradyrhizobium japonicum NP_770973
SugE Burkholderia cepacia YP_776179
SugE Sphingopyxis alaskensis YP_615530
SugE Alcanivorax borkumensis YP_694264
SugE Nocardia farcinica YP_120693
SugE Protochlamydia amoebophila YP_007714
SugE Leptospira borgpetersenii YP_801667
SugE Rickettsia bellii YP_537322
SugE Bacteroides ovatus ZP_02066060
SugE Flavobacterium johnsoniae YP_001195630
SugE Rickettsiella grylli ZP_01300129
SugE Sphingomonas wittichii YP_001262619
SugE2 Deinococcus radiodurans AAF10580
SugE1 Deinococcus radiodurans AAF10579
SugE Rubrobacter xylanophilus YP_643069
SugE2 Listeria monocytogenes YP_013474
SugE Pediococcus pentosaceus YP_803585
SugE Lactobacillus plantarum NP_784622
SugE Enterococcus faecium ZP_00603219
SugE Jannaschia sp. CCS1 YP_509232
YkkD Bacillus subtilis CAB13167
YkkD Staphylococcus saprophyticus YP_300697
YkkD Helicobacter hepaticus NP_860983
YkkD Campylobacter jejuni YP_178373
YkkD Desulfitobacterium hafniense YP_520482
YkkD Lactobacillus brevis YP_796249
YkkD Enterococcus faecalis NP_814152
YvdR Oceanobacillus iheyensis NP_694185
YkkD Chromohalobacter salexigens YP_572171
YkkC Actinobacillus succinogenes YP_001344921
Sug2 Wolbachia pipientis NP_965923
Sug1 Wolbachia pipientis NP_965922
YvdR Bacillus subtilis CAB15455
SugE Anaeromyxobacter dehalogenans YP_467311
SugE Pseudomonas aeruginosa NP_249477
YdgE Escherichia coli YP_540796
YdgE Shigella flexneri YP_689092
YdgE Salmonella enterica YP_216488
YdgE Yersinia intermedia ZP_00833155
YdgE Photorhabdus luminescens NP_929384
YdgE Pseudomonas aeruginosa NP_250231
YdgE Acidovorax avenae subsp. citrulli YP_969095
YdgE Burkholderia dolosa ZP_00983437
YdgE Chromobacterium violaceum NP_900948
YdgE Stenotrophomonas maltophilia ZP_01645705
YdgE Aeromonas hydrophila YP_857998
YdgE Vibrio harveyi ZP_01986465
YdgE Sinorhizobium meliloti NP_385721
YdgE Desulfovibrio desulfuricans YP_388897
YdgE Deinococcus geothermalis YP_605420
YdgE Helicobacter hepaticus NP_860039
YdgE Campylobacter jejuni YP_179295
YdgE Pseudoalteromonas haloplanktis YP_340953
YvaD Anabaena variabilis YP_323732
YvaD Nostoc sp. PCC NP_487548
YvaD Chloroflexus aurantiacus ZP_00765654

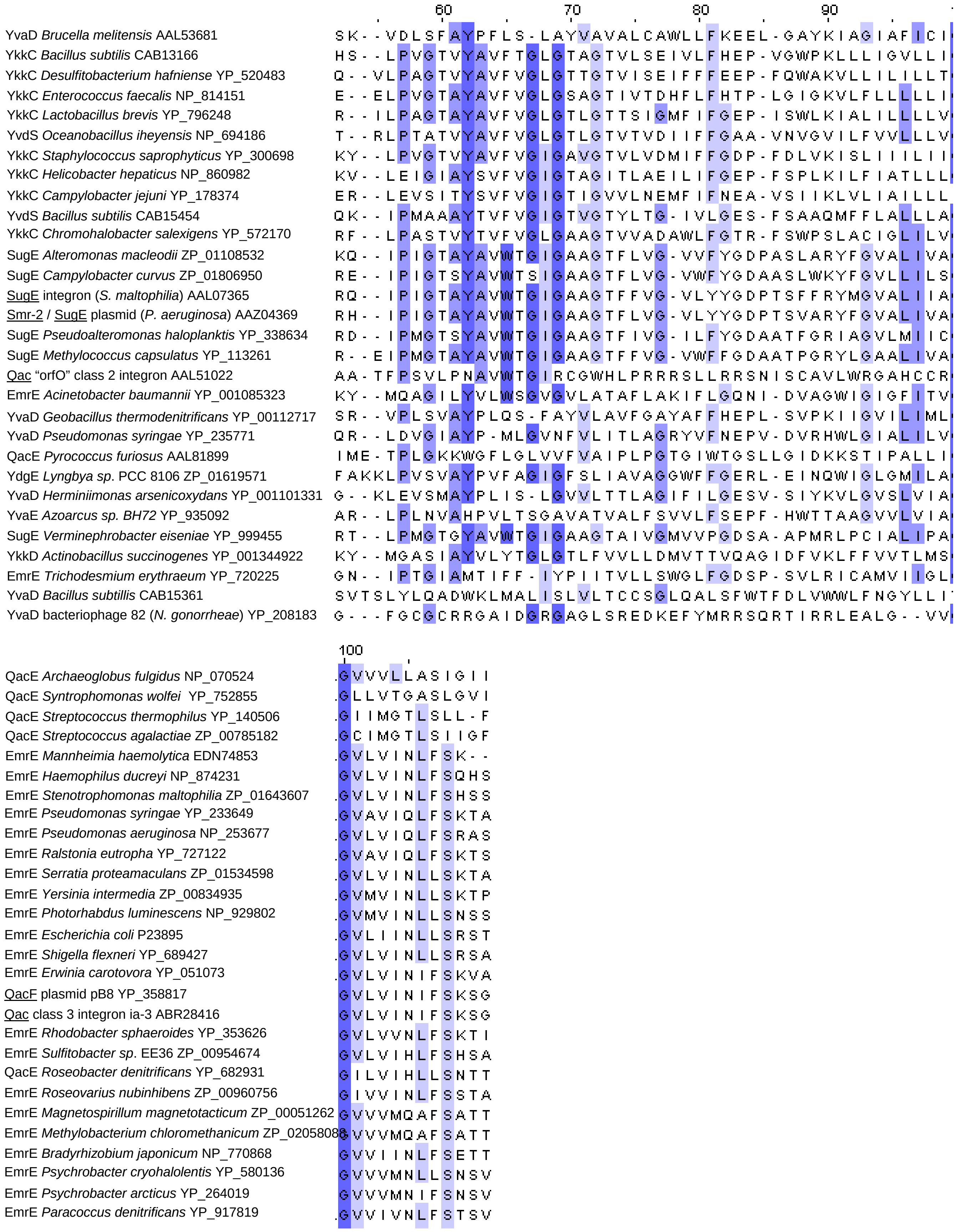

60 70 80 90
YvaD Brucella melitensis AAL53681 SK--VDLSFAYPFLS-LAYVAVALCAWLLFKEEL-GAYKIAGIAFICI
YkkC Bacillus subtilis CAB13166 HS--LPVGTVYAVFTGLGTAGTVLSEIVLFHEP-VGWPKLLLIGVLLI
YkkC Desulfitobacterium hafniense YP_520483 Q--VLPAGTVYAVFVGLGTTGTVISEIFFFEEP-FQWAKVLLILILLT
YkkC Enterococcus faecalis NP_814151 E--ELPVGTAYAVFVGLGSAGTIVTDHFLFHTP-LGIGKVLFLLLLLI
YkkC Lactobacillus brevis YP_796248 R--ILPAGTAYAVFVGLGTLGTTSIGMFIFGEP-ISWLKIALILLLLV
YvdS Oceanobacillus iheyensis NP_694186 T--RLPTATVYAVFVGLGTLGTVTVDIIFFGAA-VNVGVILFVVLLLV
YkkC Staphylococcus saprophyticus YP_300698 KY--LPVGTVYAVFVGIGAVGTVLVDMIFFGDP-FDLVKISLIIILII
YkkC Helicobacter hepaticus NP_860982 KV--LEIGIAYSVFVGIGTAGITLAEILIFGEP-FSPLKILFIATLLL
YkkC Campylobacter jejuni YP_178374 ER--LEVSITYSVFVGIGTIGVVLNEMFIFNEA-VSIIKLVLIAILLL
YvdS Bacillus subtilis CAB15454 QK--IPMAAAYTVFVGIGTVGTYLTG-IVLGES-FSAAQMFFLALLLA
YkkC Chromohalobacter salexigens YP_572170 RF--LPASTVYTVFVGLGAAGTVVADAWLFGTR-FSWPSLACIGLILV
SugE Alteromonas macleodii ZP_01108532 KQ--IPIGTAYAVWTGIGAAGTFLVG-VVFYGDPASLARYFGVALIVA
SugE Campylobacter curvus ZP_01806950 RE--IPIGTSYAVWTSIGAAGTFLVG-VWFYGDAASLWKYFGVLLILS
SugE integron (S. maltophilia) AAL07365 RQ--IPIGTAYAVWTGIGAAGTFFVG-VLYYGDPTSFFRYMGVALIIA
Smr-2 / SugE plasmid (P. aeruginosa) AAZ04369 RH--IPIGTAYAVWTGIGAAGTFLVG-VLYYGDPTSVARYFGVALIVA
SugE Pseudoalteromonas haloplanktis YP_338634 RD--IPMGTSYAVWTGIGAAGTFIVG-ILFYGDAATFGRIAGVLMIIC
SugE Methylococcus capsulatus YP_113261 R--EIPMGTAYAVWTGIGAAGTFFVG-VWFFGDAATPGRYLGAALIVA
Qac “orfO” class 2 integron AAL51022 AA-TFPSVLPNAVWTGIRCGWHLPRRRSLLRRSNISCAVLWRGAHCCR
EmrE Acinetobacter baumannii YP_001085323 KY--MQAGILYVLWSGVGVLATAFLAKIFLGQNI-DVAGWIGIGFITV
YvaD Geobacillus thermodenitrificans YP_00112717 SR--VPLSVAYPLQS-FAYVLAVFGAYAFFHEPL-SVPKIIGVILIML
YvaD Pseudomonas syringae YP_235771 QR--LDVGIAYP-MLGVNFVLITLAGRYVFNEPV-DVRHWLGIALILV
QacE Pyrococcus furiosus AAL81899 IME-TPLGKKWGFLGLVVFVAIPLPGTGIWTGSLLGIDKKSTIPALLI
YdgE Lyngbya sp. PCC 8106 ZP_01619571 FAKKLPVSVAYPVFAGIGFSLIAVAGGWFFGERL-EINQWIGLGMILA
YvaD Herminiimonas arsenicoxydans YP_001101331 G--KLEVSMAYPLIS-LGVVLTTLAGIFILGESV-SIYKVLGVSLVIA
YvaE Azoarcus sp. BH72 YP_935092 AR--LPLNVAHPVLTSGAVATVALFSVVLFSEPF-HWTTAAGVVLVIA
SugE Verminephrobacter eiseniae YP_999455 RT--LPMGTGYAVWTGIGAAGTAIVGMVVPGDSA-APMRLPCIALIPA
YkkD Actinobacillus succinogenes YP_001344922 KY--MGASIAYVLYTGLGTLFVVLLDMVTTVQAGIDFVKLFFVVTLMS
EmrE Trichodesmium erythraeum YP_720225 GN--IPTGIAMTIFF-IYPIITVLLSWGLFGDSP-SVLRICAMVIIGL
YvaD Bacillus subtillis CAB15361 SVTSLYLQADWKLMALISLVLTCCSGLQALSFWTFDLVWWLFNGYLLI
YvaD bacteriophage 82 (N. gonorrheae) YP_208183 G---FGCGCRRGAIDGRGAGLSREDKEFYMRRSQRTIRRLEALG--VV
100
QacE Archaeoglobus fulgidus NP_070524 .GVVVLLASIGII
QacE Syntrophomonas wolfei YP_752855 .GLLVTGASLGVI
QacE Streptococcus thermophilus YP_140506 .GIIMGTLSLL-F
QacE Streptococcus agalactiae ZP_00785182 .GCIMGTLSIIGF
EmrE Mannheimia haemolytica EDN74853 .GVLVINLFSK--
EmrE Haemophilus ducreyi NP_874231 GVLVINLFSQHS
EmrE Stenotrophomonas maltophilia ZP_01643607 .GVLVINLFSHSS
EmrE Pseudomonas syringae YP_233649 .GVAVIQLFSKTA
EmrE Pseudomonas aeruginosa NP_253677 .GVLVIQLFSRAS
EmrE Ralstonia eutropha YP_727122 .GVAVIQLFSKTS
EmrE Serratia proteamaculans ZP_01534598 .GVLVINLLSKTA
EmrE Yersinia intermedia ZP_00834935 .GVMVINLLSKTP
EmrE Photorhabdus luminescens NP_929802 .GVMVINLLSNSS
EmrE Escherichia coli P23895 .GVLIINLLSRST
EmrE Shigella flexneri YP_689427 .GVLVINLLSRSA
EmrE Erwinia carotovora YP_051073 .GVLVINIFSKVA
QacF plasmid pB8 YP_358817 GVLVINIFSKSG
Qac class 3 integron ia-3 ABR28416 GVLVINIFSKSG
EmrE Rhodobacter sphaeroides YP_353626 GVLVVNLFSKTI
EmrE Sulfitobacter sp. EE36 ZP_00954674 GVLVIHLFSHSA
QacE Roseobacter denitrificans YP_682931 GILVIHLLSNTT
EmrE Roseovarius nubinhibens ZP_00960756 GIVVINLFSSTA
EmrE Magnetospirillum magnetotacticum ZP_00051262 GVVVMQAFSATT
EmrE Methylobacterium chloromethanicum ZP_02058088 VVVMQAFSATT
EmrE Bradyrhizobium japonicum NP_770868 GVVIINLFSETT
EmrE Psychrobacter cryohalolentis YP_580136 GVVVMNLLSNSV
EmrE Psychrobacter arcticus YP_264019 GVVVMNIFSNSV
EmrE Paracoccus denitrificans YP_917819 .GVVIVNLFSTSV

	100
EmrE *Desulfovibrio desulfuricans* YP_388842	GIVVINVFSKTV
EmrE *Photobacterium profundum* ZP_01218031	.GTVIINVFSNTT
EmrE *Vibrio harveyi* YP_001445675	GVMIVNLLSKTV
EmrE *Xanthobacter autotrophicus* YP_001417989	.GVMVINLLSKSV
EmrE *Sphingomonas wittichii* YP_001263498	.GVAVLNLFSTSI
EmrE *Acidovorax avenae subsp. citrulli* YP_973010	.GVLVINLFSSTA
EmrE *Delftia acidovorans* ZP_01577698	.GVLVIQLFSKTA
EmrE *Myxococcus xanthus* YP_633339	.GVIVINTFSRSA
EmrE *Dechloromonas aromatica* YP_283677	.GVVVLNVFSKTV
QacE *Xanthomonas campestris* NP_637776	.GAVVINVFSKMA
EmrE *Burkholderia cepacia* YP_773352	.GVVVLNLFSKMQ
Qac intI1 (*Serratia marcescens*) AAK40353	GVAVLNPLSKVS
QacF intI (*Achromobacter denitrificans*) AAZ14837	GVAVLNLLSKVS
QacF plasmid pIP833 (*E. aerogenes*) AAD22143	GVAVLNLLSKVS
QacH plasmid p3iANG (*Vibrio cholerae*) AAZ42322	GVAVLNLLSKVS
QacF plasmid pSC138 (*S. enterica*) AAX56371	GVAVLNLLSKVS
QacE2 Int Class1 (*A. salmonicida*) AAK53557	GVAVLNLLSKAS
QacG class I intergon (*A. baumannii*) AAL38576	GVAVLNLLSKAS
QacG integron (*P. aeruginosa*) AAS79147	GVAVLNLLSKAS
QacE plasmid R751(*E. aerogenes*) NP_044260	GVVVLNLLSKAS
QacEΔ1 class 2 integron (*P. aeruginosa*) AAV32841	.AFLLARSPSWKS
EmrE *Marinobacter aquaeolei* YP_958340	GVLVINLLSNTG
Smr *Chloroflexus aurantiacus* ZP_00766077	GVVILNLLSKVE
EmrE *Shewanella frigidimarina* YP_748773	GVVVMNLFSNVT
EmrE *Chromobacterium violaceum* NP_902350	.GVAVINLFSHSA
EmrE *Methylobacillus flagellatus* YP_544992	.GVVVMQLFSRTT
EmrE *Methylococcus capsulatus* YP_115175	.GVLVCTLWSKSA
EmrE *Coxiella burnetii* ZP_01299513	GVLVINLLSKSI
QacH *Brucella melitensis* YP_414370	.GVIVLNLLSNTA
QacE *Brucella melitensis* AAL52226	.GVIVLNLLSNTA
EmrE *Ochrobactrum anthropi* YP_001370804	.GVIVLNLFSNTA
EmrE *Marinobacter algicola* ZP_01894487	.GVMVINVFSGSI
EmrE *Nitrosococcus oceani* YP_342650	.GVVVLNVFSKSI
EmrE *Reinekea sp.* MED297 ZP_01116498	GVVVLNLFSNVG
EmrE *Geobacter sulfurreducens* NP_951765	.GVLVLNLCSTSA
EmrE *Chlorobium limicola* EAM42528	GVLVLNLFSKTV
EmrE *Alcanivorax borkumensis* YP_693512	GVVLVTLVSNTA
EmrE *Jannaschia sp. CCS1* YP_511013	GVLILRLMTTTA
EmrE *Chromohalobacter salexigens*	.GVVIIQAFSRMS
QacH *Pelagibacter ubique* ZP_01264056	GVLMVNLLGNN-
QacH2 *Pelagibacter ubique* YP_266208	GVILLNTMGKTN
EmrE *Pseudoalteromonas atlantica* YP_661014	GVAMINLSQGHS
QacF *Pseudoalteromonas haloplanktis* CAI87270	GIAMINLSQGHS
Qac megaplasmid (*S. oneidensis)* NP_720475	GVIMINLSQGHS
YvaE *Shewanella frigidimarina* YP_752098	GVVMINLSQGHS
EmrE *Shewanella pealeana* YP_001500134	GVMMINFSQGHA
EmrE *Vibrio harveyi* YP_001449011	GVMVINFSHGHA
EmrE *Photobacterium profundum* ZP_01219483	GIAIINLSSVPH
EbrB *Pseudomonas syringae* YP_235623	GIILVNLAKEQA
EmrE *Wigglesworthia glossinidia* NP_871589	GVLIINIY----
EmrE *Aeromonas hydrophila* YP_855864	GGVIMNVFSSM-
EmrE *Erwinia carotovora* YP_051860	GGVIMNLYSSM-
EmrE *Aeromonas hydrophila* YP_855865	GIVIMNLFS---
QacE integron (*Escherichia coli*) YP_672432	GIIIMNLFSKMG
QacH *Erwinia carotovora* YP_051859	GIMIMNLFSK--
EmrE *Nitrobacter winogradskyi* YP_319204	GAVGLNLTTSVS
EmrE *Synechococcus sp.* RS9917 ZP_01081426	GVVIVNLTGSLE
EbrB *Mannheimia haemolytica* EDN74090	GVIMMNTLSKMG
EmrE *Neisseria meningitidis* NP_284788	GVVLLNTMSHMS

Sequence	100
EbrB *Listeria monocytogenes* EAL09800	GVIILNQRSADT
EbrA *Paenibacillus larvae* ZP_02326984	GVFFLNKSKEKG
EbrB *Bacillus subtillis* CAB13613	GVVVLNQSKAHA
EbrB *Paenibacillus larvae* ZP_02326985	GVFILNL-----
EbrA *Bacillus subtillis* CAB13614	GVVLLNWP----
QacG plasmid pST94 O87866	GVVLLNVFGESH
QacJ plasmid pNVH01 (*S. aureus*) CAD55144	GVVLLNVFGSSH
QacH plasmid pST2H6 (*S. saprophyticus*) CAA76544	GVVLLNTFGSSH
Smr *Staphylococcus aureus* AAM94142	GVVSLNIFGTSH
EbrA *Desulfotomaculum reducens* YP_001114614	GVIILNL-----
Ebr *Pediococcus pentosaceus* YP_804449	GVILVNLFGVS-
Ebr1 *Synechococcus sp*. RS9917 ZP_01080450	GVMISNYHGLVE
EbrA *Listeria monocytogenes* EAL09801	GVVVLKL-----
Smr *Roseiflexus castenholzii* ZP_01530339	GVVLLNMFGGSE
EbrA *Roseiflexus sp*. RS-1 YP_001278866	GVILLNMFGGE-
EbrA *Gloeobacter violaceus* NP_927270	GVLVLNLSGGGR
EbrB *Thermus thermophilus* YP_004540	GVVLIRLAD---
EbrB *Halobacterium sp*. NRC1 NP_444228	GVVVLNVASDAY
EmrE *Zymomonas mobilis sbsp. mobilis* YP_161843	GVIGLNLISPA-
Smr2 *Mycobacterium vanbaalenii* YP_954149	GVVLVESGSHQE
Smr2 *Mycobacterium gilvum* YP_001134816	GVALVETGSRVD
EbrB *Clavibacter michiganensis* YP_001220961	GVLLIELGH---
Smr *Nocardia farcinica* YP_118186	GVLALELGGAH-
Smr *Saccharopolyspora erythraea* YP_001109297	GVLALEM-----
Smr *Thermobifida fusca* YP_289048	GVAALELGAAR-
Smr *Saccharopolyspora erythraea* YP_001103712	GVLSLELGGQH-
Smr *Nocardioides sp*. JS614 YP_921842	GVLALELGARHG
Smr *Nocardioides sp*. JS614 YP_921842	GVVTLQLDGHQA
Smr *Mycobacterium tuberculosis* P95094	GVVTLNLAGAH-
NepA plasmid pAO1 (*A. nicotinovorans*) CAD47919	GVVTLNLQAGQ-
EmrE *Candidatus Pelagibacter ubique* ZP_01264207	GVVLVNSFTIKT
QacH *Lactobacillus plantarum* NP_786542	GAILLN---I--
YdgF *Escherichia coli* ABE07266	GIVLIKSGTRKA
YdgF *Shigella flexneri* YP_689093	GIVLIKSGTRKA
YdgF *Salmonella enterica sbsp. enterica* YP_216487	GIVLIKSGTRKP
YdgF *Yersinia intermedia* ZP_00833156	GIMLVKSGTRKP
YdgF *Photorhabdus luminescens* NP_929383	GIMLIKSGTRKA
YdgF *Pseudomonas aeruginosa* NP_250232	GILLIKSGTRNA
YdgF *Sinorhizobium meliloti* NP_385720	GILLLNAGRSDA
YdgF *Aeromonas hydrophila* YP_857997	GIVLVTMGETHD
YdgF *Vibrio harveyi*	GIVCVTL-----
YdgF *Chromobacterium violaceum* NP_900949	GLVCVTMGETHD
YdgF *Campylobacter jejuni* YP_179294	GIIMINIGEVKE
YdgF *Desulfovibrio desulfuricans* YP_388898	GVLLVH------
YdgF *Lawsonia intracellularis* CAJ54979	GAILIHHGTVNI
YdgF *Deinococcus geothermalis* YP_605419	GARLLHGGTRAR
YdgF *Pseudoalteromonas haloplanktis* YP_340952	GIVIINSGHAMQ
YdgF *Acidovorax avenae subsp. citrulli* YP_969092	GVVLVNADGAHA
YdgF *Helicobacter hepaticus* NP_860040	GIALINYAEIKR
EmrE *Alteromonas macleodii* ZP_01110205	GTLLITSKSAVV
YvaE *Bacillus subtilis* CAB15362	GVVSLNLIEHVA
YvaE *Blastopirellula marina* ZP_01094237	GVVGLNACS---
YvaE *Desulfitobacterium hafniense* YP_517233	GVVGLNLGGTA-
YvaE *Rhodospirillum rubrum* YP_428434	GVVGLNLLSKQ-
YvaE *Bdellovibrio bacteriovorus* NP_968626	GVVILNL-----
Smr *Streptomyces coelicolor* AAK95484	GVVVLNLGG---
YvaE *Acidobacteria bacterium* YP_593828	GVVGLNLSGRS-
EbrB *Rubrobacter xylanophilus* YP_644095	GVMLLNLSG---
YvaE *Desulfovibrio vulgaris* YP_966624	GVVGLNYTSSGE
YvaE *Pelodictyon phaeoclathratiforme* ZP_00590439	GVAGLHFSQEVH
YvaE *Chlorobium tepidum* NP_662342	GIAGLHFSQEHM

Sequence	100
YvaE *Prosthecochloris vibrioformis* YP_001130806	GVVGLQLSQHTA
YvaE *Anabaena variabilis* YP_323581	GVVGIN-SGK--
YvaE *Nostoc sp*. PCC 7120 NP_484329	GVVGIN-SGK--
EbrA *Methanosarcina acetivorans* NP_616284	GVIGLHLSDKVT
YvaE *Methanococcoides burtonii* YP_566929	GVLGLNLSDKLA
YvaE *Clostridium beijerinckii* YP_001307448	GVIGLNLTST--
YvaE *Marinomonas sp*. MED121 ZP_01075592	GVFGLHQVSTKT
QacC *Lactobacillus plantarum* NP_786541	GVILLNMPAKTT
NepB plasmid pAO1 (*A. nicotinovorans*) CAD47918	GIVILNLATTRQ
YvaE *Burkholderia cenocepacia* YP_839289	SLVLLNLSDGPT
EmrE *Synechococcus sp*. RS9917 ZP_01080451	GILVLNSSQQEA
SugE *Escherichia coli* AAC46453	GIIGLKLSTH--
SugE *Shigella flexneri* YP_691592	GIIGLKLSTH--
SugE *Salmonella enterica sbsp. enterica* YP_219204	GIIGLKLSAH--
SugE plasmid/ *Citrobacter freundii* AAC46457	GIIGLKLSTH--
SugE *Serratia proteamaculans* ZP_01538212	GILGLKFSSH--
SugE *Yersinia intermedia* ZP_00832613	GIIGLKLAS---
SugE *Photorhabdus luminescens* NP_931319	GIIGLKLSS---
SugE *Proteus vulgaris* P20928	GIIGLKLAS---
SugE *Pseudoalteromonas atlantica* YP_660982	GVIGLKLSS---
SugE *Limnobacter sp*. MED105 ZP_01915318	GVLGLKLS----
SugE *Shewanella oneidensis* NP_717508	GVLGLKLAH---
SugE *Methylobacillus flagellatus* YP_545743	GIIGLKLATP--
SugE *Bradyrhizobium japonicum* NP_770684	GIIGLKLVT---
SugE *Ralstonia eutropha* YP_727054	GIAGLKLVGSA-
SugE *Dechloromonas aromatica* YP_287101	GIIGLKLVTPDT
SugE *Mesorhizobium loti* NP_105639	GIMGLKFAA---
SugE *Rhizobium leguminosarum* YP_768145	GIAGLKLTA---
SugE *Sinorhizobium meliloti* NP_385492	GIAGLKLVG---
SugE *Herminiimonas arsenicoxydans* YP_001098437	GIIGLKVFSPT-
SugE *Gloeobacter violaceus* NP_925933	GIVGLKWVAPH-
SugE *Syntrophus aciditrophicus* YP_462525	GIVGLKLTTQS-
SugE *Geobacter sulfurreducens* NP_951764	GIIGLKLSSPA-
SugE *Pseudomonas aeruginosa* NP_251955	GLVGLKLSH---
SugE *Aeromonas hydrophila sp. hydrophila* YP_856475	GILGLKFIG---
SugE *Bdellovibrio bacteriovorus* NP_969313	SIIGLKATAGGH
SugE *Myxococcus xanthus* YP_631852	SIVGLKVTSGR-
SugE *Deinococcus geothermalis* YP_605633	AIIGLKATSGH-
SugE *Streptomyces coelicolor* NP_627124	AVVGLKATSGH-
SugE *Anaeromyxobacter dehalogenans* YP_467455	SILGLKLTAAS-
SugE *Blastopirellula marina* ZP_01088886	SIIGLKLTSGH-
SugE *Myxococcus xanthus* YP_628656	SVIGLKFTSGGD
SugE *Legionella pneumophila* YP_122951	SIFGLKYTA---
SugE *Marinomonas sp*. MWYL1 ZP_01596466	GIVGLKLTSS--
SugE *Vibrio harveyi* YP_001448000	GI----------
SugE *Rhodospirillum rubrum* YP_425364	GLIGLKIG----
SugE *Pelodictyon phaeoclathratiforme* ZP_00588597	GVIGLKIFSGGM
SugE *Chlorobium limicola* EAM42493	GVAGLRLFSR--
SugE *Prosthecochloris aestuarii* EAN23020	GVLGLRLL----
SugE *Chlorobium tepidum* NP_661278	GVAGLRVLAGK-
SugE *Geobacillus thermodenitrificans* YP_001124648	GIIGLKFTAGQ-
SugE *Desulfotomaculum reducens* YP_001114341	GMIGLKITSGH-
SugE *Methanosarcina barkeri* AAZ72469	GILGLRIISL--
SugE *Clostridium beijerinckii* YP_001310526	GIVGLKIVSSH-
SugE *Nitrobacter winogradskyi* YP_318926	GMVGLKVVSPA-
SugE *Rhodopirellula baltica* NP_869718	GIVGLKLASSS-
SugE *Nitrosococcus oceani* YP_344225	GIIGLKLTSSD-
SugE *Roseovarius nubinhibens* ZP_00959799	GIVGLKLVDGSA
SugE *Rhodobacter sphaeroides* YP_351822	GIIALKLA----

	100
SugE *Stenotrophomonas maltophilia* ZP_01645236	GVIGLKLVSPN-
SugE *Xanthomonas campestris* NP_635868	GVIGLKLVS---
SugE *Symbiobacterium thermophilum* YP_074555	GIVGLKLTSRS-
SugE *Xanthobacter autotrophicus* YP_001419080	GIVGLKLVTP--
SugE *Brucella melitensis biovar abortus* YP_414234	GIIGLKLSST--
SugE *Ochrobactrum anthropi* YP_001371057	GIIGLKLSSS--
SugE *Burkholderia cepacia* YP_774210	GLVGLKLSSSA-
SugE *Zymomonas mobilis sp. mobilis* YP_162432	GIILMKLSNKNV
SugE *Stenotrophomonas maltophilia* ZP_01643044	GLVLMKLSSS--
SugE *Delftia acidovorans* ZP_01582139	GLVLMKLSSD--
SugE *Magnetospirillum magnetotacticum* ZP_00208705	GLVLMKLSSPA-
SugE *Methylobacterium sp.* 4-46 ZP_01845840	GLVTMKLASTS-
SugE *Nitrosomonas europaea* NP_842203	GLVLMKLSS---
SugE *Acinetobacter baumannii* YP_001083756	GLVLMKISSPS-
SugE *Paracoccus denitrificans* YP_917710	GLVLMKIASPH-
SugE *Pseudomonas aeruginosa* NP_250573	GIVILKLATRQG
SocA2 *Myxococcus xanthus* YP_633361	GVAGLRAIER--
SugE *Bordetella parapertussis* NP_883309	GVAGLRMIEP--
SugE *Ralstonia eutropha* YP_726368	GIGGLKLLPA--
SugE2 *Mycobacterium gilvum* YP_001136522	SVVGLKAVS---
SugE *Clavibacter michiganensis* YP_001223477	CVVGLKVVDTGH
SugE1 *Arthrobacter aurescens* YP_949237	CVVGLKVVA---
SugE *Arthrobacter aurescens* YP_949238	CAAGLKALPAEK
SugE1 *Mycobacterium gilvum* YP_001136521	AVAGLKVLPSST
SugE *Kineococcus radiotolerans* ZP_00617403	GVVGLKFTH---
SugE *Nocardia farcinica* YP_116350	GVVGLKMVG---
SugE *Thermobifida fusca* YP_289322	GIVGLRVLE---
SugE *Stigmatella aurantiaca* ZP_01464276	GVTGLALTGGGH
SugE *Saccharopolyspora erythraea* YP_001109242	GVIGLNLSGAAH
SugE *Streptomyces coelicolor* NP_629357	GVIGLQLSGSAH
SugE1 *Listeria monocytogenes* YP_013473	GIIGLKLVDGNE
SugE *Bradyrhizobium japonicum* NP_770973	GIAALKLA----
SugE *Burkholderia cepacia* YP_776179	GIVGLQLQEGV-
SugE *Sphingopyxis alaskensis* YP_615530	GVAGTKLFAAN-
SugE *Alcanivorax borkumensis* YP_694264	GIVGLKLTSGPA
SugE *Nocardia farcinica* YP_120693	GIVLARLTNPE-
SugE *Protochlamydia amoebophila* YP_007714	SIIGLKISSA--
SugE *Leptospira borgpetersenii* YP_801667	SVLGLKFLVSE-
SugE *Rickettsia bellii* YP_537322	SIIGLKFS----
SugE *Bacteroides ovatus* ZP_02066060	SIVGLKL-----
SugE *Flavobacterium johnsoniae* YP_001195630	SIVGLKAVSH--
SugE *Rickettsiella grylli* ZP_01300129	SIFGLKLVSSR-
SugE *Sphingomonas wittichii* YP_001262619	CIAGLKLTAH--
SugE2 *Deinococcus radiodurans* AAF10580	SIAGLKIVDR--
SugE1 *Deinococcus radiodurans* AAF10579	AILGLKVTA---
SugE *Rubrobacter xylanophilus* YP_643069	GVLMLRFAEA--
SugE2 *Listeria monocytogenes* YP_013474	GVVGLKLTSGV-
SugE *Pediococcus pentosaceus* YP_803585	GIIGIKMTAGH-
SugE *Lactobacillus plantarum* NP_784622	GIIGIKVTS---
SugE *Enterococcus faecium* ZP_00603219	SIIGIKITSGH-
SugE *Jannaschia sp.* CCS1 YP_509232	LIAGMFLLGSDA
YkkD *Bacillus subtilis* CAB13167	SAVGLKILS---
YkkD *Staphylococcus saprophyticus* YP_300697	SIVGLKIVS---
YkkD *Helicobacter hepaticus* NP_860983	SVVGLKALS---
YkkD *Campylobacter jejuni* YP_178373	CTF----LS---
YkkD *Desulfitobacterium hafniense* YP_520482	SVIGLKLIS---
YkkD *Lactobacillus brevis* YP_796249	ATLGLKLIS---
YkkD *Enterococcus faecalis* NP_814152	SVIGLKLIG---
YvdR *Oceanobacillus iheyensis* NP_694185	AVIGLRIVSN--
YkkD *Chromohalobacter salexigens* YP_572171	GVVGLKLLA---
YkkC *Actinobacillus succinogenes* YP_001344921	SAIALKVM----

	100
Sug2 *Wolbachia pipientis* NP_965923	GIIGLKLFDTAK
Sug1 *Wolbachia pipientis* NP_965922	GSIGLKLSI---
YvdR *Bacillus subtilis* CAB15455	GVIGLRLTSSS-
SugE *Anaeromyxobacter dehalogenans* YP_467311	GVVGLRLSGAA-
SugE *Pseudomonas aeruginosa* NP_249477	GVAGLK------
YdgE *Escherichia coli* YP_540796	GMIMVKLA----
YdgE *Shigella flexneri* YP_689092	GMIMVKLA----
YdgE *Salmonella enterica* YP_216488	GMVMIKFA----
YdgE *Yersinia intermedia* ZP_00833155	GMVMIKLS----
YdgE *Photorhabdus luminescens* NP_929384	GMVMIKMA----
YdgE *Pseudomonas aeruginosa* NP_250231	GMSLLKLA----
YdgE *Acidovorax avenae subsp. citrulli* YP_969095	GISLLKLS----
YdgE *Burkholderia dolosa* ZP_00983437	AVVLLSTAE---
YdgE *Chromobacterium violaceum* NP_900948	AVVTLSTV----
YdgE *Stenotrophomonas maltophilia* ZP_01645705	AVLLLSTAK---
YdgE *Aeromonas hydrophila* YP_857998	AVVVM-------
YdgE *Vibrio harveyi* ZP_01986465	AVTLLTTA----
YdgE *Sinorhizobium meliloti* NP_385721	AVLVLHTA----
YdgE *Desulfovibrio desulfuricans* YP_388897	GLAMLHIS----
YdgE *Deinococcus geothermalis* YP_605420	SVAVLHLAEGSQ
YdgE *Helicobacter hepaticus* NP_860039	GVILLHWES---
YdgE *Campylobacter jejuni* YP_179295	AVFLLNY-----
YdgE *Pseudoalteromonas haloplanktis* YP_340953	SIVTMQLVGPA-
YvaD *Anabaena variabilis* YP_323732	GISAIALAKPS-
YvaD *Nostoc sp. PCC* NP_487548	GISAIALSKPS-
YvaD *Chloroflexus aurantiacus* ZP_00765654	GMLMVARS----
YvaD *Brucella melitensis* AAL53681	GTILIAQSGKVR
YkkC *Bacillus subtilis* CAB13166	GVIGLKLVTQDE
YkkC *Desulfitobacterium hafniense* YP_520483	GVFGLKAVTGER
YkkC *Enterococcus faecalis* NP_814151	GIIGLKMVTGNK
YkkC *Lactobacillus brevis* YP_796248	GIGGLQWTTTKE
YvdS *Oceanobacillus iheyensis* NP_694186	GVLGLKSVTG--
YkkC *Staphylococcus saprophyticus* YP_300698	GIIGLKLTTEEA
YkkC *Helicobacter hepaticus* NP_860982	GVVGLKLSADKT
YkkC *Campylobacter jejuni* YP_178374	SIIALKWVS---
YvdS *Bacillus subtilis* CAB15454	GILGMKLFTKES
YkkC *Chromohalobacter salexigens* YP_572170	GVIGLHQFSGKP
SugE *Alteromonas macleodii* ZP_01108532	GVVVLKLAH---
SugE *Campylobacter curvus* ZP_01806950	GVVVLKVA----
<u>SugE</u> integron (*S. maltophilia*) AAL07365	GVITLKLAH---
<u>Smr-2</u> / <u>SugE</u> plasmid (*P. aeruginosa*) AAZ04369	GVITLKLAH---
SugE *Pseudoalteromonas haloplanktis* YP_338634	GVITLKISH---
SugE *Methylococcus capsulatus* YP_113261	GVMTLKLAH---
<u>Qac</u> “orfO” class 2 integron AAL51022	GARALRCGR---
EmrE *Acinetobacter baumannii* YP_001085323	GLMIIAQYY---
YvaD *Geobacillus thermodenitrificans* YP_00112717	GVSFIGFSANGS
YvaD *Pseudomonas syringae* YP_235771	GVFQLGRQA---
QacE *Pyrococcus furiosus* AAL81899	GLLSLAITTLPT
YdgE *Lyngbya sp.* PCC 8106 ZP_01619571	GIIIMSR-----
YvaD *Herminiimonas arsenicoxydans* YP_001101331	GVLVLSVKS---
YvaE *Azoarcus sp. BH72* YP_935092	GVFLITFRVA--
SugE *Verminephrobacter eiseniae* YP_999455	GVIGFKRVTSDG
YkkD *Actinobacillus succinogenes* YP_001344922	GVIVIKSAK---
EmrE *Trichodesmium erythraeum* YP_720225	GILVIPGGVGN-
YvaD *Bacillus subtillis* CAB15361	YLFFIHLLLKEG
YvaD bacteriophage 82 (*N. gonorrheae*) YP_208183	GVK--RLK----
